# An inaugural forum on epidemiological modeling for public health stakeholders in Arizona

**DOI:** 10.3389/fpubh.2024.1357908

**Published:** 2024-05-31

**Authors:** Joseph R. Mihaljevic, Carmenlita Chief, Mehreen Malik, Kayode Oshinubi, Eck Doerry, Esma Gel, Crystal Hepp, Tim Lant, Sanjay Mehrotra, Samantha Sabo

**Affiliations:** ^1^School of Informatics, Computing, and Cyber Systems, Northern Arizona University, Flagstaff, AZ, United States; ^2^Center for Health Equity Research, College of Health and Human Services, Northern Arizona University, Flagstaff, AZ, United States; ^3^Interdisciplinary Health Program, College of Health and Human Services, Northern Arizona University, Flagstaff, AZ, United States; ^4^Department of Supply Chain Management and Analytics, University of Nebraska-Lincoln, Lincoln, NE, United States; ^5^Pathogen and Microbiome Division, Translational Genomics Research Institute, Flagstaff, AZ, United States; ^6^Office of the Vice President for Research, Knowledge Enterprise, Arizona State University, Tempe, AZ, United States; ^7^Department of Industrial Engineering and Management Sciences, Northwestern University, Evanston, IL, United States; ^8^Center for Engineering and Health, Feinberg School of Medicine, Northwestern University, Chicago, IL, United States

**Keywords:** public health, stakeholder engagement, predictive modeling, collective knowledge building, infectious disease epidemiology

## Abstract

Epidemiological models—which help us understand and forecast the spread of infectious disease—can be valuable tools for public health. However, barriers exist that can make it difficult to employ epidemiological models routinely within the repertoire of public health planning. These barriers include technical challenges associated with constructing the models, challenges in obtaining appropriate data for model parameterization, and problems with clear communication of modeling outputs and uncertainty. To learn about the unique barriers and opportunities within the state of Arizona, we gathered a diverse set of 48 public health stakeholders for a day-and-a-half forum. Our research group was motivated specifically by our work building software for public health-relevant modeling and by our earnest desire to collaborate closely with stakeholders to ensure that our software tools are practical and useful in the face of evolving public health needs. Here we outline the planning and structure of the forum, and we highlight as a case study some of the lessons learned from breakout discussions. While unique barriers exist for implementing modeling for public health, there is also keen interest in doing so across diverse sectors of State and Local government, although issues of equal and fair access to modeling knowledge and technologies remain key issues for future development. We found this forum to be useful for building relationships and informing our software development, and we plan to continue such meetings annually to create a continual feedback loop between academic molders and public health practitioners.

## 1 Introduction

Quantitative models that describe the spread of infectious disease within populations (i.e., epidemiological models) have become widely appreciated for their ability to provide a quantitative, data-driven foundation for evaluating complex decisions that emerge during epidemics (1–4). Epidemiological models can provide short-term forecasts of disease burden as well as scenario modeling, both of which are useful for the planning and evaluation of public health interventions to mitigate disease spread. For example, models are commonly used to forecast the severity of the influenza season and have been used to analyze the potential effects of population-scale health interventions, such as vaccinations, against a myriad of diseases (5–7).

Epidemiological modeling has emerged as a vital tool during the COVID-19 pandemic, caused by the novel coronavirus SARS-CoV-2. Throughout the COVID-19 pandemic, the US Centers for Disease Control and Prevention (CDC) advertised models that provide short-term forecasts of COVID-19 cases, hospitalizations, and mortalities at the state or country level (2, 8). COVID-19 model predictions were discussed on national news networks and in US White House press briefings (9, 10). Indeed, scenario modeling of SARS-CoV-2 dynamics was critical in helping state and federal governments explore and enact large-scale interventions, such as shelter-in-place orders, to combat transmission and avert overwhelmed hospital systems (3, 11–13), and models were also used to address the optimal allocation of vaccines when they became available (14).

Although providing model-based understanding and forecasts at the state and national spatial scales can be useful for overall public health planning in the midst of outbreaks, the leadership of local municipalities (including counties, cities, and tribal communities) may find it difficult to derive useful insights pertinent to their constituency (15, 16). If models make predictions at the national level, local leaders may not be sure if these predictions are relevant to their area. In past epidemics local policymakers have stated that recommendations based on state- or national-level models were not always useful in making decisions for their jurisdictions (17). Collaboration between local policymakers and epidemiological modelers must therefore be improved to lead to more actionable public health recommendations appropriate for local municipalities (15, 18, 19).

There are substantial challenges in designing and implementing epidemiological models that can impede the effective use of models in public health decision-making, but close collaborations could help overcome these challenges. Specifically, there are conceptual and technical challenges associated with constructing the models themselves, statistical challenges in tuning model parameters using local data, challenges in obtaining appropriate and accurate data for model parameterization, social challenges of organizing effective collaboration and communication between modeling experts and stakeholders, challenges in the design of modeling experiments, and finally, in clear communication of modeling outputs and implications to stakeholders with diverse backgrounds and priorities. More pragmatically, there are also the basic operational challenges of funding epidemiologic modeling and finding relevant experts. These challenges have been accentuated during the current SARS-CoV-2 pandemic, highlighting the need for new research, as well as the need for establishing new frameworks for collaboration and communication reaching from modelers to public health officials to local citizens and decision makers. In this context, identifying and analyzing challenges in local-level epidemiologic modeling is an opportunity to strategize about ways of overcoming challenges to local-level modeling as the CoV-2 pandemic continues and as future epidemics arise, with special emphasis on benefitting local governments and communities.

Collaborations between modeling experts and public health experts and other stakeholders can be critical for designing high quality epidemiological models that are clear, credible, and practical in applied settings. Developing the trust relationships and educational preparation that are the foundation for such collaborations can be challenging, but previous studies describe how targeted conferences, meetings, and forums can be useful mechanisms for building understanding and trust between stakeholders with different backgrounds, expertise, and priorities (20–24). For example, Moghadas and colleagues (21) gathered public health and policy experts to discuss pandemic preparedness in the context of influenza, emphasizing how mathematical models can help ground these conversations. Their workshop included modeling experts who gave presentations, educating the audience on the usefulness of models, followed by discussions to identify priorities for preparedness. The authors note how this workshop forged multidisciplinary collaborations and emphasize that such collaborations are needed to effectively translate modeling into policy recommendations.

Our goal was to design and host an inaugural forum on using quantitative modeling for public health planning in the state of Arizona. We specifically sought to build relationships, understanding, and trust between local modeling experts and stakeholders in state and local public health. This meeting was facilitated by research grants that aim to build software systems that translate modeling for public health planning. Our forum was held in part to help us begin building such software with collaborative input and deepen our understanding of the needs of public health partners in our state. Therefore, we designed the forum to include a mixture of information-sharing from modeling experts, in the form of keynote speeches, and from local leaders in public health, through breakout discussions that we guided. Here we describe how we organized and implemented our forum, as well as the insights we derived from keynote speakers and group discussions with forum participants.

## 2 Methods

Note that the Institutional Review Board (IRB) at Northern Arizona University determined that this study does not meet the definition of human subjects research. Our intent was to understand the attitudes of a group of Arizona stakeholders who might ultimately interact with our future modeling technologies, with the purpose of improving our technologies, communications, and future meetings with Arizona stakeholders specifically. We are viewing these stakeholders as a case study from a specific place, without the intent to generalize to the broader public.

### 2.1 Stakeholders engagement

Our goal was to host ~50 forum participants who engage with infectious disease research or public health service within the state of Arizona for a one-and-a-half-day forum. Therefore, we identified and invited multidisciplinary public health professional stakeholders within Arizona state government, county governments, tribal communities, and academic research institutions, but we also invited some select individuals from federal institutions, as well as our grant collaborators from out of state.

We used email marketing to engage with many stakeholders and provide information regarding forum registration and regular announcements such as keynote speakers, updated meeting agendas, and travel and lodging information. These emails included an RSVP link to an electronic registration form. We encouraged invitees who could not attend to identify an alternate colleague capable of representing their organization in the forum discussions.

### 2.2 Location of forum

The forum was hosted on the Mountain campus of Northern Arizona University in Flagstaff, Arizona, over 3 days, March 8–10, 2023. Before the official forum began, we hosted a reception on the evening of March 8 to welcome attendees and to encourage informal networking; about 60% of our participants attended. For the meetings, we had reserved a conference room on campus large enough to accommodate all 50 participants, but small enough to maximize participant engagement and network-building. Meals were provided. At the end of the first full day, we provided the option to go on a hike, although a large amount of snow on the ground discouraged all but a handful of participants. The Supplementary material includes a pamphlet that was distributed to participants, which outlines the goals of the forum.

### 2.3 Keynote speakers

Our aim was to invite diverse keynote speakers who could speak to the conceptual, technical, and social aspects of deploying mathematical modeling for public health responses. We particularly wanted speakers who had experience and deep involvement in the response to the COVID-19 pandemic, but also tapped individuals who conduct research on other diseases, such as vector-borne and sexually-transmitted infections, as these are established special interests for our Arizona audience. We asked the keynote speakers to discuss various practical aspects of infectious pathogen modeling, from creating models that integrate insights of local stakeholders, to testing hypotheses about pathogen transmission, to using models for planning resource allocation and deployment, and to generating forecasts.

#### 2.3.1 Day 1 keynote speakers

*Joseph Mihaljevic, Northern Arizona University*. Given the diverse backgrounds of the audience and variable levels of expertise with epidemiological models and the modeling process, Dr. Mihaljevic established a foundation for the forum with an Epidemiological Modeling 101 lecture. This talk started with the fundamentals of what mathematical models of infectious disease transmission represent and how individuals or teams work to construct these models, combining mathematics, computer science, and biology. The focus then shifted to how models can be useful in public health contexts: discovery of important mechanisms of transmission, forecasting, scenario simulation, and optimization of resource allocation for pathogen control. Finally, discussion shifted from theory to practice, exploring some of the complexities and challenges of modeling in real-world application scenarios, emphasizing that, while it is important to understand their limitations, models are a valuable source of information that can assist with decision-making in evolving epidemics.

*Subject Matter Expert A, “Cooperation and coordination of multiple models to manage the COVID-19 pandemic”. Subject Matter Expert A* is a leader in the field of epidemiological modeling for public health, focused their presentation on the importance of cooperation among modelers and the use of multiple models for robust uncertainty exploration. Beginning with an exploration of case studies from Ebola outbreaks, the speaker emphasized how different models can yield contradictory insights, and yet we can use formal model comparisons to understand in which aspects the models agree, providing stronger support for model outcomes. This led the speaker into a discussion of how cooperation among modelers during the COVID-19 pandemic led to the construction of the COVID-19 Scenario Hub, where several modeling groups produced standardized long-range scenario simulations under mutually agreed-upon criteria, such as how vaccination strategies would be applied in the next several weeks or months. The examples emphasized the utility of ensemble modeling, in which scenario (or forecast) simulations from multiple models are averaged, and this ensemble average can often outperform individual models. The speaker also discussed how this work was conveyed to national stakeholders and may have influenced national-level policy. A key takeaway was that designing models and scenario simulations with the input of decision makers and public health stakeholders is critical for making models useful in the very real context of a global pandemic.

*Two representatives from the Center for Forecasting and Outbreak Analytics (CFA), Centers for Disease Control and Prevention (CDC), “CDC Center for Forecasting and Outbreak Analytics 101”*. These two representatives presented a 15-min introduction to the administrative structure and vision of the newly formed CFA. They also previewed some funding mechanisms that were relevant to collaborations between modelers and federal, state, tribal, local, and territorial governments.

*Sanjay Mehrotra, Northwestern University, “Optimizing the Allocation of Ventilators for COVID-19”*. This talk helped our audience change gears and learn at a high level about how optimization theory is used to make informed decisions about resource allocation. Dr. Mehrotra introduced resource allocation as a challenge for public health, and then discussed a case-study about the allocation of ventilators in the early phase of the COVID-19 pandemic in the USA. Considering the interests of public health decision-makers, Dr. Mehrotra walked the audience through decision-support tools that his lab developed to visualize the distribution of ventilators across facilities. Dr. Mehrotra ended the talk discussing the hypothetical scenario of vaccine allocation across a diverse spatial landscape, using an Arizona county as a visual example. This talk spurred interesting discussion, as the optimal allocation of both vaccines and human resources was a consistent challenge for this audience during the pandemic, and some audience members pointed out how particular spatial heterogeneities unique to Arizona pose challenges for studies on resource allocation.

#### 2.3.2 Day 2 keynote speakers

*Subject Matter Expert B, “Participatory Design of Malaria Modeling Frameworks with Local Stakeholders: Experiences from Nigeria”*. The second day began with *Subject Matter Expert B*, who is an expert in working with local communities to design and implement model-guided interventions. The speaker introduced the audience to an interesting case-study of working with local stakeholders to build, test, and implement modeling of malaria transmission. Importantly, the speaker outlined how their research group used the modeling framework to assess intervention strategies, which ultimately helped inform national-level policy. This talk not only outlined how models can be used to simulate and evaluate competing intervention strategies, but also highlighted the complexities and benefits of working with stakeholders to define the goals of the modeling project, to obtain necessary data, and to communicate results in a way that builds trust in the modeling outcomes. This talk was a great way to contextualize our meeting, which was bringing together diverse perspectives to understand the opportunities and challenges of modeling for public health in our local communities.

*Subject Matter Expert C, “Predictive Modeling for Sexually-Transmitted and Vector-Borne Diseases”*. Motivated by the fact that many of the audience members work in the fields of vector-borne and sexually-transmitted infections, *Subject Matter Expert C* focused their presentation on the modeling of HIV epidemiology and mosquito-borne disease, introducing how models can integrate the effects of climate and local human environment on the dynamics of mosquitoes and mosquito-borne pathogens. The speaker focused on methodologies to define the model inputs, calibrate the models, and visualize the model outcomes (e.g., human risk of infection). The talk switched gears to using molecular epidemiology of HIV to understand outbreak clusters. Some key take-aways from this presentation were that bringing multiple streams of data together to inform a mathematical model can yield more useful and realistic model outcomes, and that feedback from public health experts is vital to construct useful modeling studies.

### 2.4 Breakout group discussions

Keynote presentations were followed by breakout-sessions to encourage discussion and build an understanding of our participants' viewpoints. Each of the three breakout sessions had a specific theme, focusing on (1) the participants' relationships with epidemiological data, (2) their utilization of data in decision making, and (3) the opportunities and challenges that participants see for epidemiological modeling in Arizona. More specifically, the first session started with brief introductions and then focused on the participants' exposure and use of infectious disease data, the spatial scale of data, opportunities for improving data literacy, data analysis tools and techniques, experience with mathematical modeling, and relevant challenges and opportunities for data. The focus of phase two was the participants' role in decision-making, the types of data involved in decision-making, opportunities for better integration of data into decision-making, and identifying unique issues specific to our partners' jurisdictions. Focus of the phase three discussion was the opportunities and challenges for applying epidemiological modeling for timely public health decision making, particularly in the state of Arizona. This last discussion took part on day two, after the participants had learned more about modeling from our keynote speakers.

During each breakout session, we mixed the participants into six groups of eight participants. Group membership was randomly reshuffled for each session to promote discussion across multiple disciplines and backgrounds. At the end of each 40-min session, each group assigned a representative to convey a summary of the group's most important points from the discussion. For each breakout session, handwritten notes were taken using a structured table top discussion guide, which was a set of pre-determined, open-ended questions developed by the project team. Project team members used the table top guide to facilitate a group discussion at each table. Group responses were summarized by question and major themes, lessons learned and recommendations were organized using a codebook (25). Project members independently read and coded the meeting notes and through a face-to-face process of consensus building agreed on patterns and themes for the table top discussions. Based on our experience with collaborative analysis (26, 27), themes were shared back with the broader project team for interpretation. The supplement provides high-level interpretation and identification of key words and themes collaboratively gleaned from the hand-written notes.

## 3 Results

### 3.1 Forum participant profile

A diverse group of 45 multidisciplinary stakeholders from health departments, universities and federal agencies participated in the forum (Table 1). Reflecting our focus on identifying the unique needs, barriers and opportunities of local communities in the application of predictive modeling, 26.6% (*n* = 12) of the participants were from the state and county health departments of Arizona; in addition, 15.6% (*n* = 7) were representatives of Tribal and Intertribal organizations.

**Table 1:** Forum participants by agency type.

**Agency type**	**Number of individuals**	**Percentage**
Northern Arizona University (NAU)	12	26.7
University/Research Institution (External to NAU)	12	26.7
State	4	8.9
County	8	17.7
Tribal and Intertribal Organization	7	15.6
Federal	2	4.4
Totals	45	100

### 3.2 Breakout group discussions

*Phase I: A focus on data*. During phase I discussion, stakeholders described their positionality with respect to infectious disease data, as well as their views on issues surrounding collection and analysis of various types of epidemiological data. Although participants' backgrounds varied widely in terms of which infectious diseases they worked with and types of data of highest priority to them, several common themes emerged regarding familiarity with data analysis tools and perceived limitations in using the data for analyses.

There were some positive commonalities among participants. For example, the use of ‘data dashboards' became much more common during the COVID-19 pandemic, which was generally seen as improving internal communications and improving the transfer of quantitatively-driven knowledge to the public. Also, across the group, there was a wide breadth of experience using different data processing and analysis tools, such as Excel, ArcGIS, SAS, internal databases, R, Python, SPSS, Tableau, and Javascript. There was also general excitement about using data in more advanced analytics, such as epidemiological modeling, but this led to a variety of points regarding the current challenges with data and analytics.

Some of these challenges have to do with the data quality. Participants mentioned problems with missing data and concerns regarding the reliability of existing data. Stakeholders who handle data described not always having information on who collected particular sets of data, who has access to data, or a detailed understanding of which data are most or least trustworthy. Stakeholders discussed a preference and need for data collected at fine spatial scales (e.g., zip codes or census block groups) for improved data modeling and predictive forecasting, and yet they see major gaps in the availability of reliable community-level data; particular example was weaknesses in tribe-specific data. Participants identified a lack of information about community demographics and social determinants of health, which makes it difficult to design effective control strategies for infectious diseases. These issues complicate the analysis of data and associated interpretation for timely actions (more on this below).

There are opportunities to improve data literacy and knowledge of current best practices in data analytics, including epidemiological modeling. Several stakeholders who are directly or indirectly involved in decision-making mentioned that they do not have structured opportunities to learn updated data analysis techniques and tools. Participants also discussed several barriers for analyzing data and conducting more advanced data analytics (e.g., mathematical modeling and forecasting), including a lack of a specialized workforce and a lack of funding for sophisticated analytic projects. Importantly, participants acknowledged the imminent need of capacity building in terms of training and skill building of the public health workforce for using predictive analytics. The discussion session concluded with a consensus that the establishment of infrastructures for the development and implementation of data analytics and modeling in the public health sector is a crucial way forward.

*Phase II: Data analysis for decision-making*. The focus of the second discussion session was on the participants' role in decision making, and the opportunities and challenges for enhancing the integration of data into decision making. Most of the stakeholders were not directly involved in decision making or policy making; instead, many participants were active in public health surveillance activities, which included providing data summaries and interpreting data for decision makers. There was a general understanding that data was key to decision-making: a lack of data and a lack of clear, easily interpretable data analytics (e.g., graphical summaries) can delay decision-making. Many challenges were identified that impeded a more robust integration of data into decision making.

Participants repeated many of the challenges identified in the first phase of discussions, including issues with data quality, as well as a lack of adequate funding and resources to carry out the required tasks of data cleaning and preparation of data reports. There was a suggestion that the development of centralized data infrastructures and the improvement of data use agreements are needed to streamline the decision making process and to bridge the gaps between community perspectives, public health officials, and policy makers. To better use data in decision making, some participants advocated for development of improved procedures for establishing data usage agreements that promote the collaboration among public health officials and modelers (e.g., academic experts). Other participants advocated for a stronger role of community involvement, such as training and funding that would build the capacity of local stakeholders to analyze data and create models.

Our questionnaire also asked participants to share any issues that are unique to their individual jurisdictions that must be considered when advocating for data-driven decision making. This sparked conversations regarding the anonymity of data for specific communities, a lack of data from smaller populations, and a disproportionate lack of staffing, workforce, funding and computing resources for some communities, including tribes. There was also some concern about the accuracy of data collected by outside organizations for the development of culturally sensitive interventions, where it was felt that local communities should be supported to collect and share their own experiences.

*Phase III: Opportunities and challenges for epidemiological modeling*. Phase three occurred on the second day, building on the foundations established by keynote presentations. With this new knowledge of the diverse ways in which epidemiological modeling can be deployed in practical scenarios, this breakout session focused on the opportunities and challenges for applying this type of modeling for timely public health decision support in the state of Arizona. Overall, the participants acknowledged that epidemiological modeling is a promising information source, particularly for resource allocation (e.g., deciding where and how to distribute vaccines). Although policy dependent constraints will always be an issue, predictive modeling can be an effective tool in planning resource allocation for community-specific emergency response preparedness programs and scenarios.

Certain constraints were highlighted while discussing the challenges for incorporating epidemiological modeling in the state of Arizona. Again, data issues were a large topic in this discussion; participants pointed out that a model's predictive accuracy is dependent on the availability of accurate and timely data sources flowing from the community of interest. Therefore, issues such as data scarcity, lack of updated data, lack of data sharing, trust issues on external data resources appeared again as recurrent themes. Participants also pointed out that the usefulness or accuracy of forecast models could be impeded by unique community structures or unforeseen variation in weather patterns, for instance. Additionally, it was mentioned that there is no centralized infrastructure to connect data collectors, modelers and policy/decision makers. Participants also emphasized that there is a need for zip code specific, tribe specific, and community specific data for customizing models, to allow them to perform more accurately at local scales.

An astute observation was that it is important to recognize that epidemiological models are not the ultimate answer to all of the problems faced in decision making during epidemics. Model assumptions made without carefully considering real-world, on-the-ground issues can heavily impact the accuracy of the model outputs, and thereby the utility and credibility of those outputs. Some participants felt strongly that community participation is crucial to identify the right questions that need to be answered in specific jurisdictions.

## 4 Discussion

Our forum provided education on how epidemiological modeling can be used to benefit public health, and it facilitated a broad discussion of the perceived opportunities and challenges of using predictive epidemiological modeling within the state of Arizona. Importantly, the forum offered an opportunity for public health stakeholders to highlight the priorities for their constituencies, and to voice their opinions and concerns about how data and modeling may or may not be used to influence policy within the state.

Our primary motivation for conducting this forum was to begin relationship- and trust-building between diverse stakeholders, which can grow into a collaborative process of creating modeling tools for infectious diseases in Arizona. We feel that this forum was successful in moving us closer toward productive working relationships. For several decades, qualitative research from across the globe has shown that epidemiological models are under-utilized due to various barriers, including insufficient communication and trust between modelers and key stakeholders within governments and communities (7, 28–30). Studies have therefore implored the establishment of working relationships between modelers and public health stakeholders prior to the emergence of epidemics, in both informal and formal capacities (31). The formal practices of participatory modeling and participatory epidemiology describe rigorous approaches in which modeling studies incorporate multi-disciplinary collaboration from the inception of the problem through implementation (32, 33). For instance, in Canada, there is an online community of practice that engages multi-disciplinary stakeholders from around the globe focused on modeling for public health (mod4PH), and this group has developed protocols for participatory modeling studies (34). Our research group therefore endeavors to implement such evidence-based practice of collaboration into our design and implementation of future software for modeling in Arizona.

Some of the key takeaways of our Arizona-specific forum reflect insights gleaned from previous workshops and qualitative research on epidemiological modeling in public health. There was a general agreement that data dashboards and model-based, interactive tools were approachable and that these tools improved the messaging from models. Previous surveys identified that modeling tools should produce results that are perceived as actionable by the stakeholders, but that any tool must be trusted as credible to ensure buy-in (35, 36). Therefore, in our own development of modeling tools, we endeavor to collaboratively agree upon key model outputs and routines that will address actionable concerns of diverse stakeholders. Another related takeaway was a concern about whether models are actionable when they make predictions at large spatial scales. More specifically, multiple participants would prefer model results that are customized to more local jurisdictions. This reflects the message that modeling studies could be more actionable if applied at smaller spatial scales, although understanding and forecasting infectious disease dynamics at small spatial scales suffers from unique technical challenges and challenges with data quality and quantity (37, 38).

Our forum also identified a desire for educational opportunities on modeling for the public health workforce. In East Africa, a two-week training program was created for public health practitioners to learn about the design and implementation of epidemiological models (39). While similar training programs could be more broadly and routinely offered, our participants noted that funding is not distributed equally in a way that allows for proper training or for hiring internal modeling expertise.

We heard from representatives of different jurisdictions who have different priorities and who believe that models should account for unique aspects of their jurisdictions, including aspects that impact health equity. This is especially true when considering jurisdictions that support vulnerable communities, including Tribal Nations. Indeed, a previous needs assessments within the US identified that deploying models that improve emergency preparedness of vulnerable and at-risk populations is a common concern among public health agencies (40). However, epidemiological modeling studies have not historically been focused to account for social and structural factors that impact health equity (41–43). More concerted effort is needed to preemptively build modeling frameworks that can handle these complexities and better address infectious disease forecasting from an equitable lens. Moreover, this viewpoint advocates for model customization in collaboration with local leaders and communities, and therefore reiterates the need for collaborative model development from the very beginning of or prior to an emerging problem (44, 45). We can envision opportunities and challenges for flexibly designing models that can then be adapted to local circumstances. For instance, by assembling stakeholders from many jurisdictions, consensus could be built on which model aspects would be universal across jurisdictions, while identifying specific ways a model could be minimally adapted to accommodate local idiosyncrasies.

We believe that this forum was an important first step in forging new understanding and partnerships between modeling experts and public health experts and advocates within Arizona. Earnestly responding to the lessons learned within this forum will be critical to sustain such partnerships in the face of ongoing and emerging epidemiological threats. Already, this forum has led to grant-writing partnerships between State agencies and academic partners in the spirit of more rigorously implementing epidemiological modeling for public health. Our plan is to make this forum a semi-annual event, to continue to build connections, trust, and collaborative partnerships between modelers, public health officials, political representatives, and ultimately, with citizens. Accordingly, we plan to add participants from additional diverse backgrounds and provide more hands-on learning and assessment of the use of models in real-world scenarios, e.g., realistic scenario-based exercises where participants use modeling tools and simulated data streams to shape a response to an epidemic. We hope these multidisciplinary learning and engagement opportunities continue to inspire innovative ways to incorporate modeling into public health practice. Indeed, previous stakeholder engagement has emphasized the desire for better modeling tools for operational use (29, 35, 36). We therefore plan to continually learn from public health partners in focused ways that will guide our building and testing of modeling software, such that we can ensure our software meets the needs of an evolving public health landscape.

## Data availability statement

The original contributions presented in the study are included in the article/Supplementary material, further inquiries can be directed to the corresponding author.

## Ethics statement

Ethical review and approval was not required for this study in accordance with the local legislation and institutional requirements. Written informed consent from the program evaluation focus group participants was not required to participate in this study in accordance with the national legislation and the institutional requirements.

## Author contributions

JM: Conceptualization, Funding acquisition, Methodology, Project administration, Supervision, Writing – original draft, Writing – review & editing. CC: Methodology, Writing – original draft, Writing – review & editing. MM: Methodology, Writing – original draft, Writing – review & editing. KO: Methodology, Writing – original draft, Writing – review & editing. ED: Conceptualization, Funding acquisition, Writing – review & editing. EG: Conceptualization, Funding acquisition, Writing – review & editing. CH: Conceptualization, Funding acquisition, Writing – review & editing. TL: Conceptualization, Funding acquisition, Writing – review & editing. SM: Conceptualization, Funding acquisition, Writing – review & editing. SS: Conceptualization, Funding acquisition, Writing – review & editing.
