# Supplementary material for: An inaugural forum on epidemiological modeling for public health stakeholders in Arizona
Source: Front Public Health. 2024 May 31;12:1357908. doi: 10.3389/fpubh.2024.1357908 (PMC11176426; doi:10.3389/fpubh.2024.1357908)
Supplement: Supplementary file 1 [file Presentation_1.PDF]

NAU NORTHERN ARIZONA UNIVERSITY

School of Informatics, Computing, and Cyber Systems

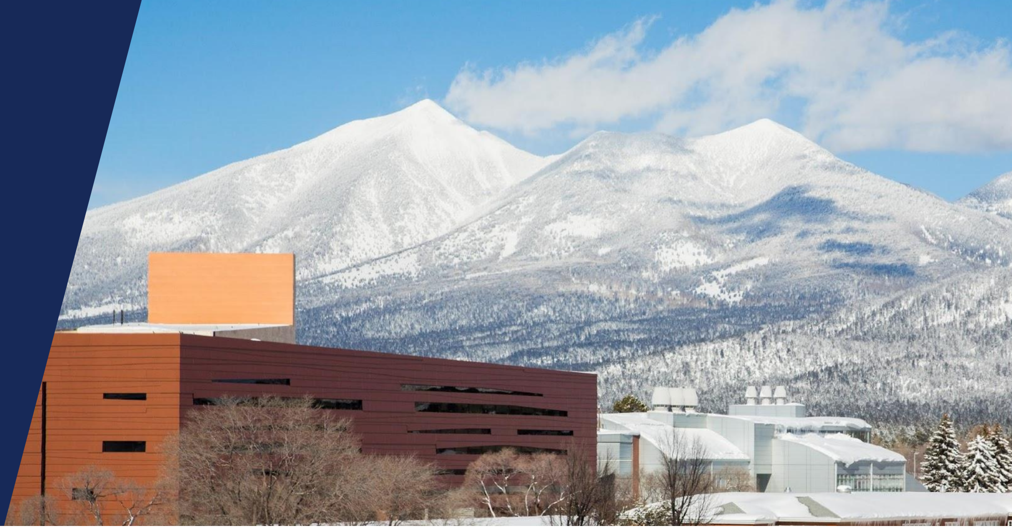

PREDICTIVE MODELING FOR PUBLIC HEALTH

An inaugural forum on data-driven technologies to support public health planning in Arizona

MARCH 8 – 10, 2023

Northern Arizona University Campus, Flagstaff, AZ

We seek to assemble and learn from a coalition of multidisciplinary professionals to envision a future of local public health planning for **infectious disease**, facilitated by **predictive modeling technologies** that contribute to planning, communication, and decision-making.

For this inaugural forum, we are bringing together public health stakeholders from across Arizona, innovative speakers, and academics to:

- **Provide education** on *epidemiological modeling* and other data-driven technologies for infectious diseases,
- **Describe our plans** for building a next-generation decision-support tool for infectious disease epidemics, and
- **Forge partnerships** between modelers and public health stakeholders here in Arizona and beyond.

EpiMoRPH
An Automated Epi Modeling Framework

Funded by:

NIH National Institute of Allergy and Infectious Diseases

Hosted by:
Joseph Mihaljevic, PhD
Assistant Professor
