# Supplementary material for: An inaugural forum on epidemiological modeling for public health stakeholders in Arizona
Source: Front Public Health. 2024 May 31;12:1357908. doi: 10.3389/fpubh.2024.1357908 (PMC11176426; doi:10.3389/fpubh.2024.1357908)
Supplement: Supplementary file 2 [file Presentation_2.pdf]

Predictive Modeling for Public Health

WELCOME

March 8 – 10, 2023

NAU
NORTHERN ARIZONA UNIVERSITY

On behalf of Northern Arizona University, we would like to express our gratitude and appreciation to the sacred land on which this educational institution resides. This sacred land is enriched with indigenous history and culture that lives on to this day. Currently, the Zuni, Apache, Yavapai, Hualapai, Havasupai, Paiute, Diné, Hopi and many other Arizona tribal nations recognize this land as a significant spiritual place. We, here at NAU, are very fortunate to live, work, and share this unique location.

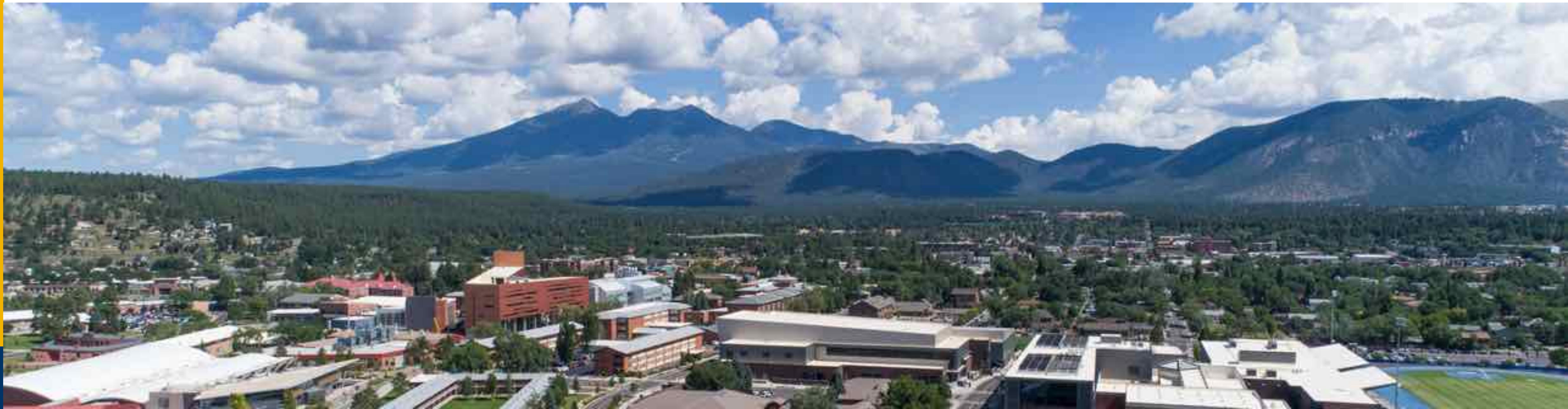

THANK YOU, ATTENDEES!

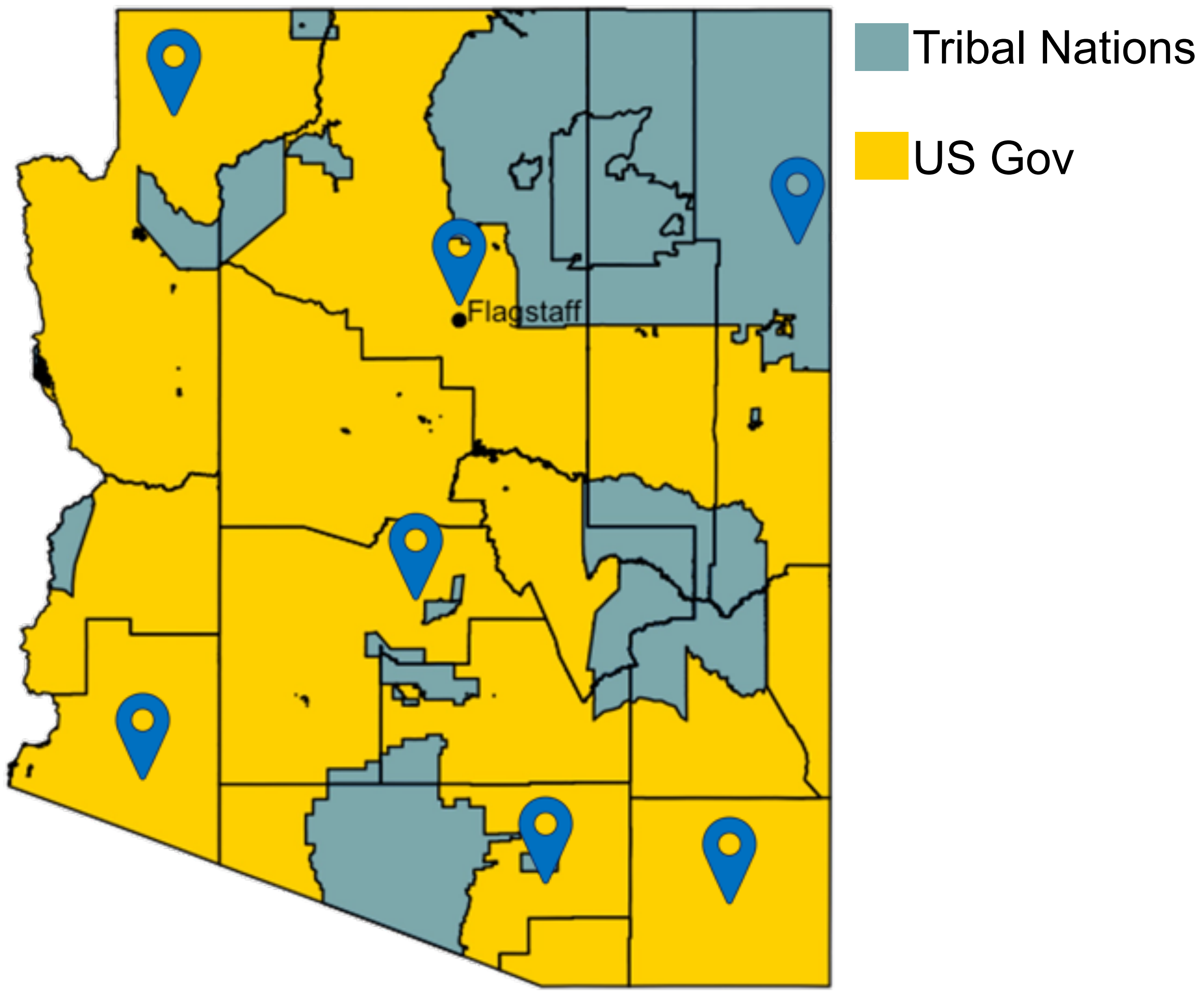

NAU
NORTHERN ARIZONA UNIVERSITY

THANK YOU, ATTENDEES!

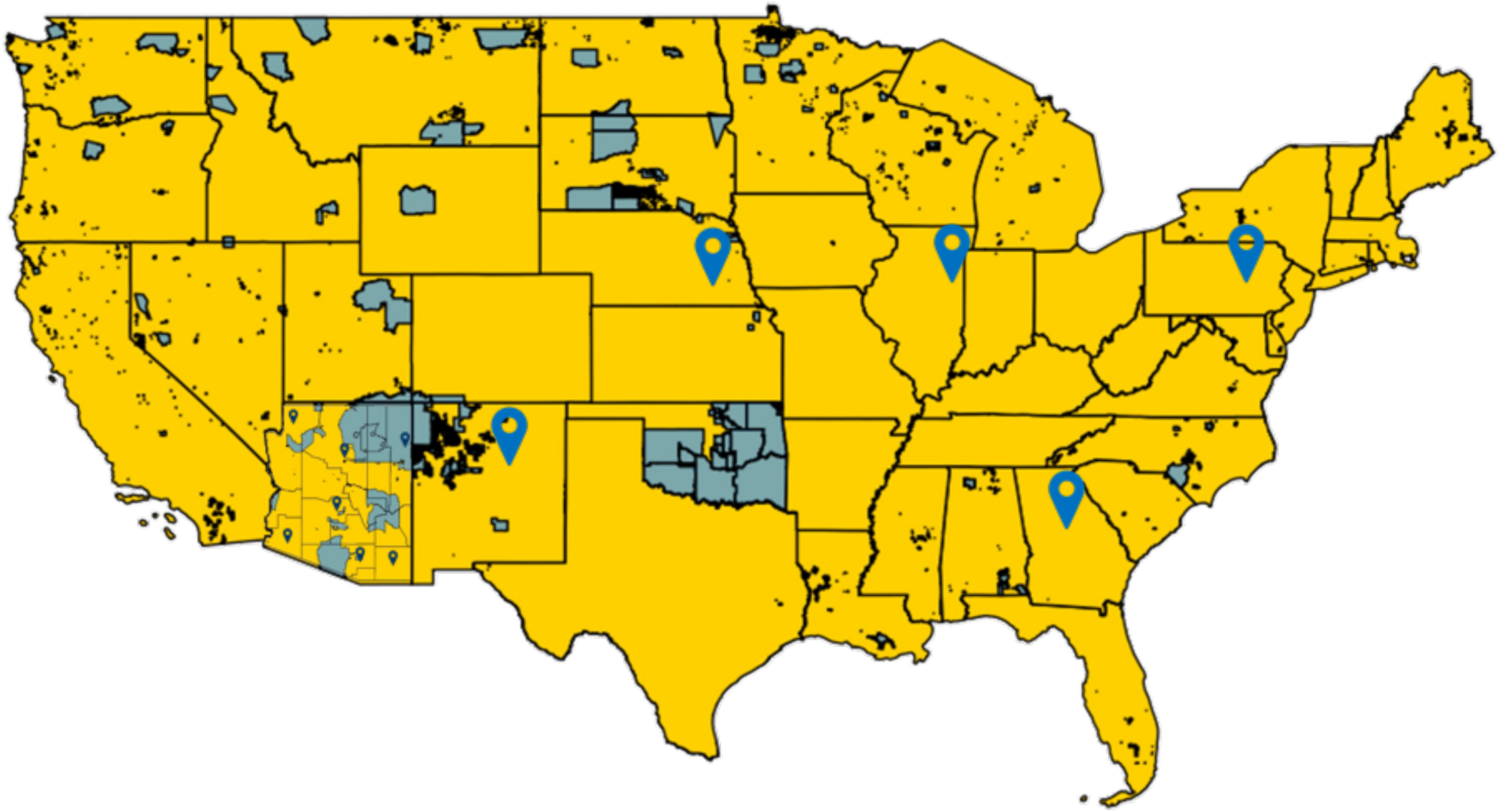

THANK YOU, ATTENDEES!

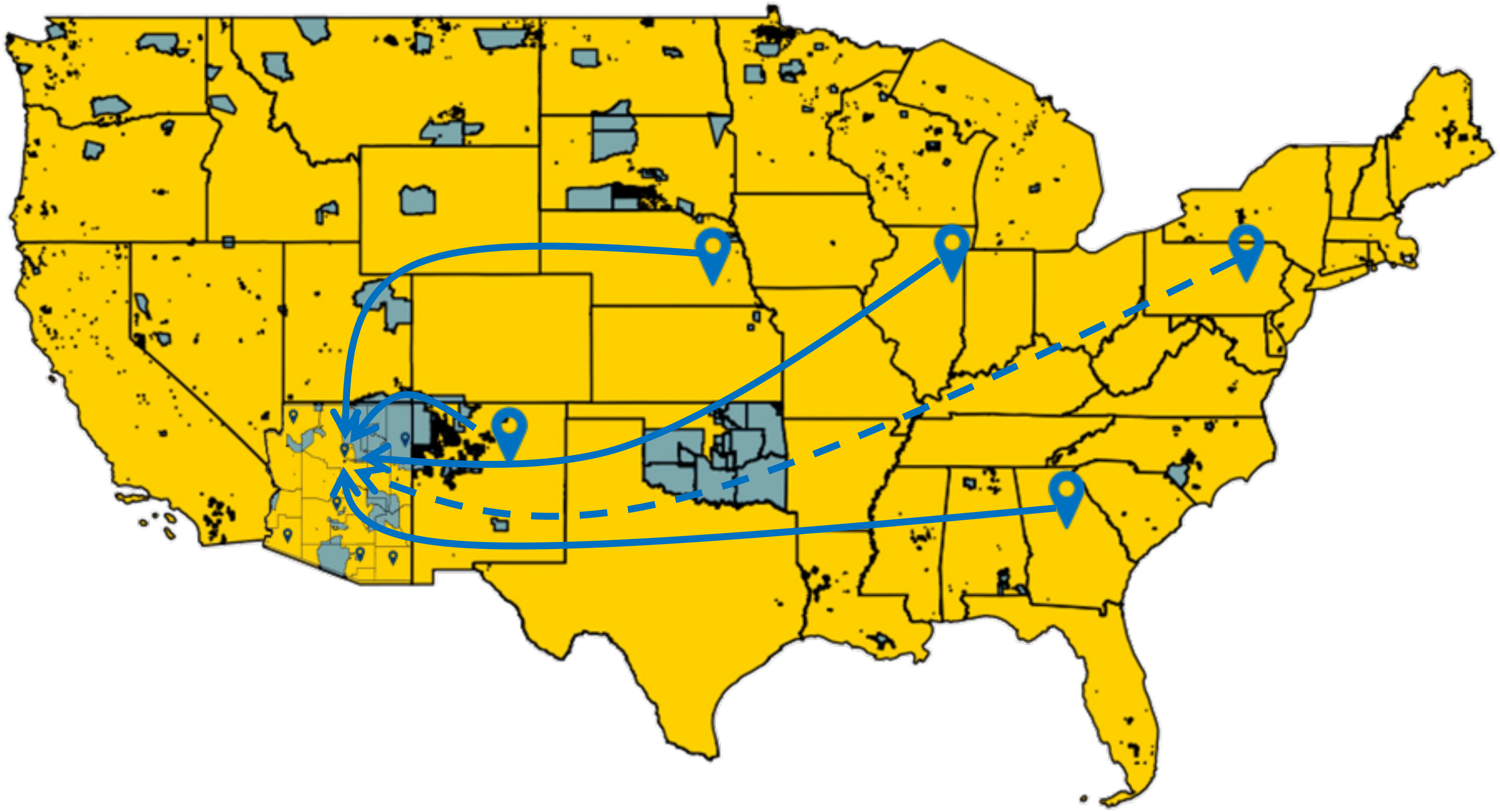

THANK YOU!

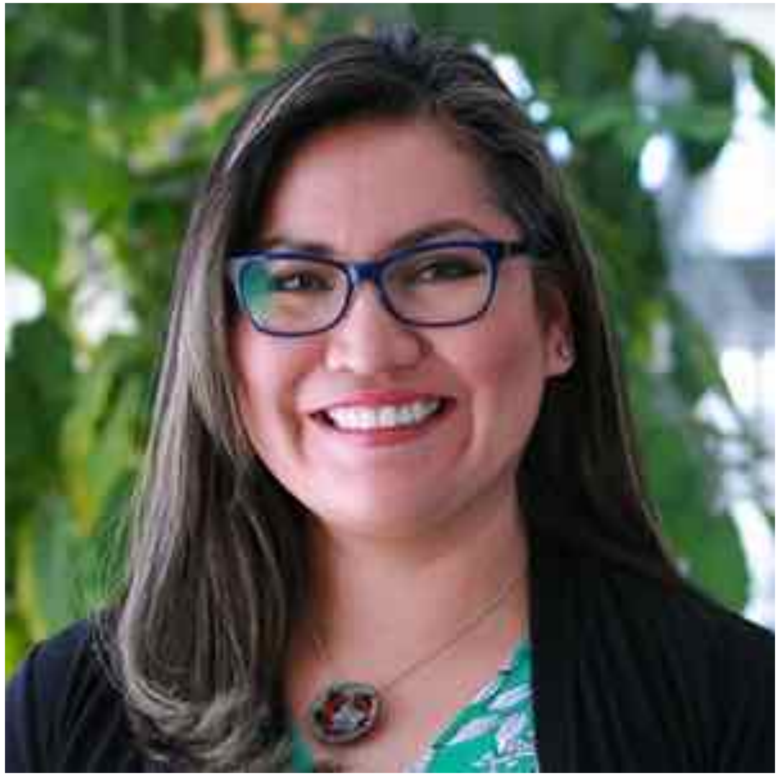

Carmenlita Chief, MPH
Program Coordinator, Sr

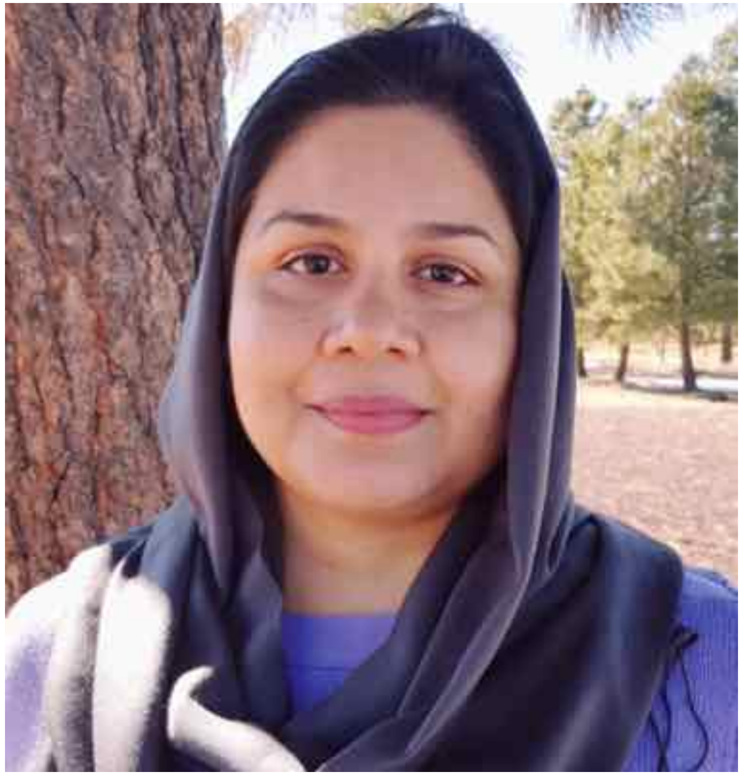

Mehreen Malik, MPH
PhD Student

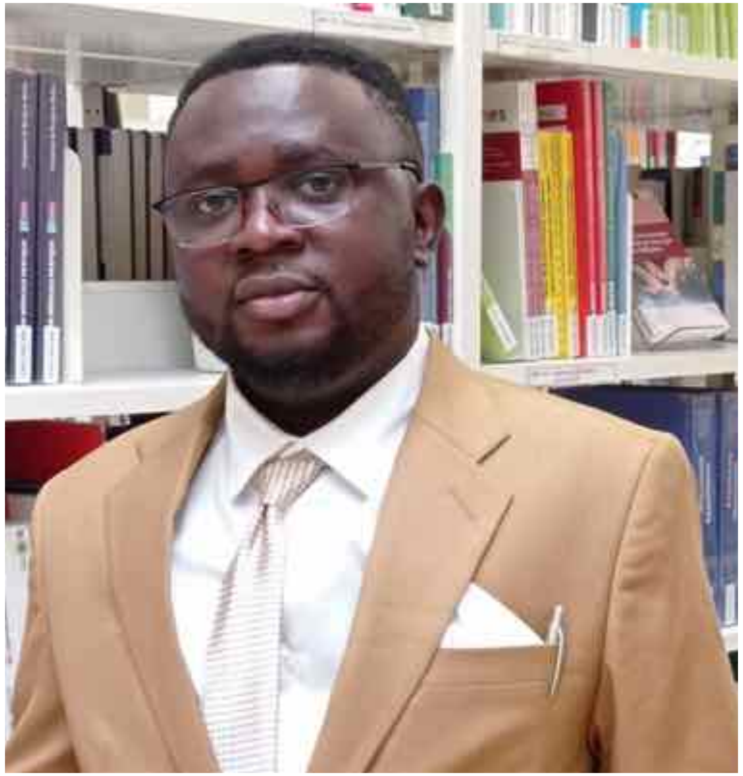

Kayode Oshinubi, PhD
Postdoctoral Scholar

EpiMoRPH

An Automated Epi Modeling Framework

NIH National Institute of Allergy and Infectious Diseases

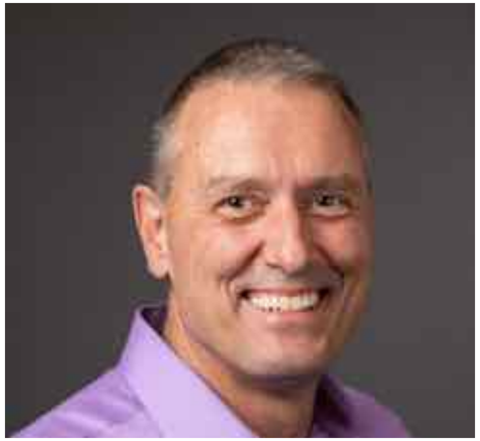

Eck Doerry, PhD
Professor, NAU

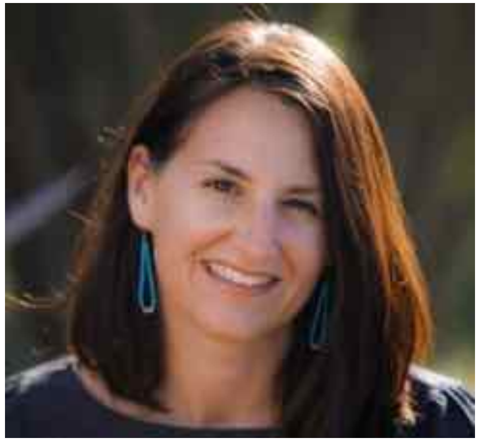

Sam Sabo, PhD
Associate Professor, NAU

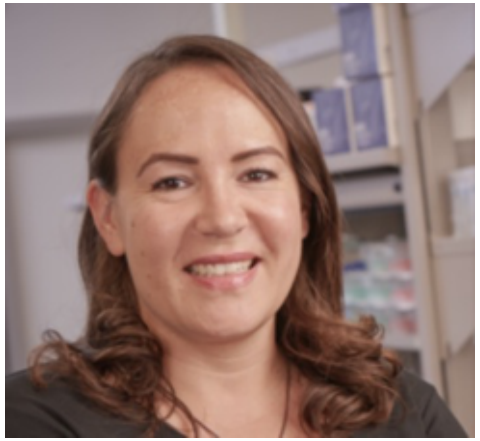

Crystal Hepp, PhD
Associate Professor, TGen

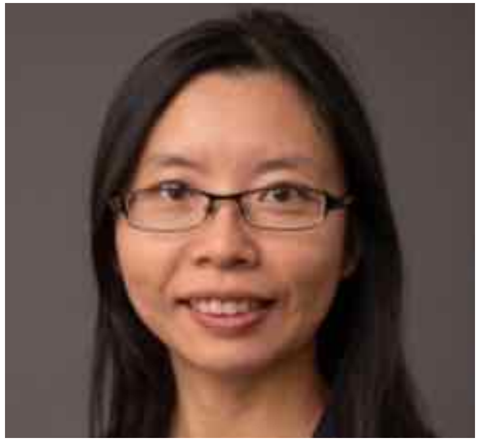

Ye Chen, PhD
Assistant Professor, NAU

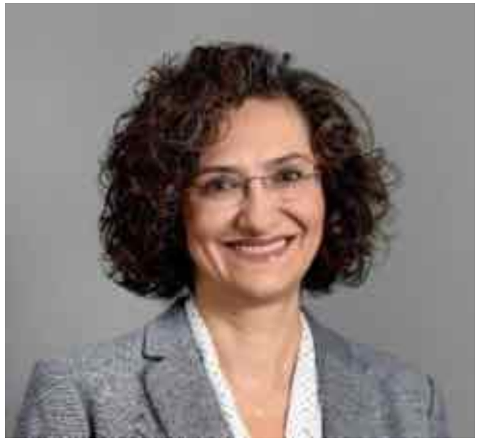

Esma Gel, PhD
Professor, UNL

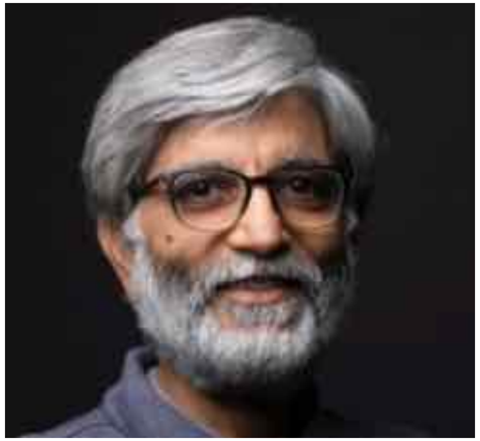

Sanjay Mehrotra, PhD
Professor, Northwestern

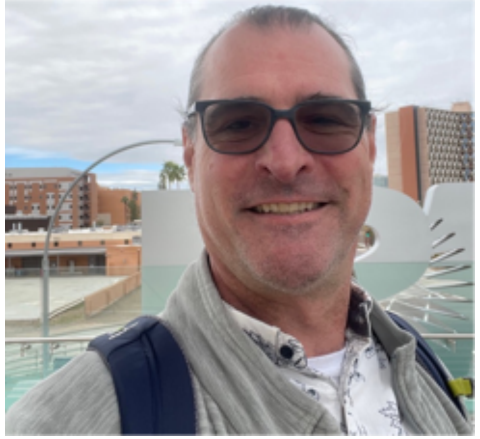

Tim Lant, PhD
Director, Research Dev, ASU

THANK YOU, KEYNOTES!

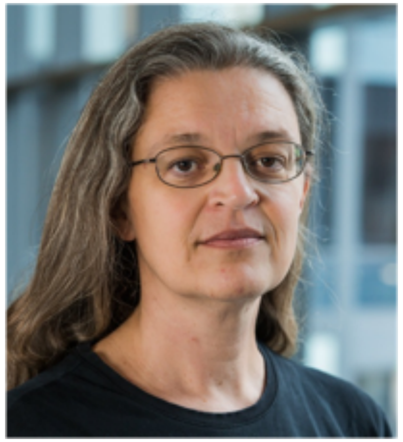

KATRIONA SHEA
Pennsylvania State University

Cooperation and coordination of multiple models to manage the COVID-19 pandemic

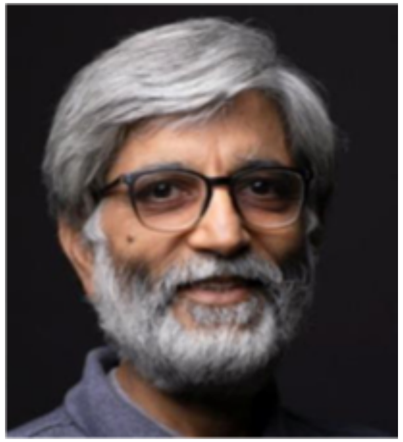

SANJAY MEHROTRA
Northwestern University

Optimizing the Allocation of Ventilators for COVID-19

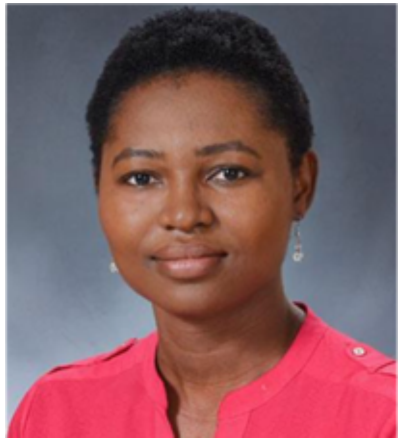

IFEOMA OZODIEGWU
Northwestern University

Participatory Design of Malaria Modeling Frameworks with Local Stakeholders: Experiences from Nigeria

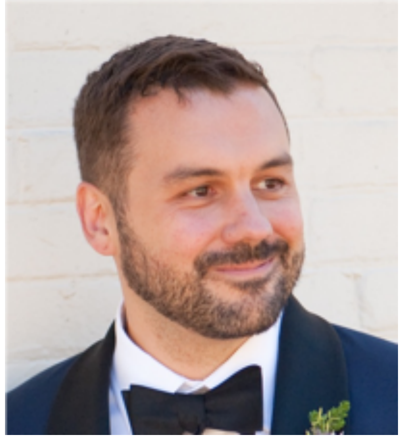

ETHAN ROMERO-SEVERSON
Los Alamos National Lab

Predictive Modeling for Sexually-Transmitted and Vector-Borne Diseases

With a special presentation by:

DANIELLE RICHARD
EMILY ANN MEYER
Centers for Disease Control and Prevention

PROVIDE EDUCATION

- *Epidemiological modeling*
- *Multi-model predictions*
- *Resource allocation*
- *Stakeholder participation*

PROVIDE EDUCATION

FORGE PARTNERSHIPS

- *Breakout discussions*
- *Networking*

PROVIDE EDUCATION

FORGE PARTNERSHIPS

DEVELOP PLANS

PROVIDE
EDUCATION

EPIDEMIOLOGICAL MODELING 101

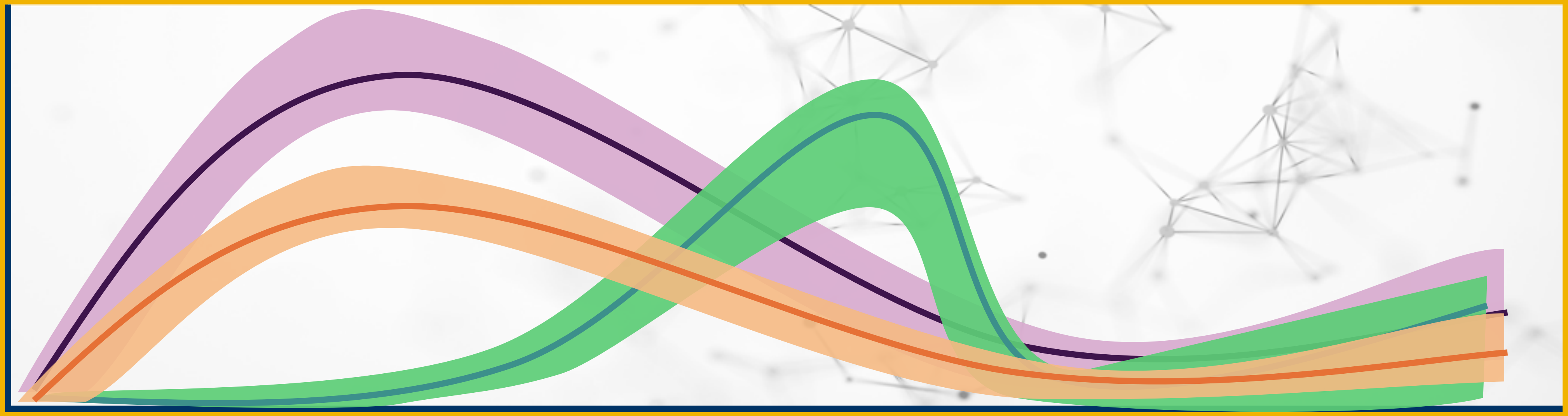

What is an epidemiological model?

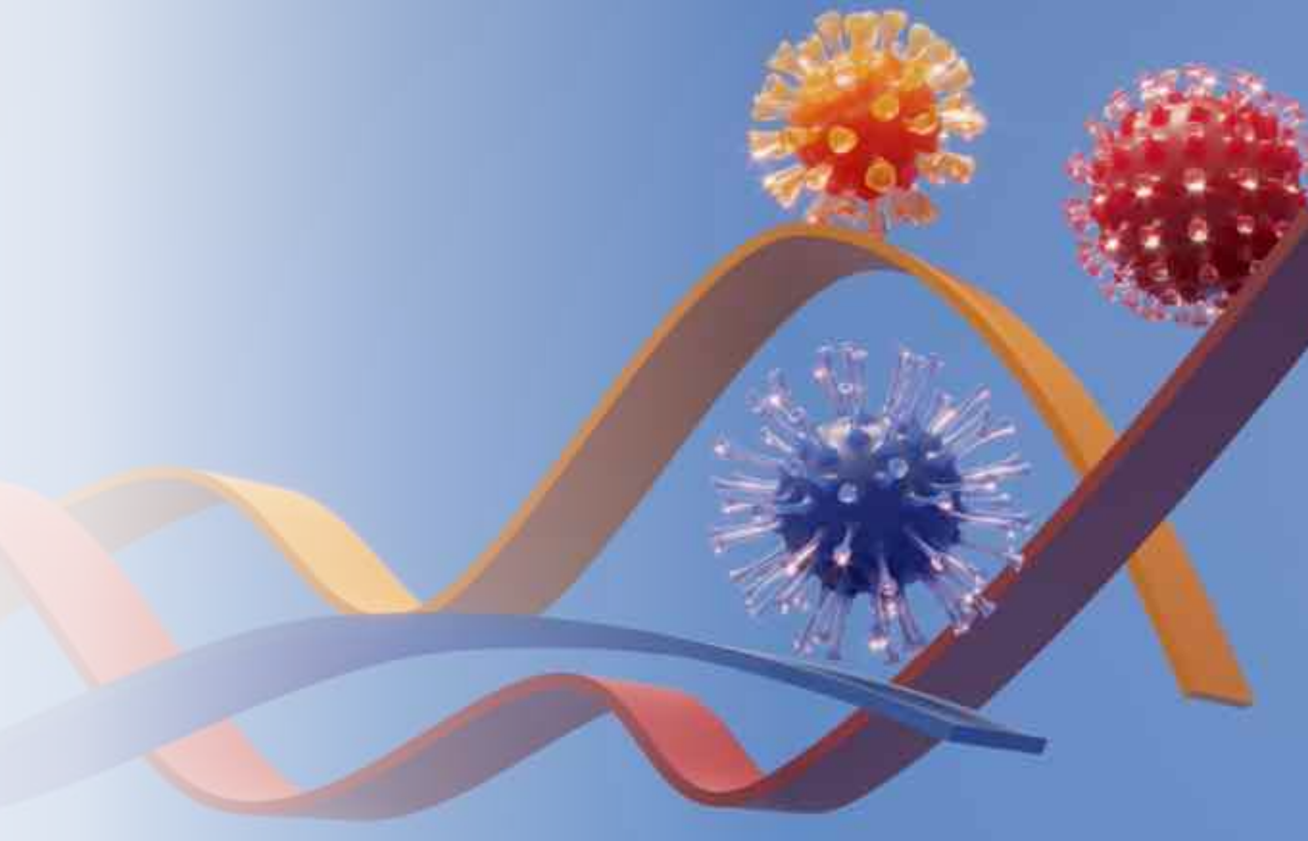

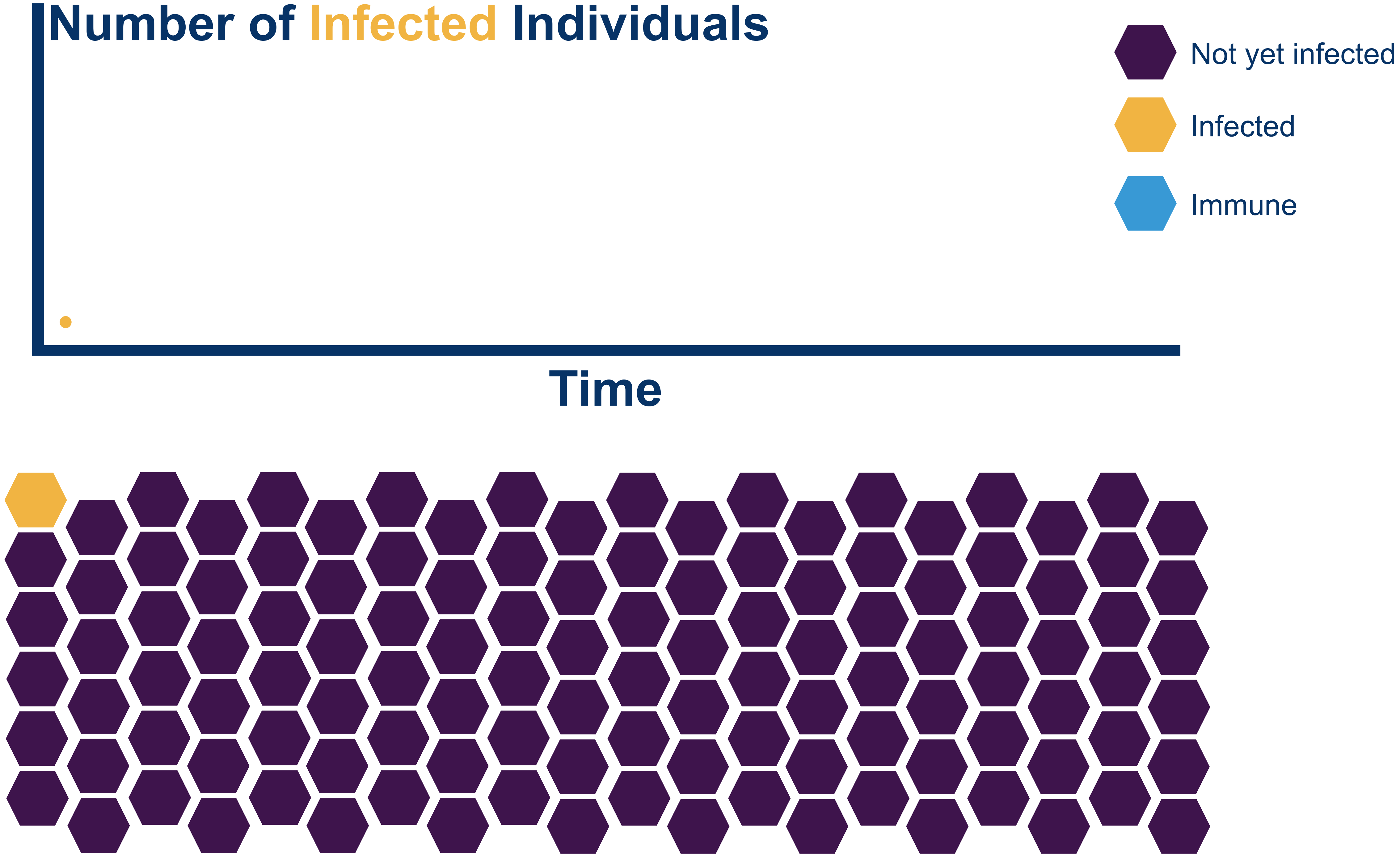

Number of Infected Individuals
Not yet infected
Infected
Immune
Time

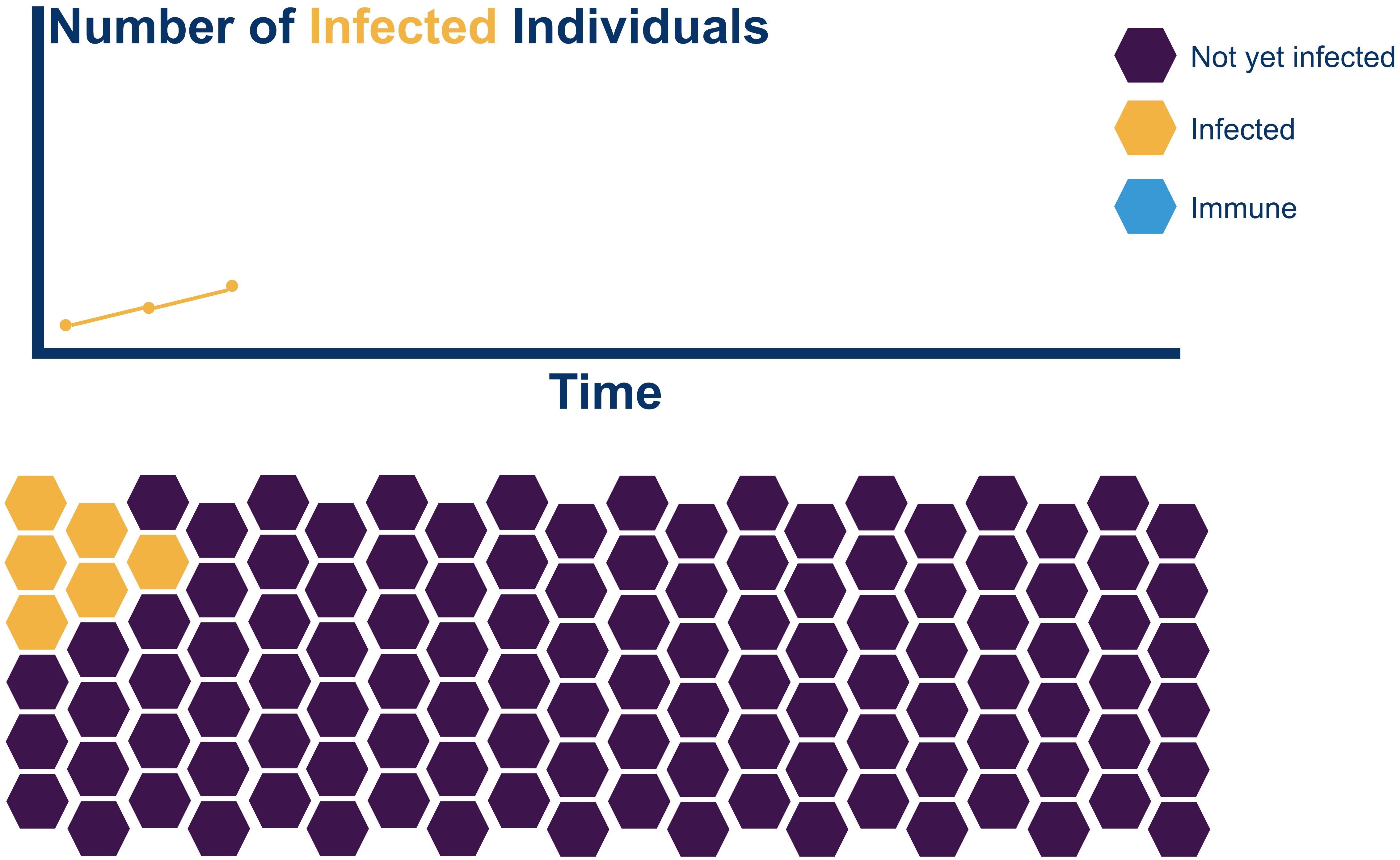
Number of Infected Individuals
Not yet infected
Infected
Immune
Time

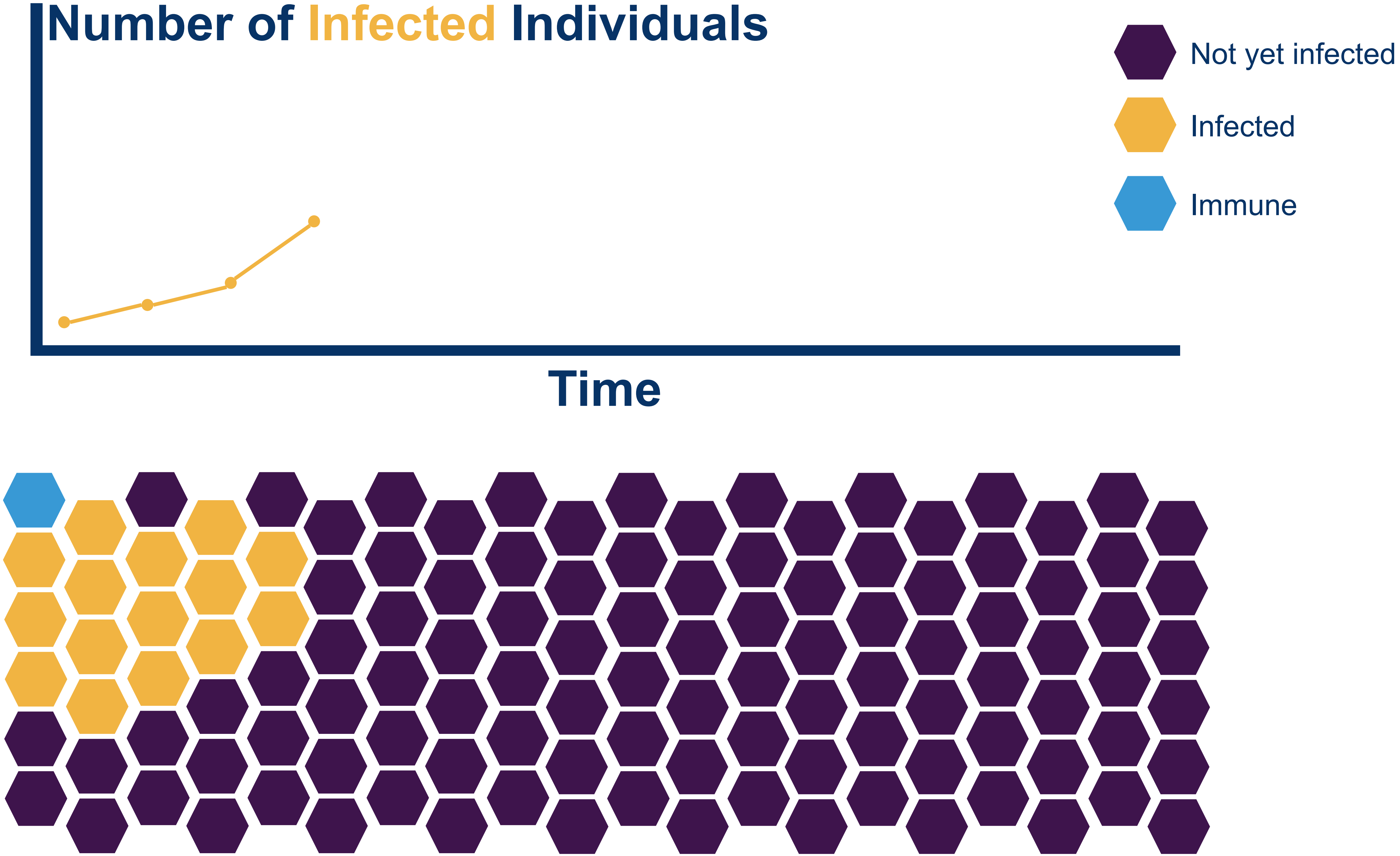

Number of Infected Individuals
Time
Not yet infected
Infected
Immune

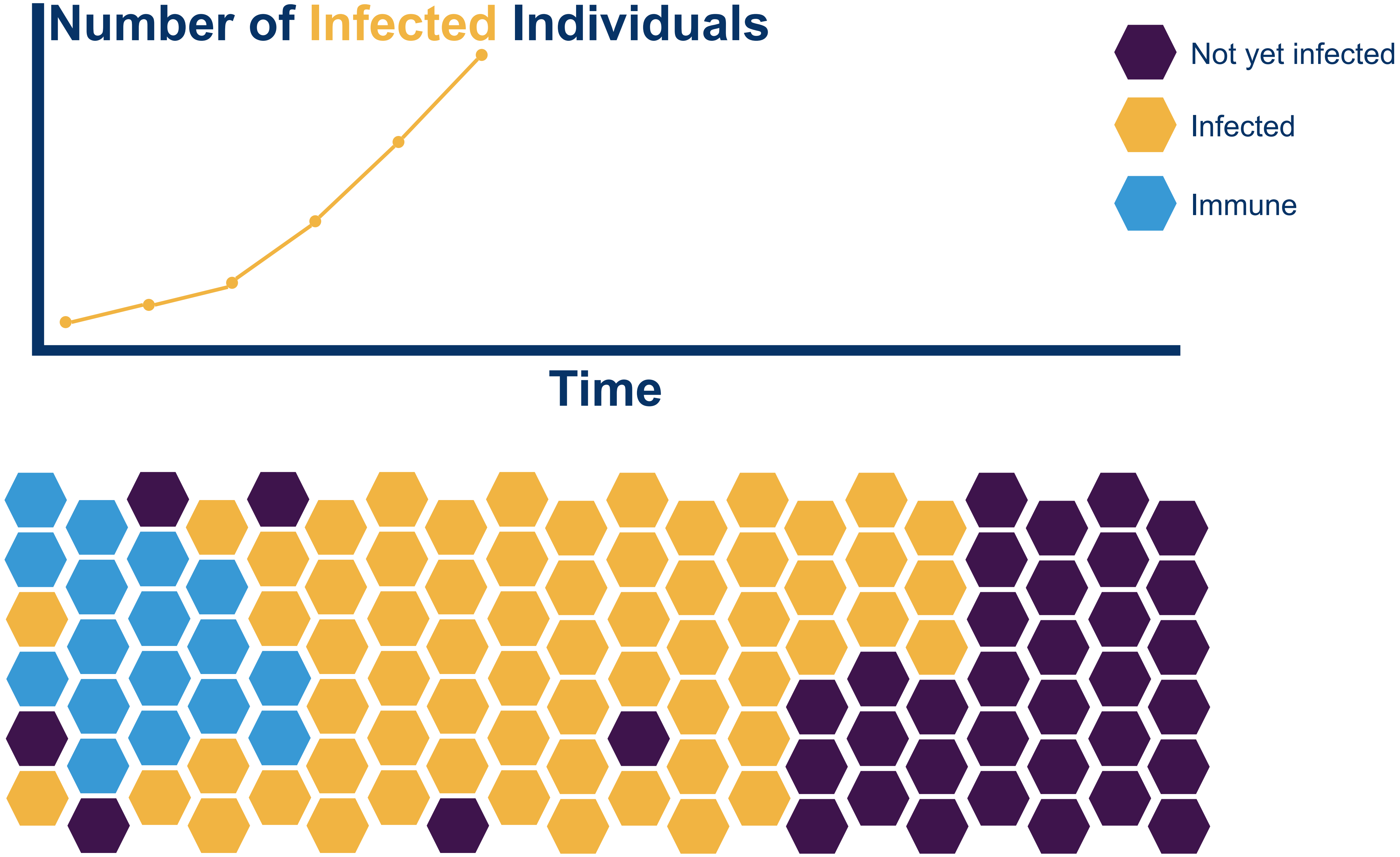

Number of Infected Individuals
Not yet infected
Infected
Immune
Time

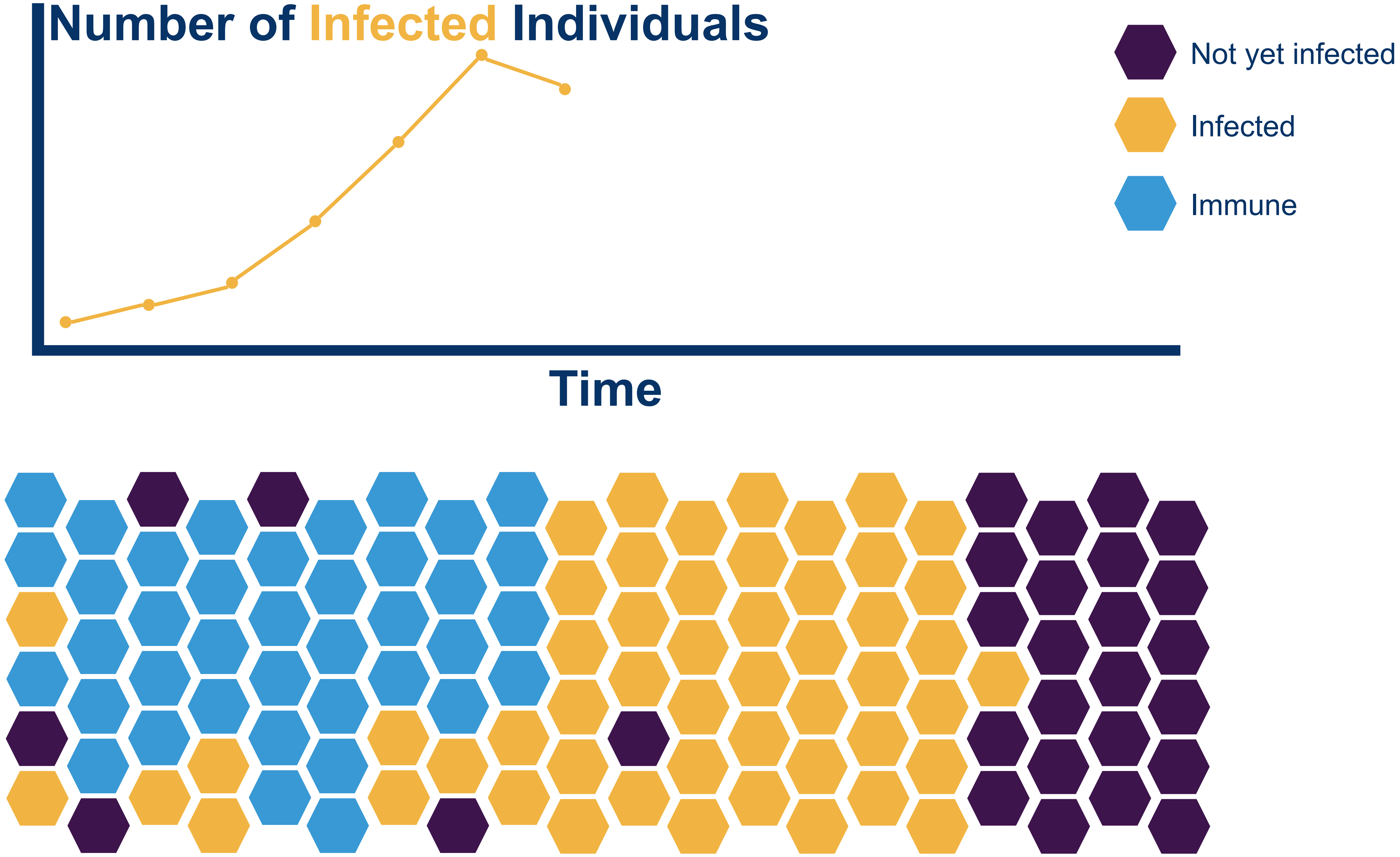
Number of Infected Individuals
Not yet infected
Infected
Immune
Time

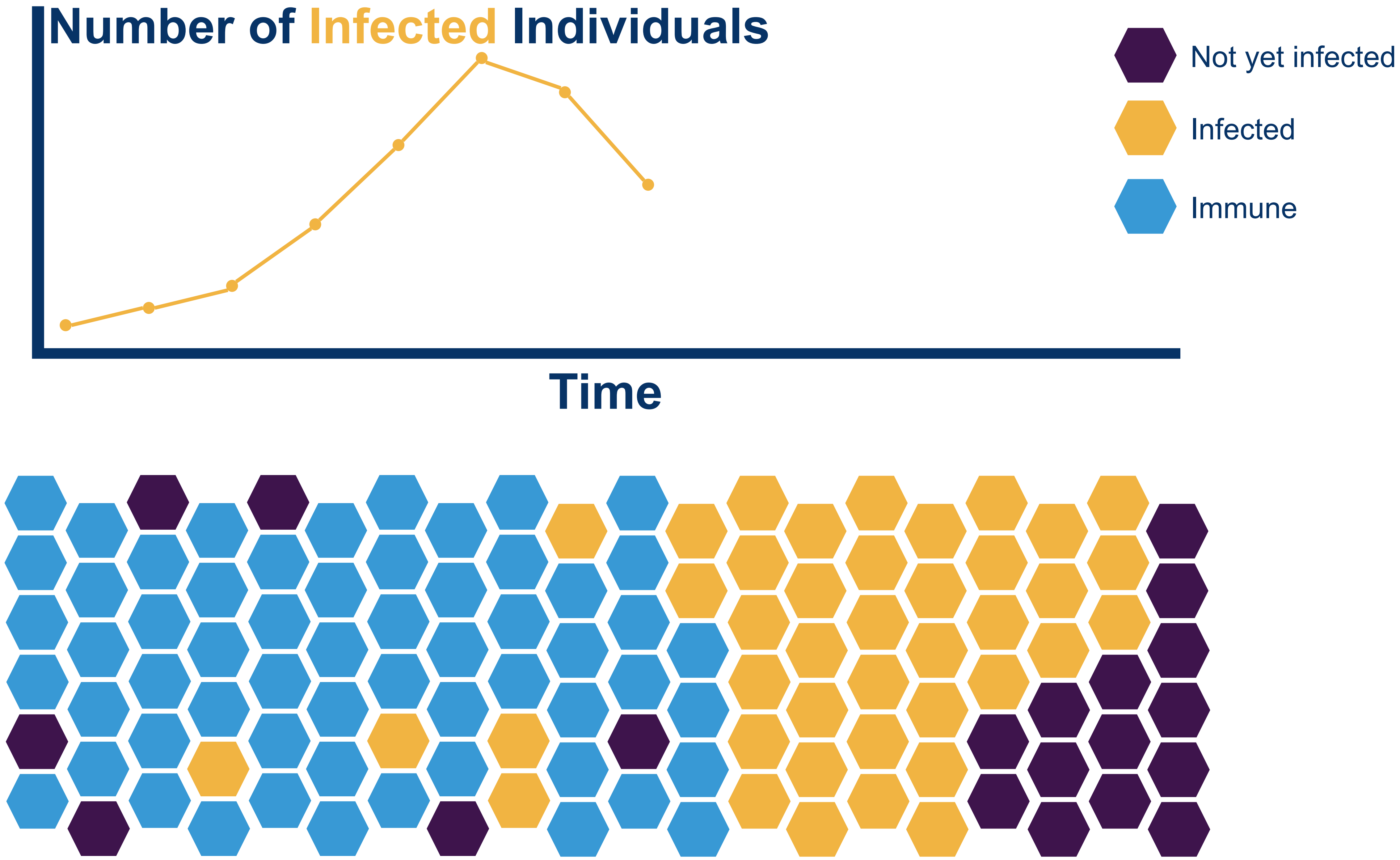
Number of Infected Individuals
Not yet infected
Infected
Immune
Time

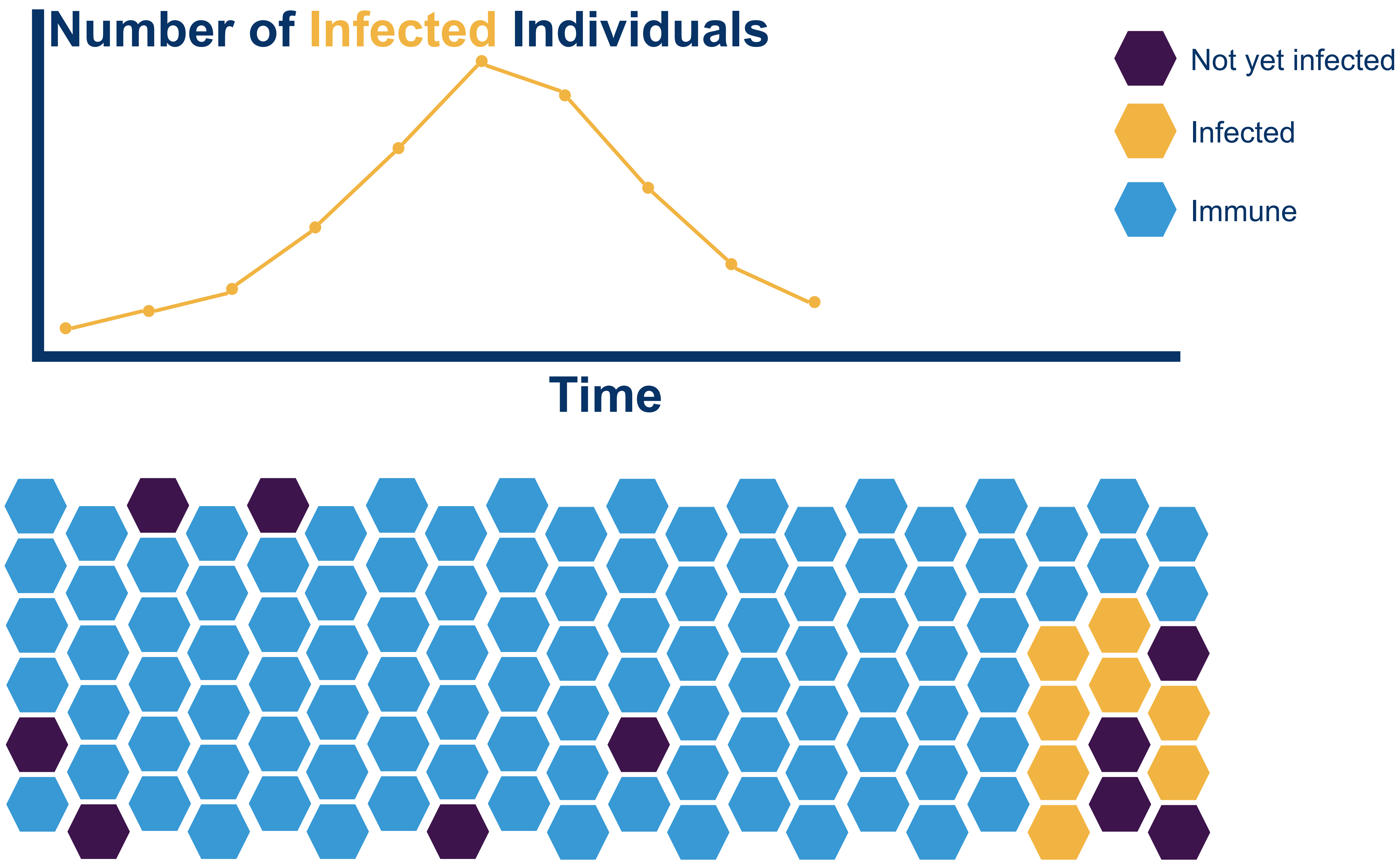
Number of Infected Individuals
Not yet infected
Infected
Immune
Time

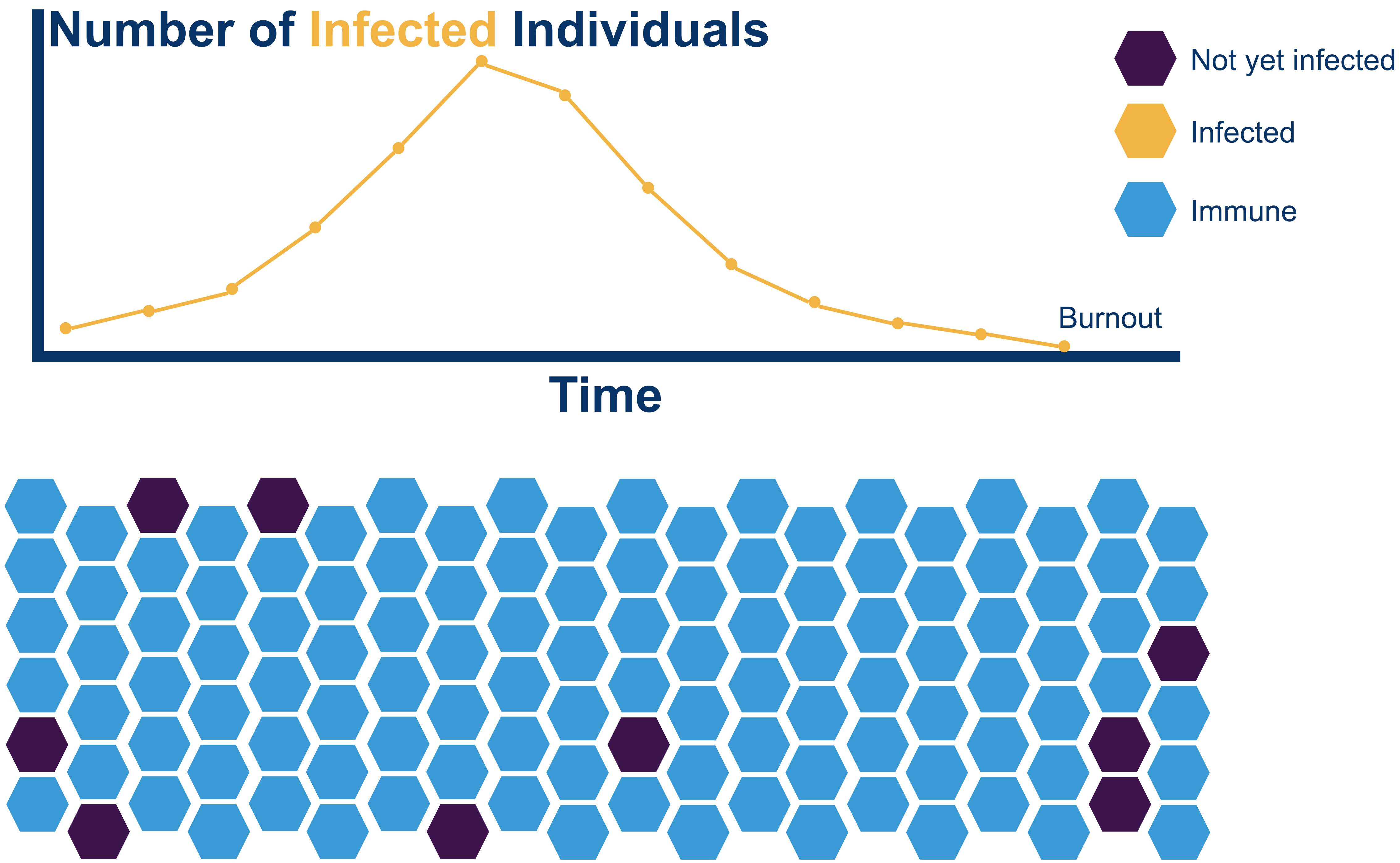
Number of Infected Individuals
Not yet infected
Infected
Immune
Burnout
Time

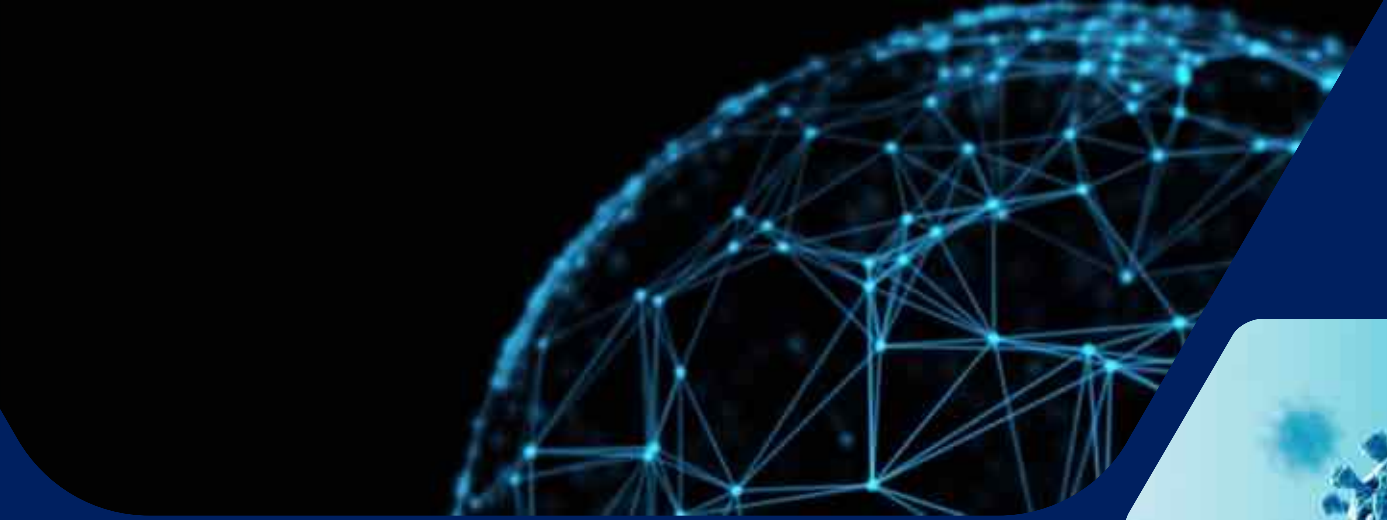

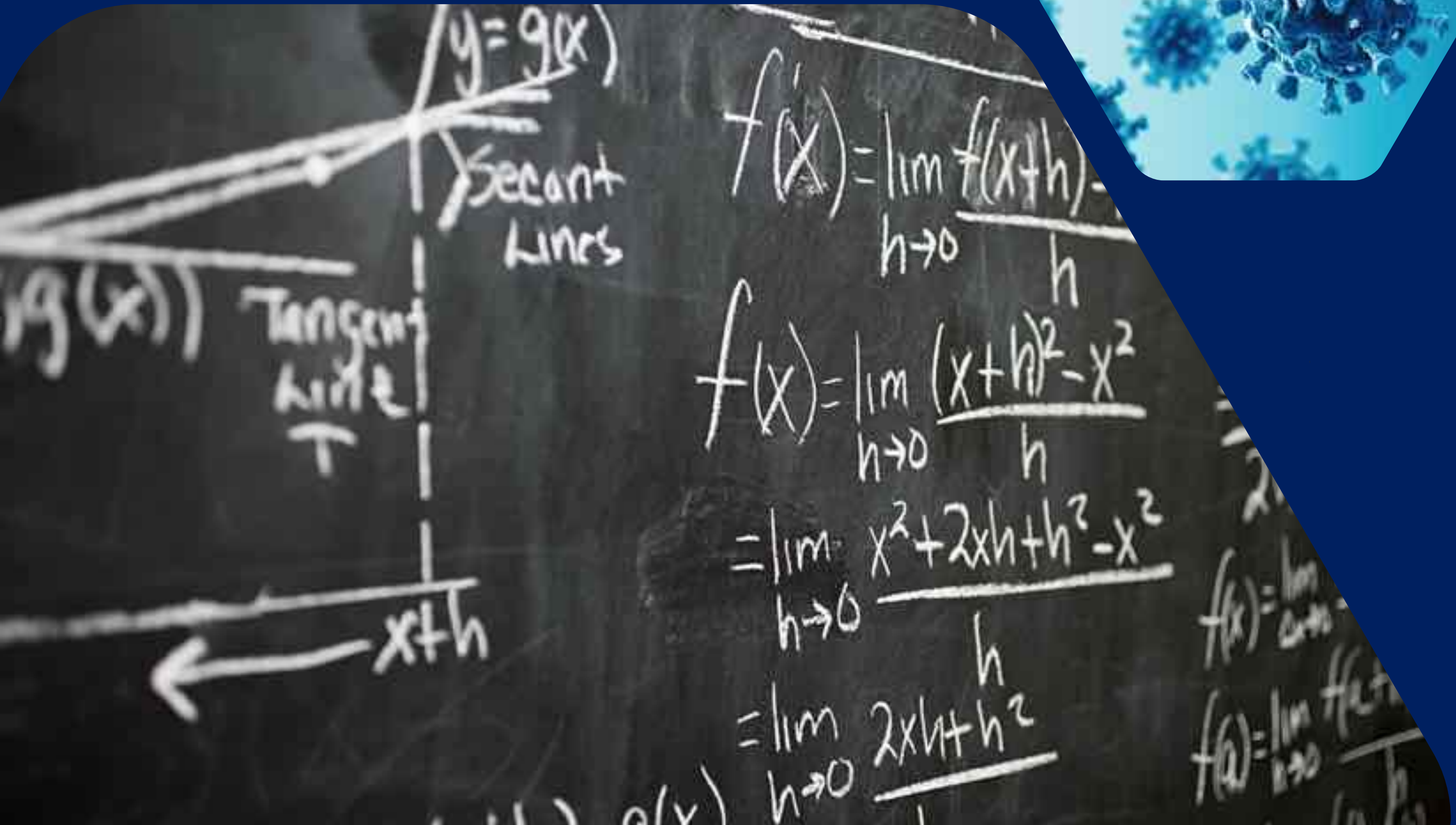

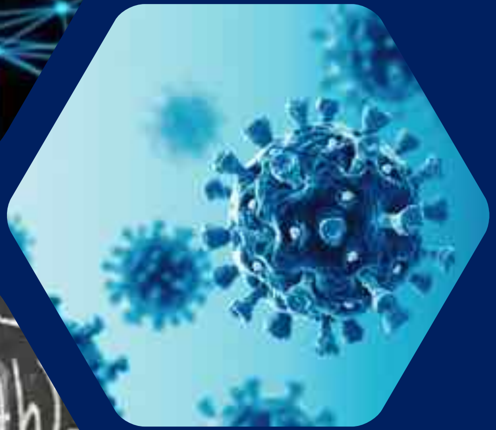

EPI MODEL:

- A representation of how we believe a pathogen transmits within and among populations

- Formalized with mathematics

How is a model created?

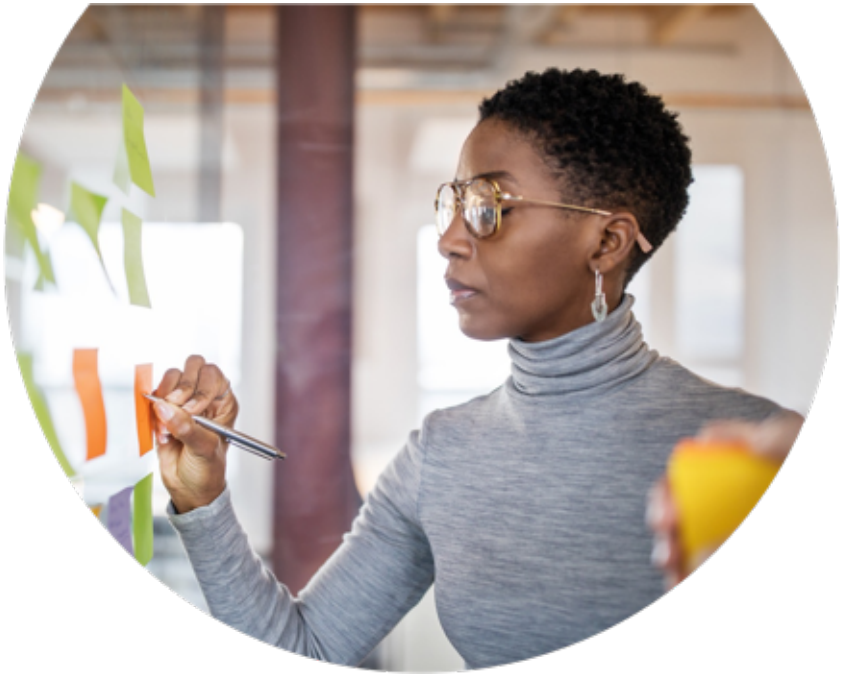

Ideas & Assumptions

- How does the pathogen transmit?
- What factors influence spread & disease?

How is a model created?

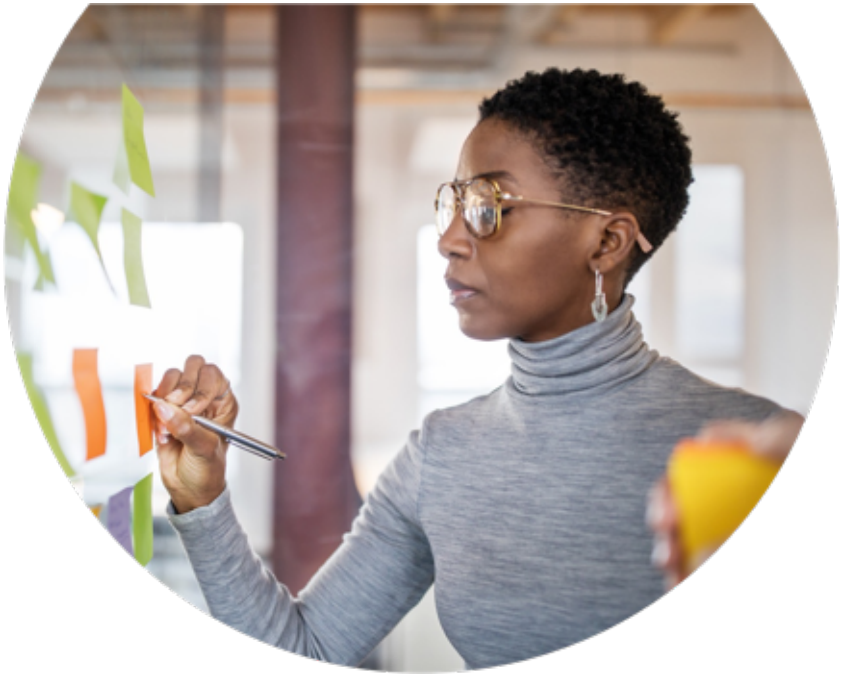

Ideas & Assumptions

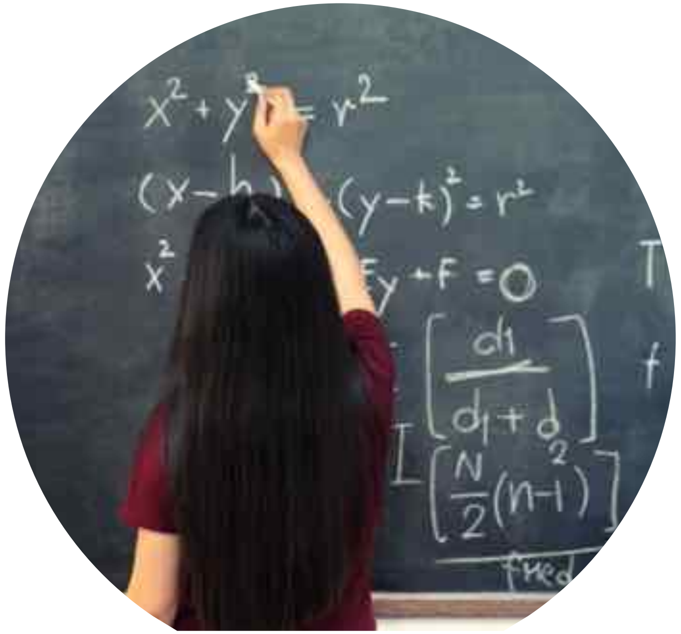

Mathematical Equations

How is a model created?

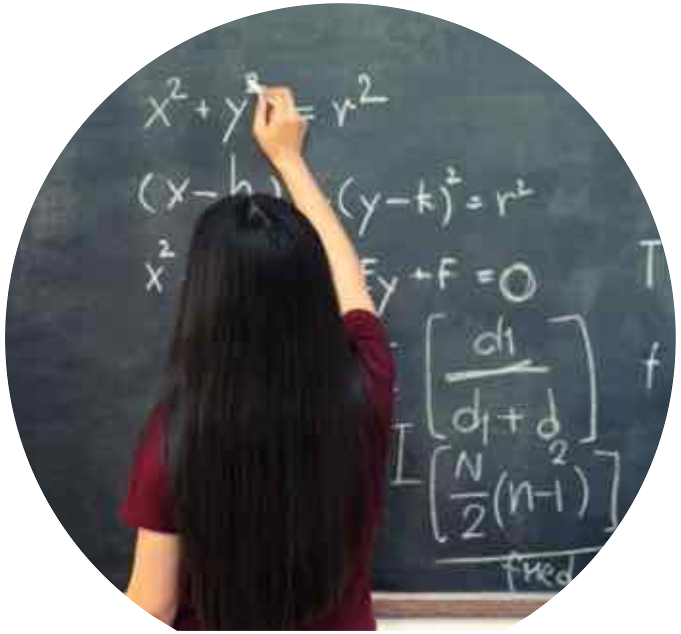

Mathematical Equations

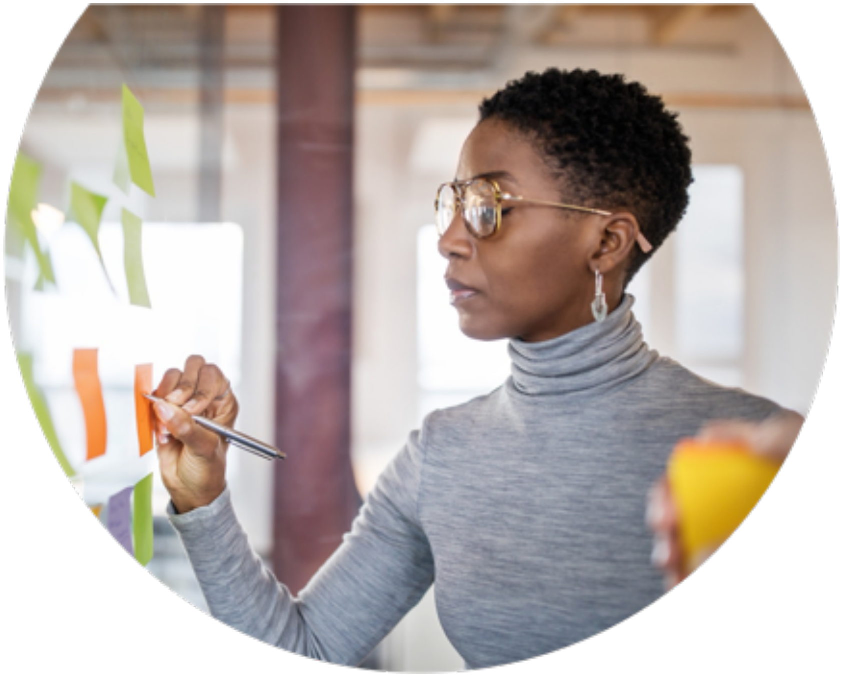

Ideas & Assumptions

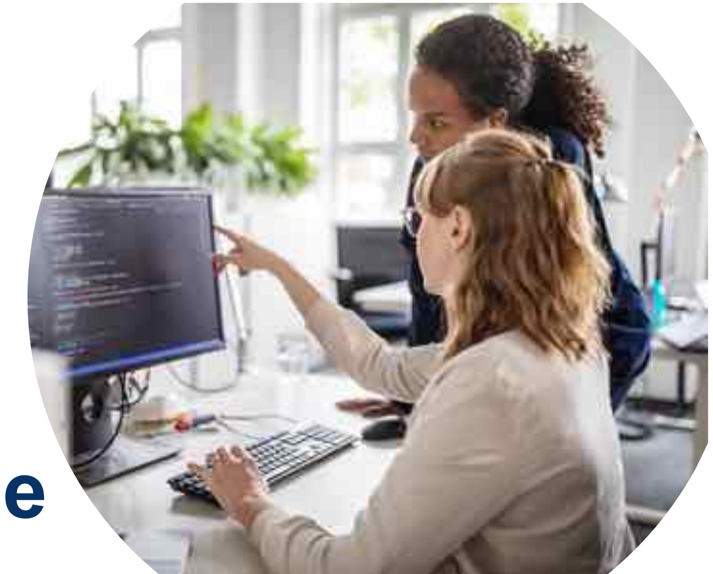

Computer Code

How is a model created?

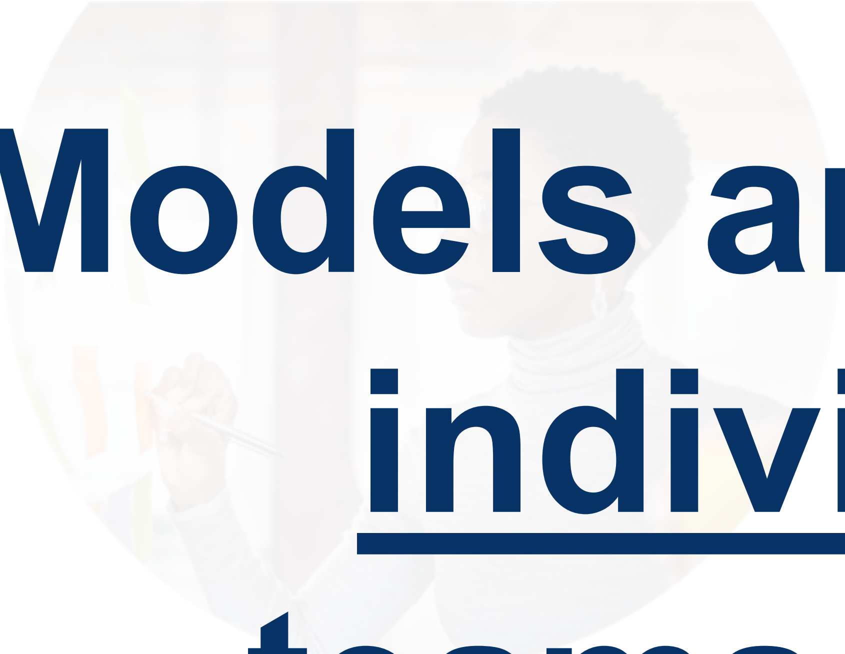

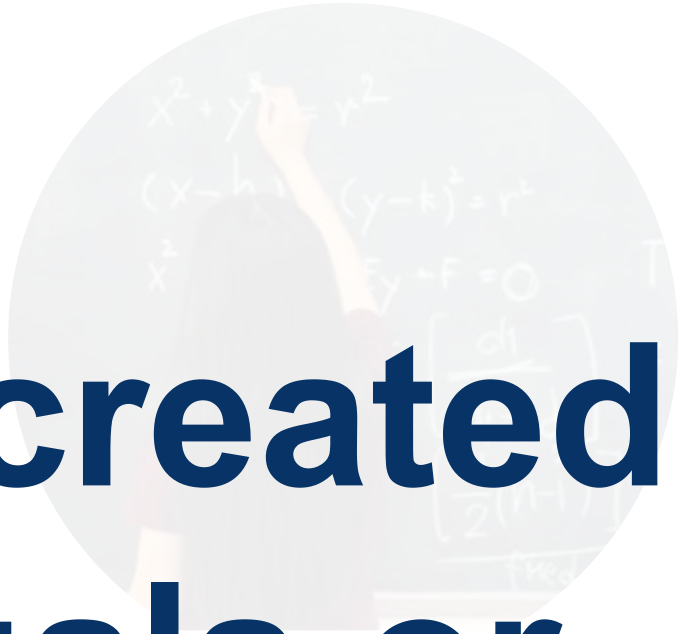

Models are created by <u>individuals</u> or <u>teams</u> of people

[illegible] Equations

Idea[illegible]

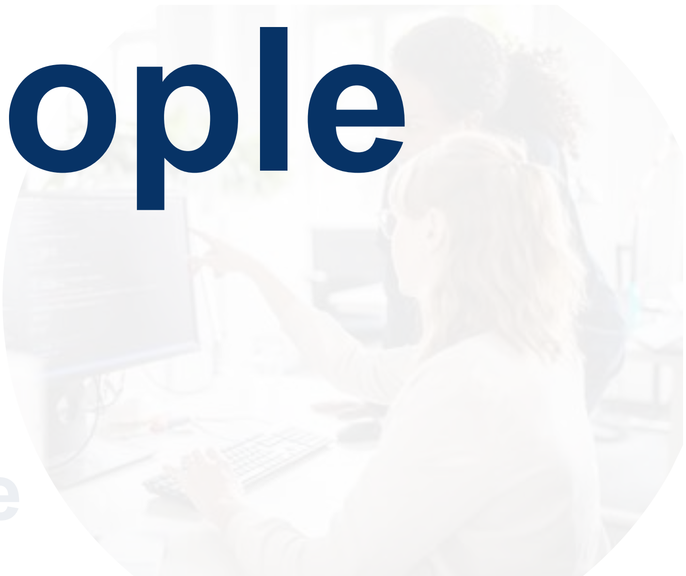

Computer Code

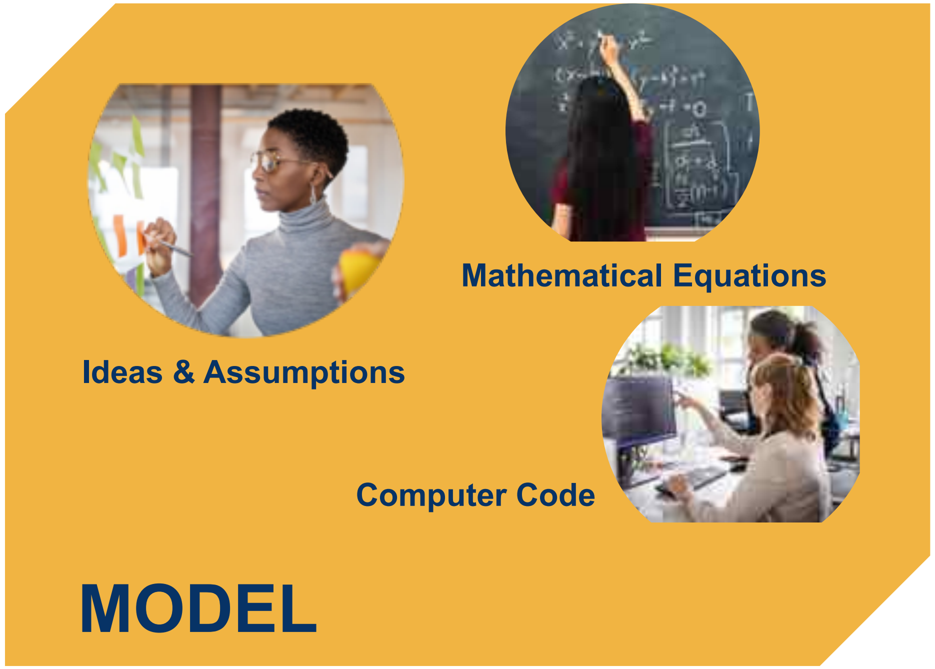

Now we can simulate the model

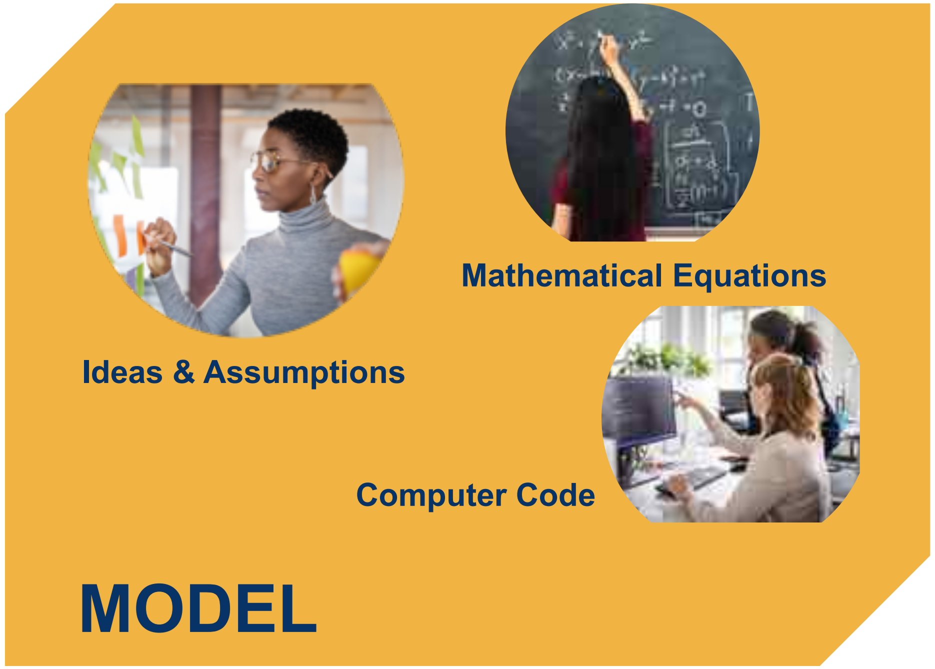

Now we can simulate the model

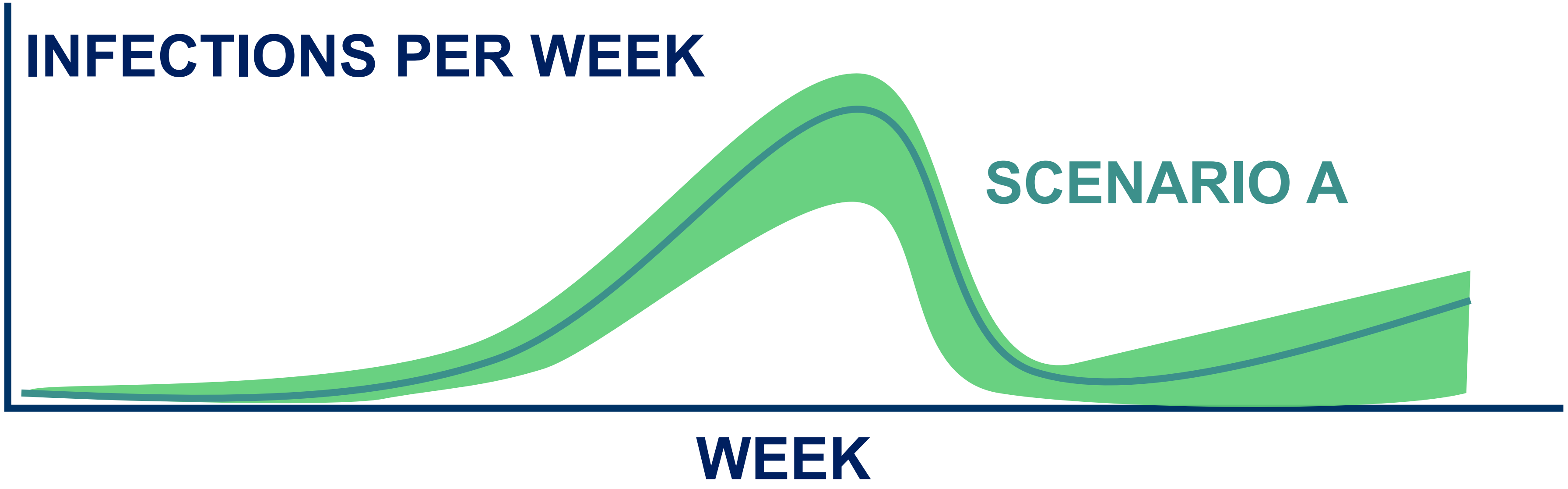

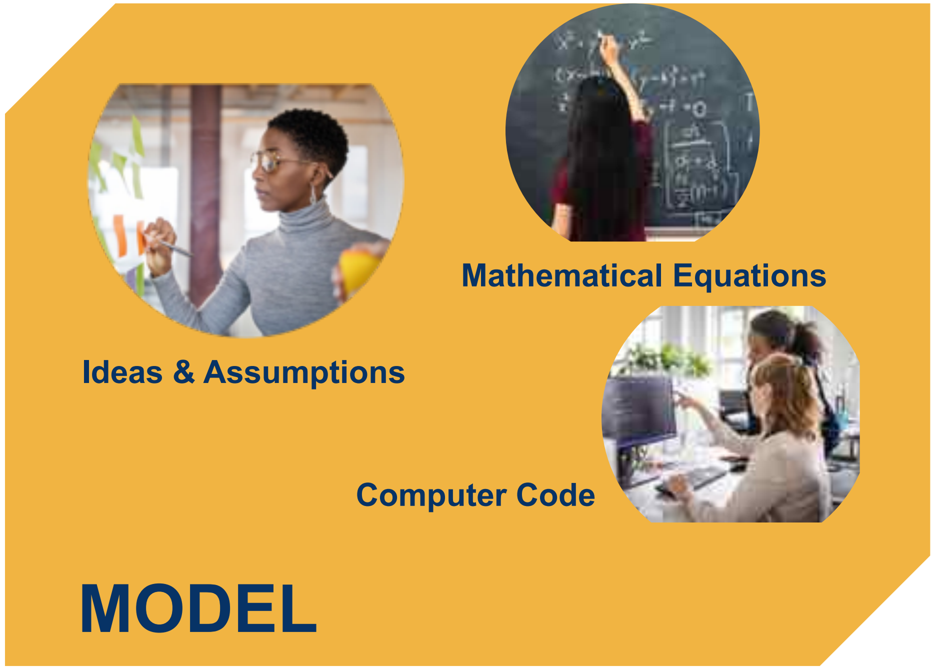

Now we can simulate the model

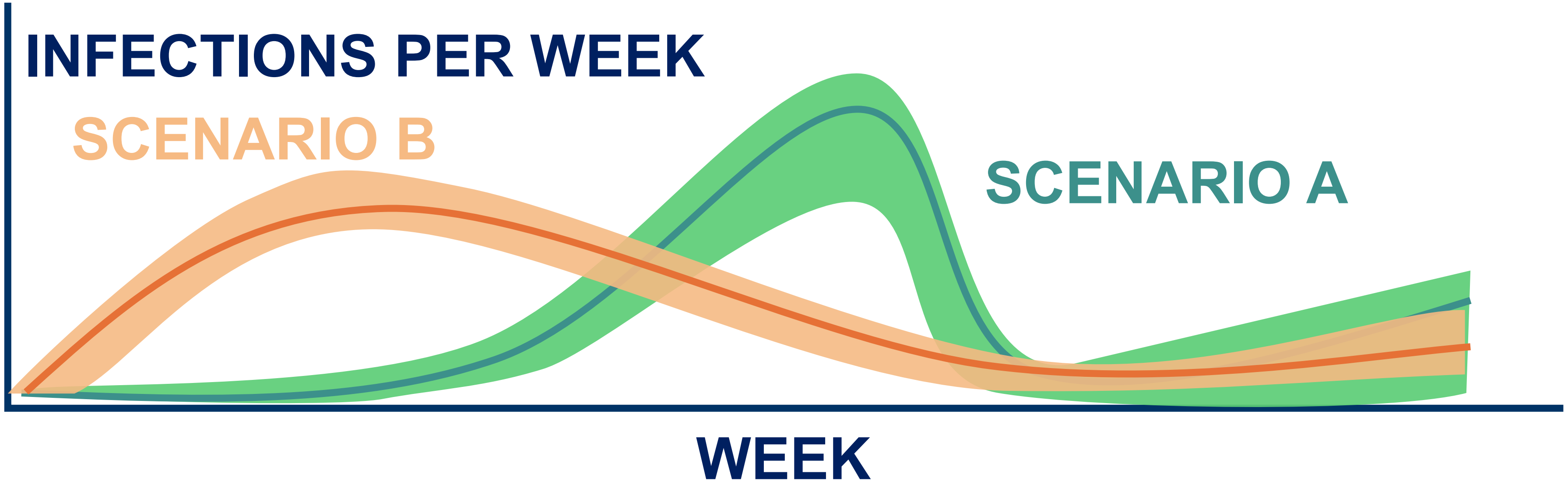

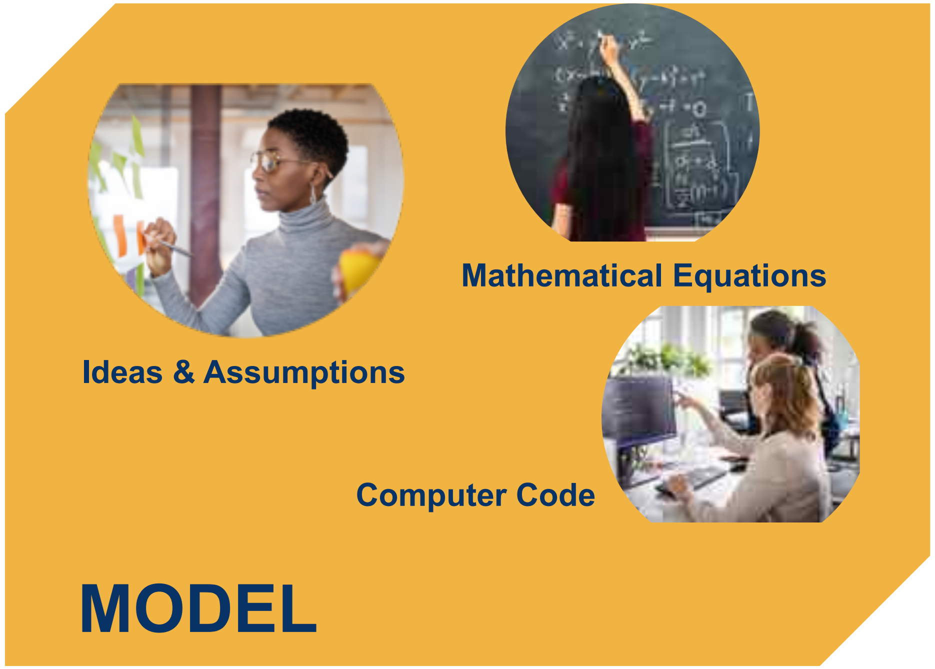

How is a model made useful for a specific place?

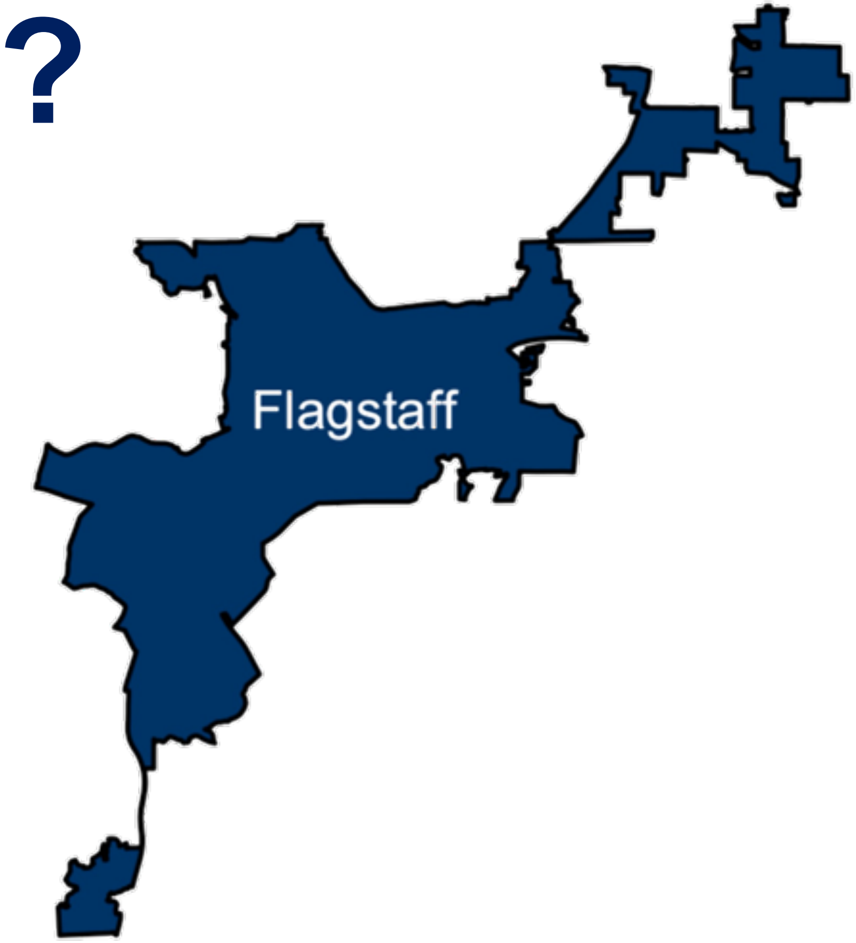

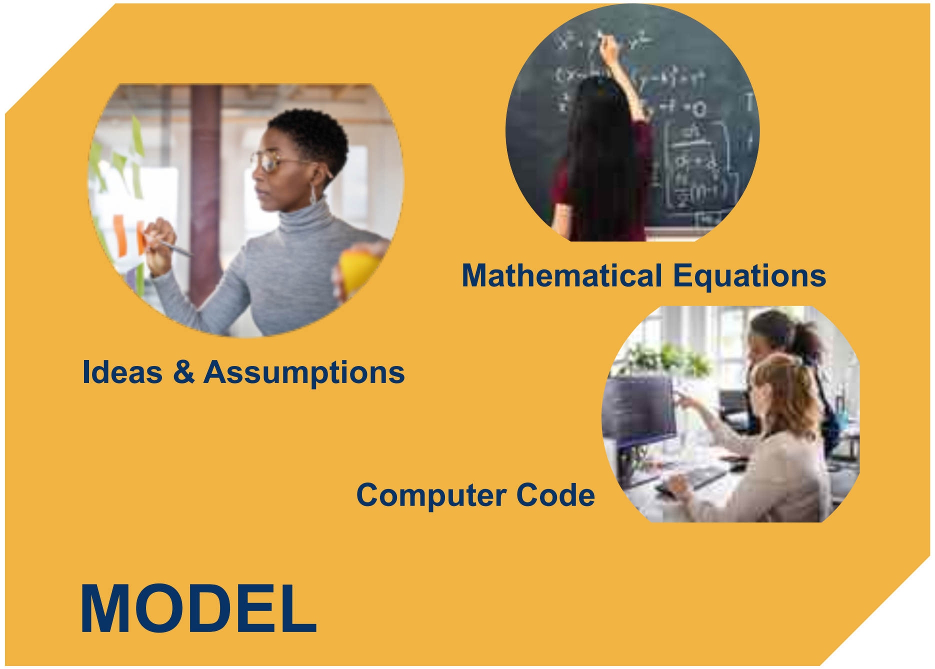

The model must be calibrated and refined.

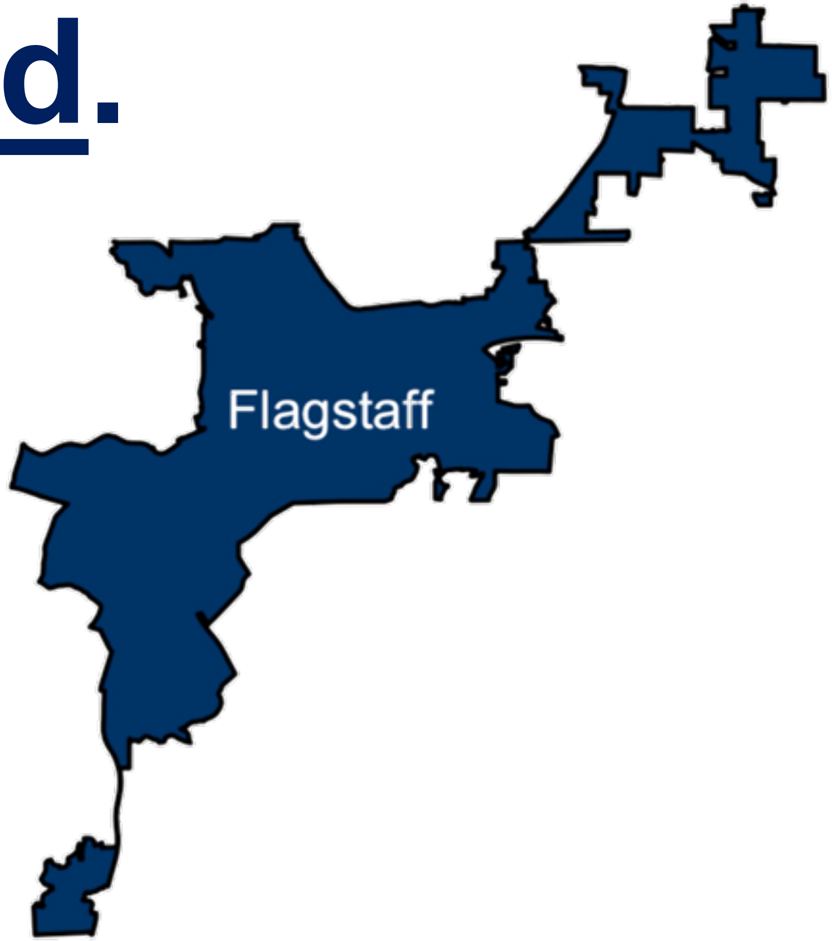

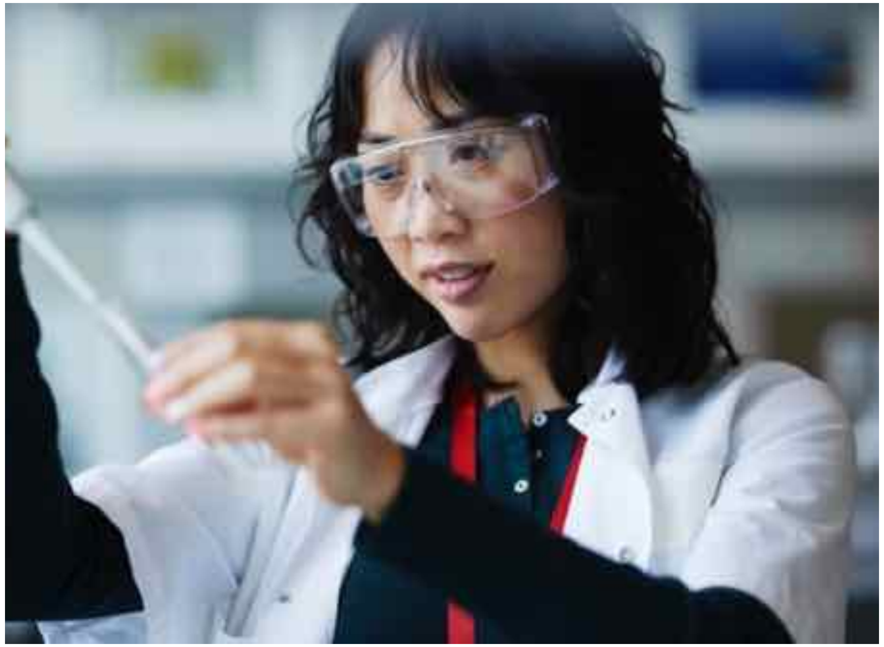

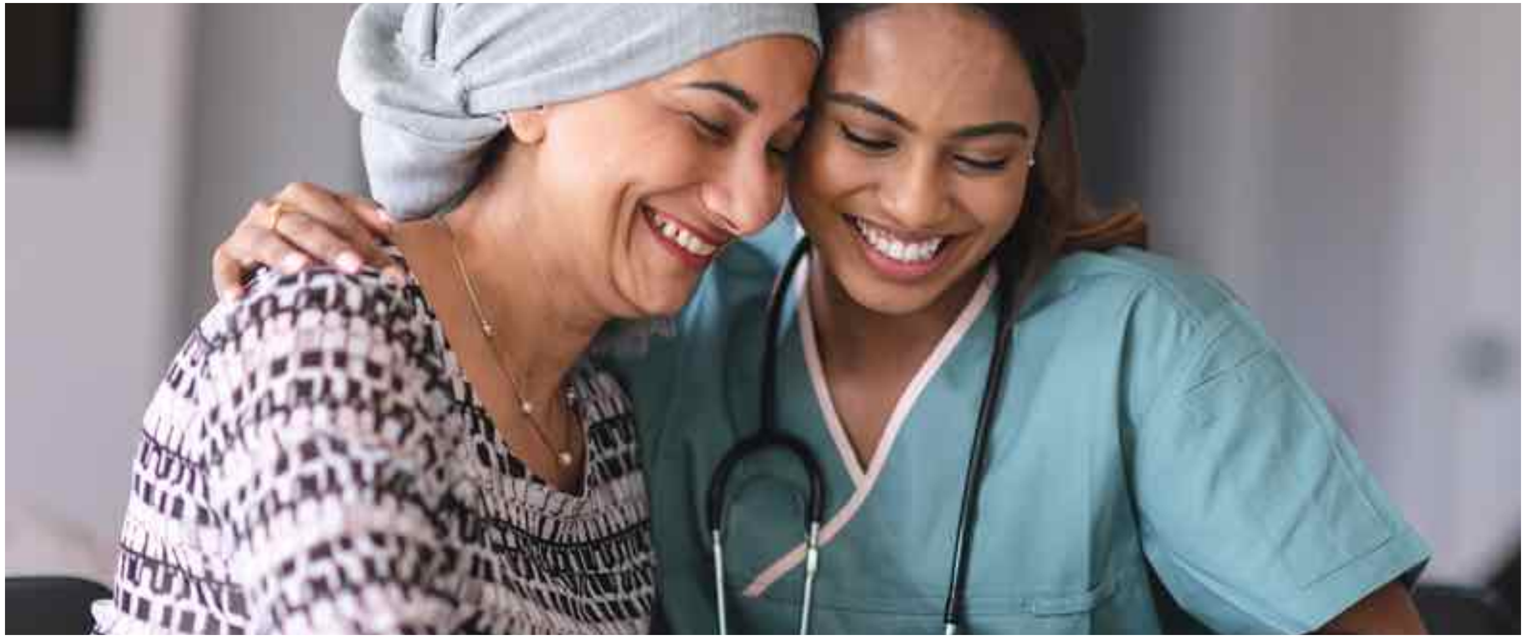

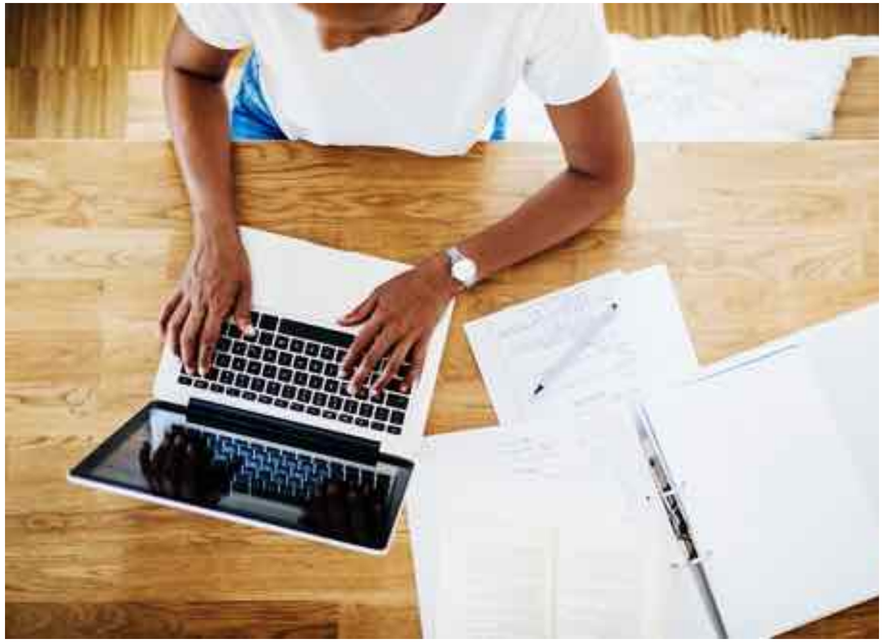

Data must be collected and entered.

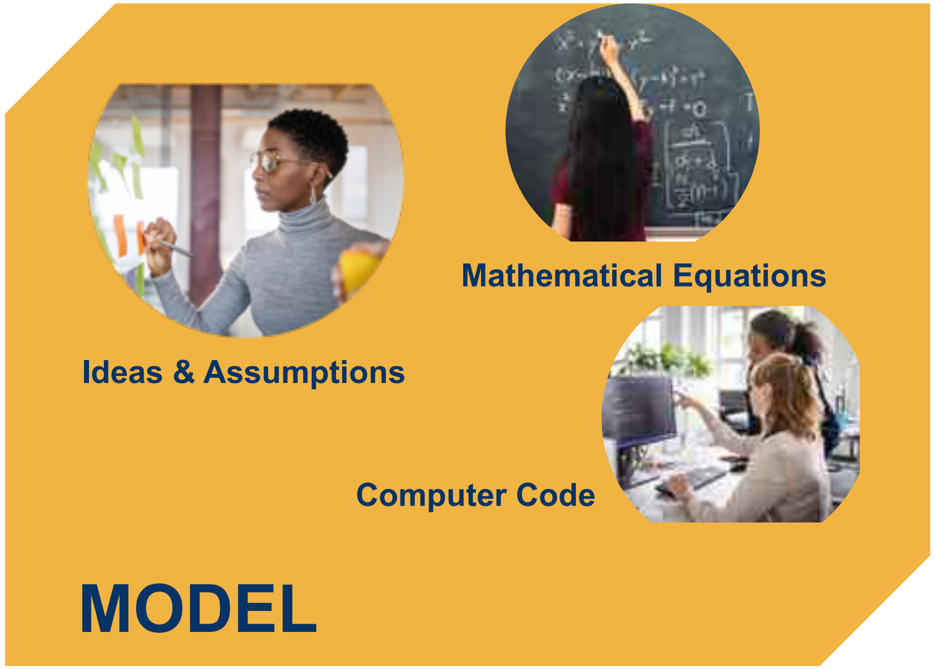

The model must be <u>calibrated</u> and <u>refined</u>.

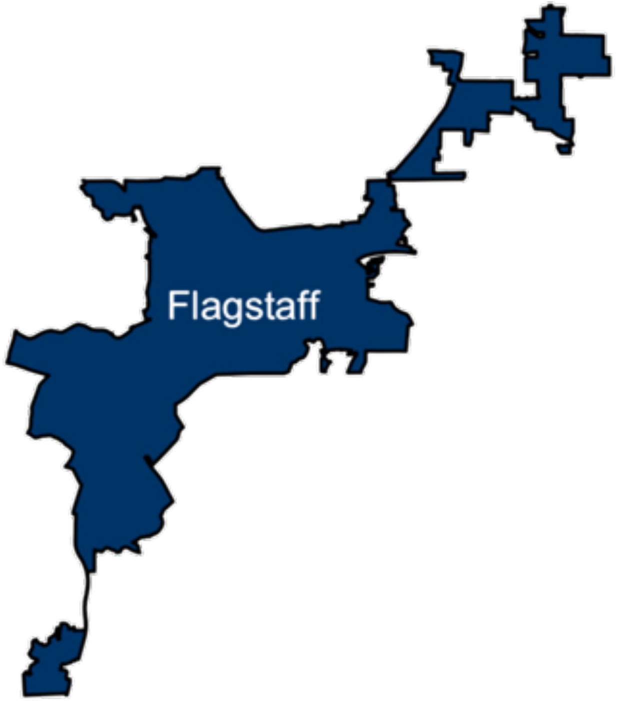

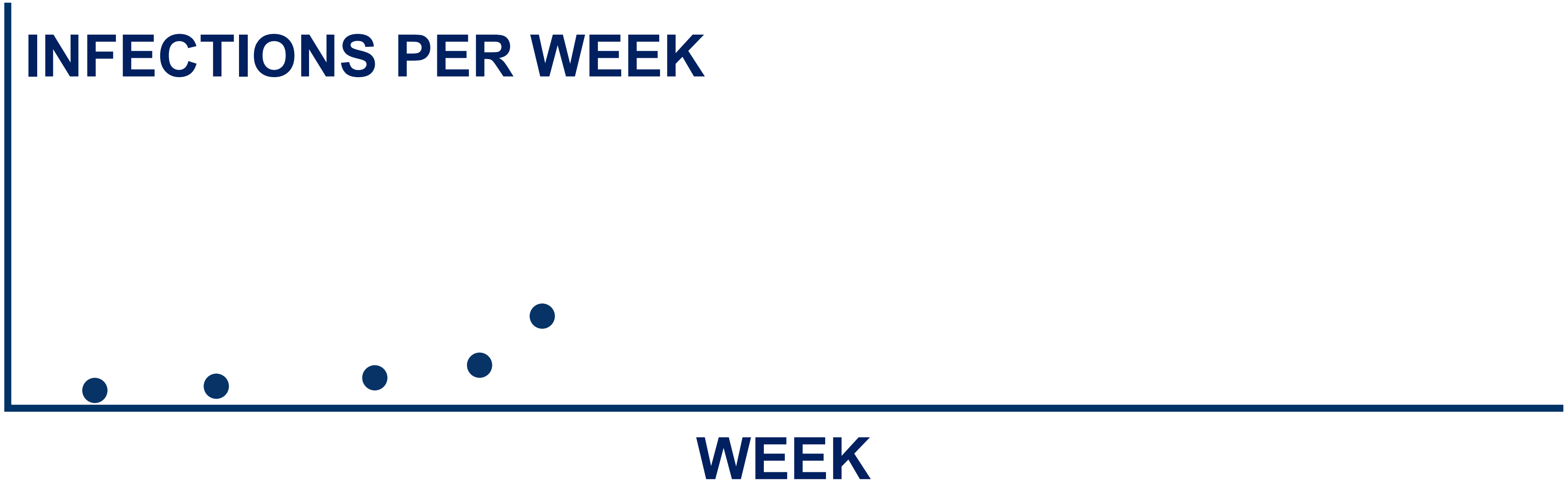

Calibrate models with data

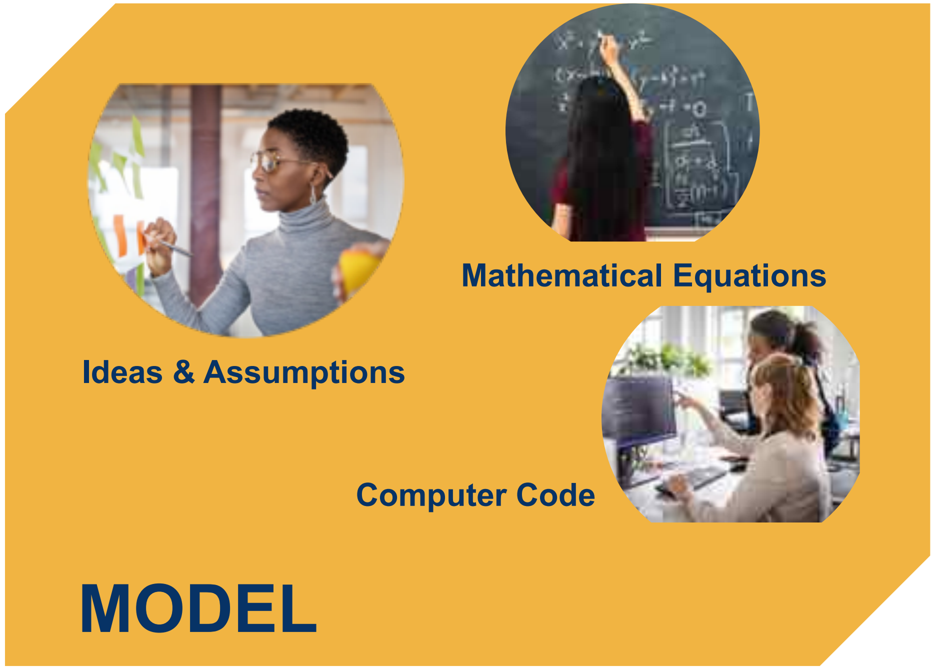

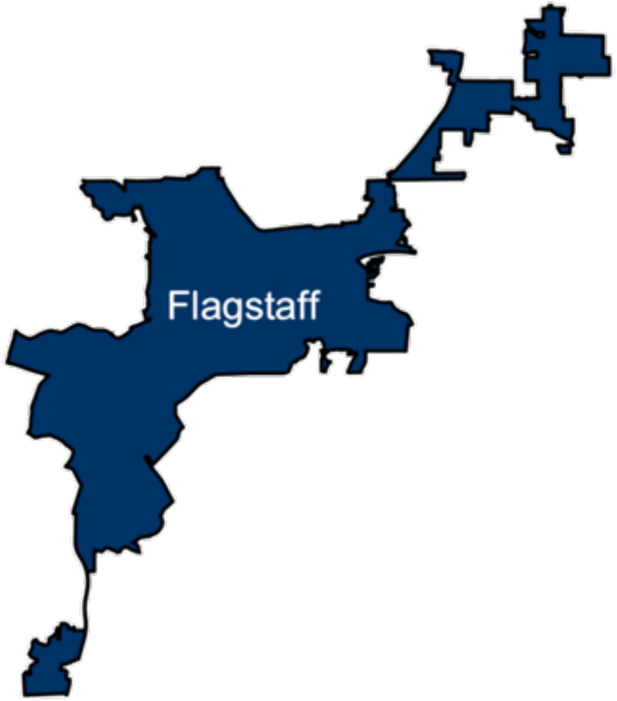

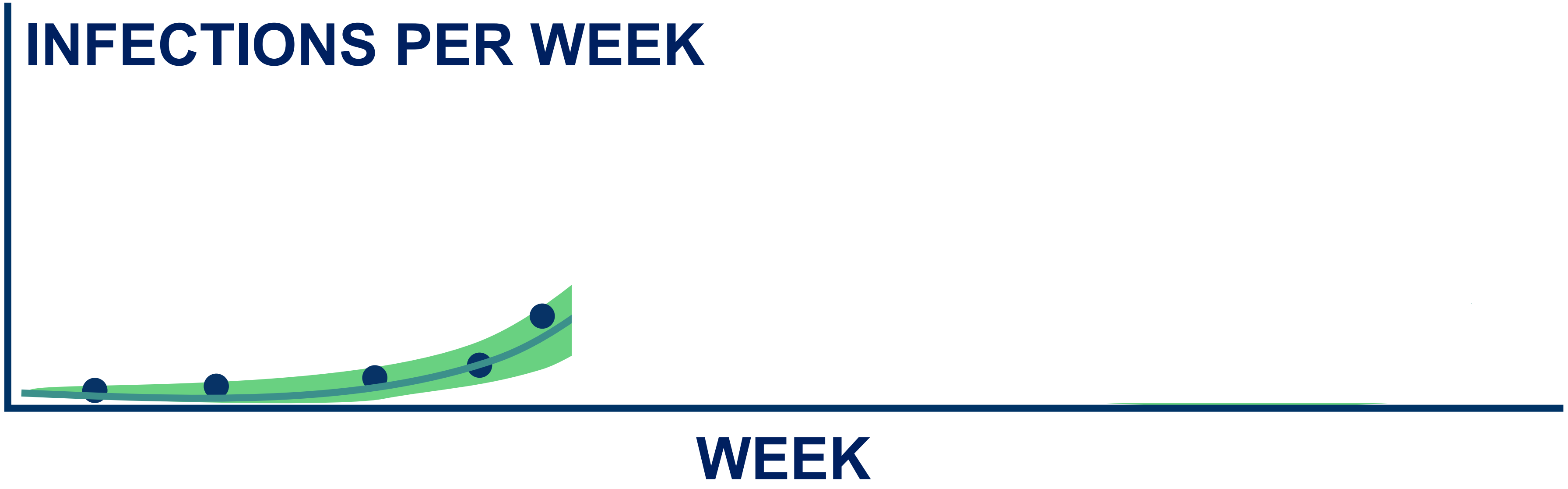

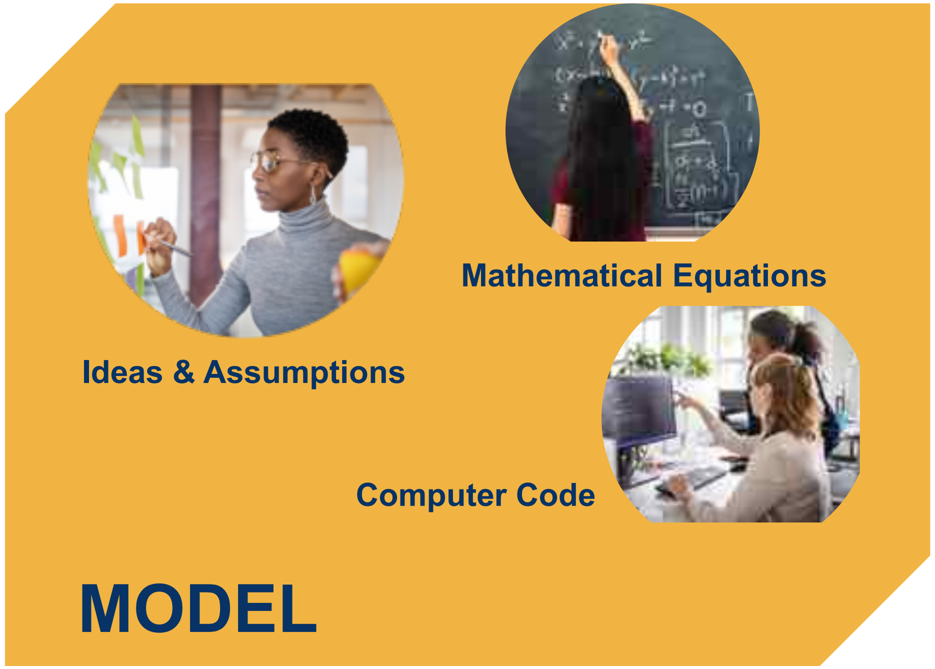

Need to refine models as new data emerge.

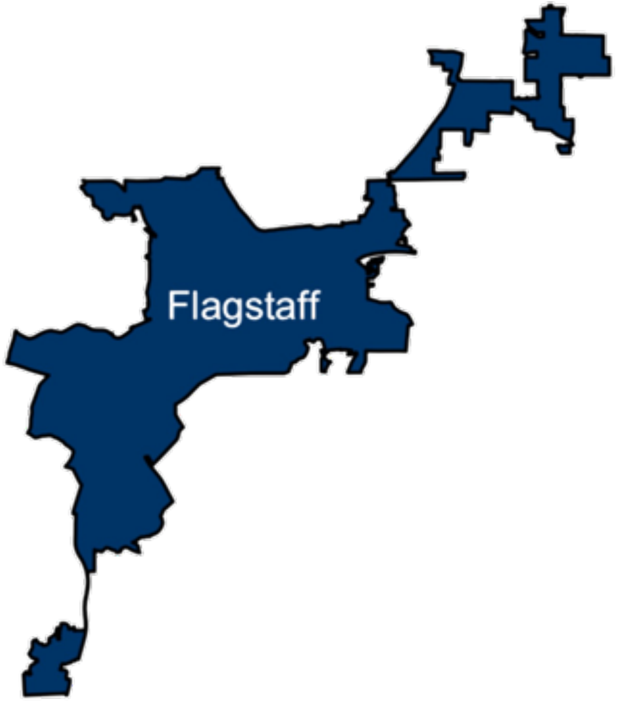

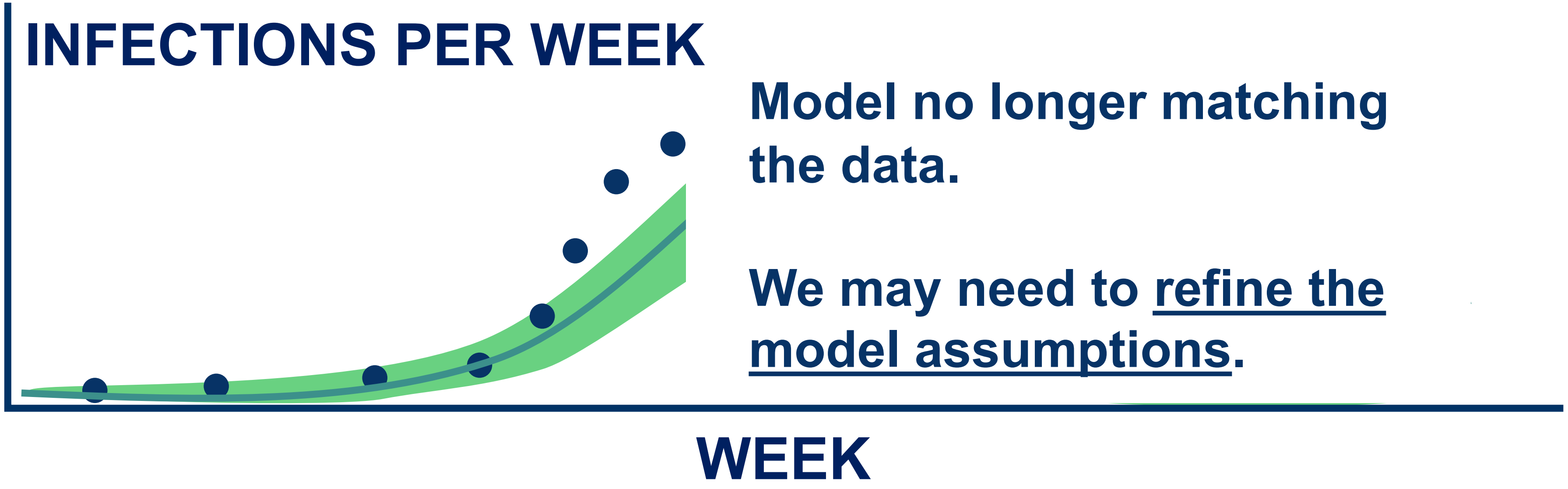

Model no longer matching the data.

We may need to refine the model assumptions.

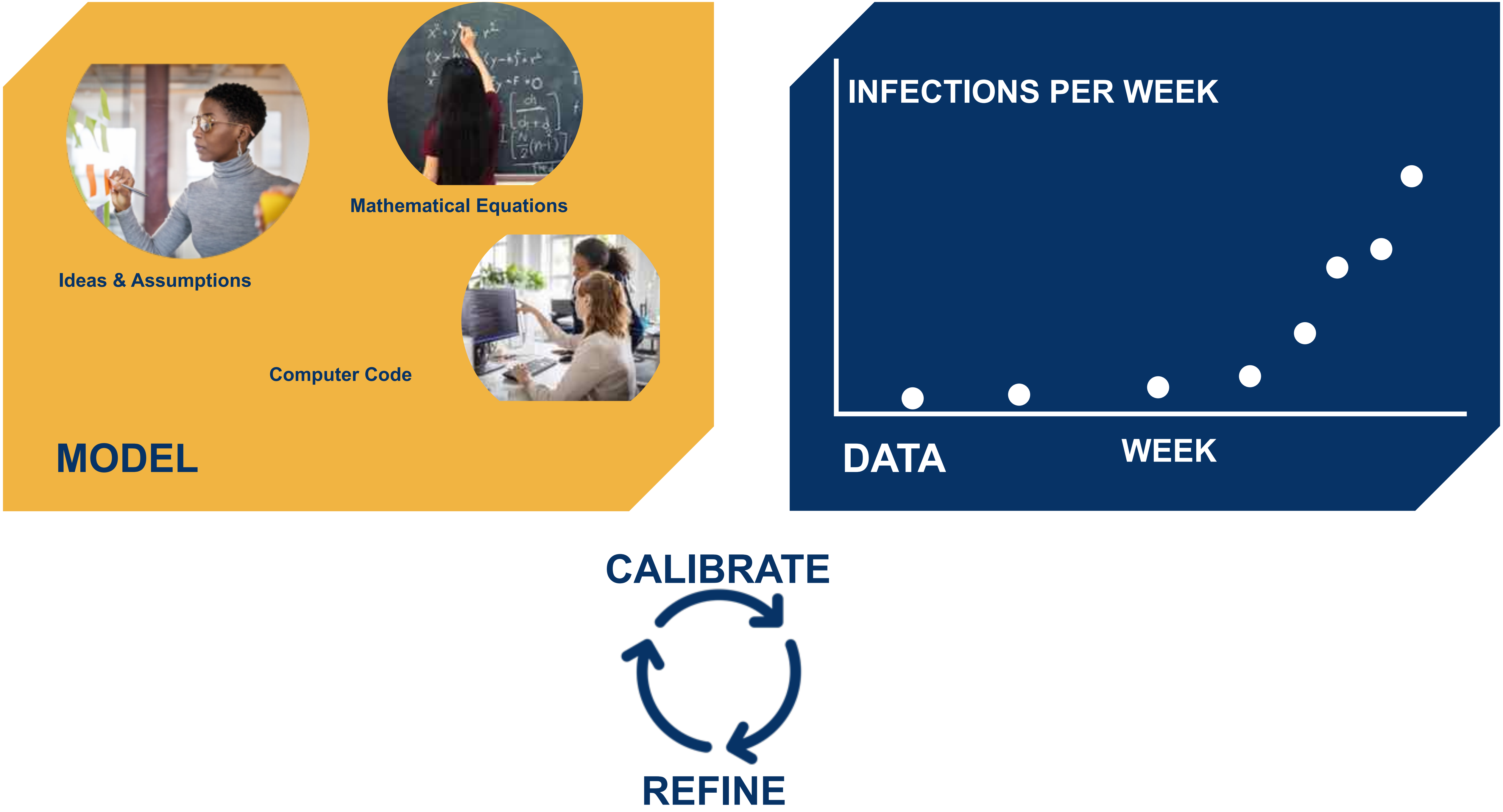
Ideas & Assumptions
Mathematical Equations
Computer Code
MODEL
INFECTIONS PER WEEK
WEEK
DATA
CALIBRATE
REFINE

This process can lead to a ‘useful’ model.

What can we do with a ‘useful’ model?

DISCOVERY

PREDICTION

SCENARIO SIMULATION

RESOURCE ALLOCATION

DISCOVERY

Which environmental factors drive infections?

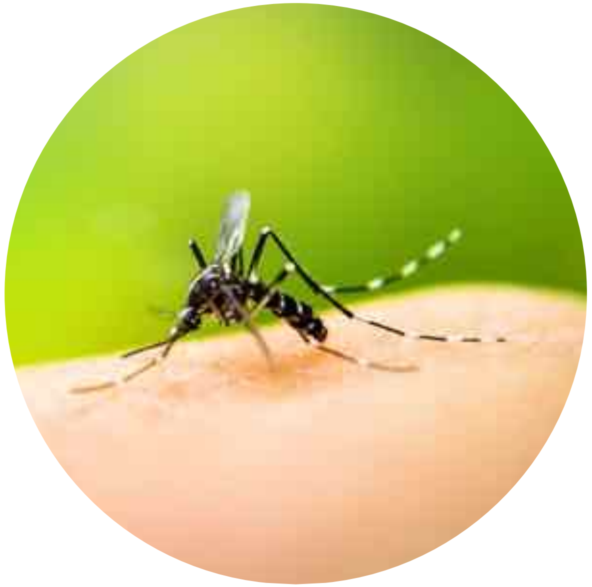

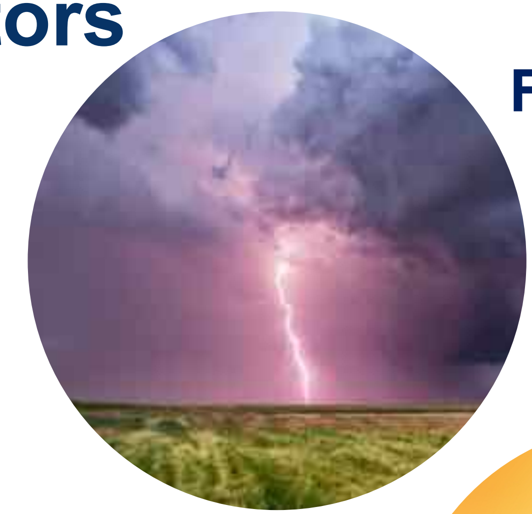

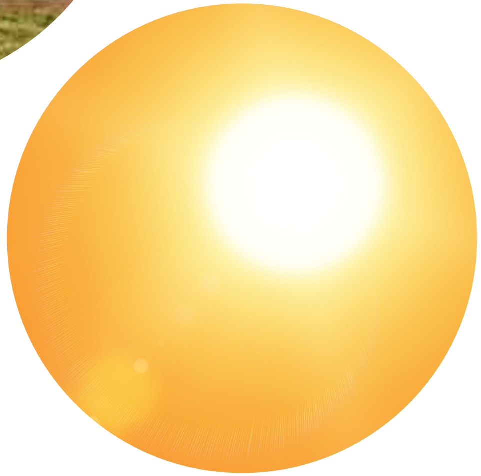

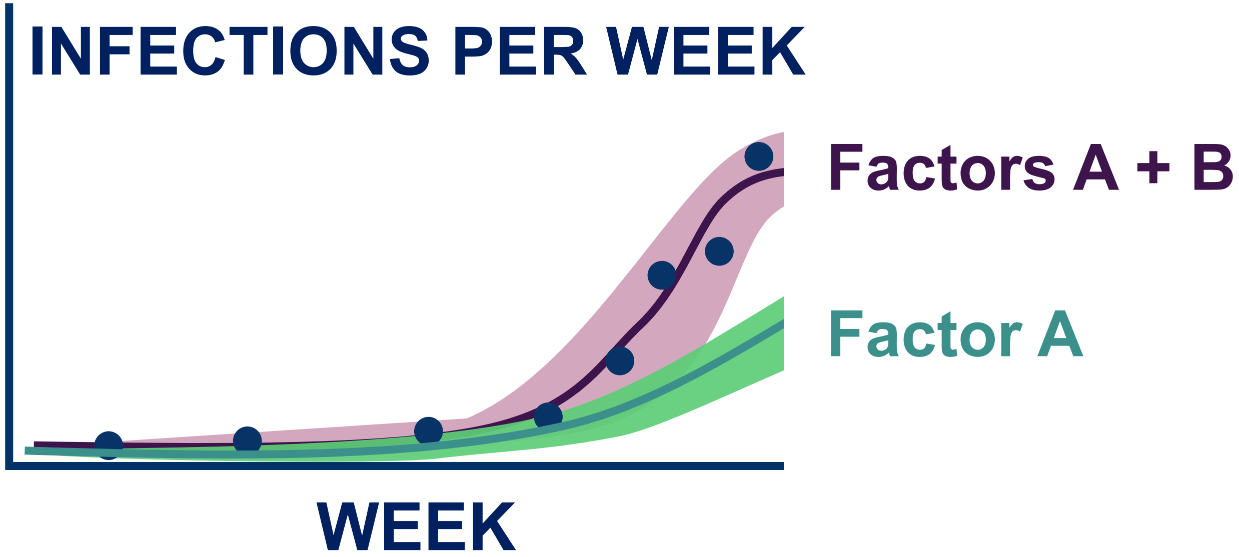

PREDICTION

What will happen in the next few <u>weeks</u>?

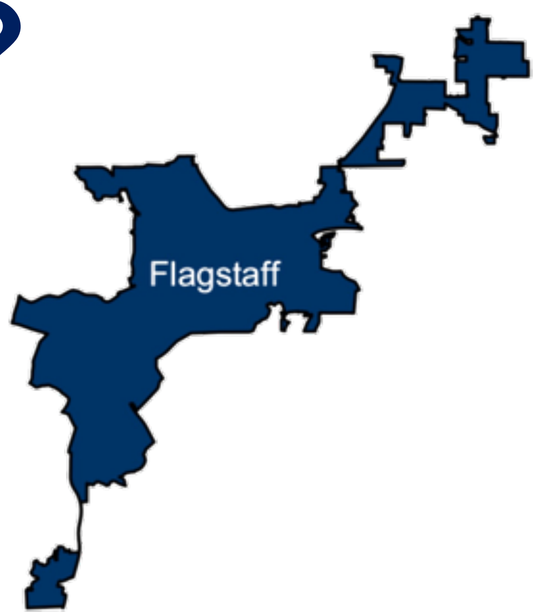

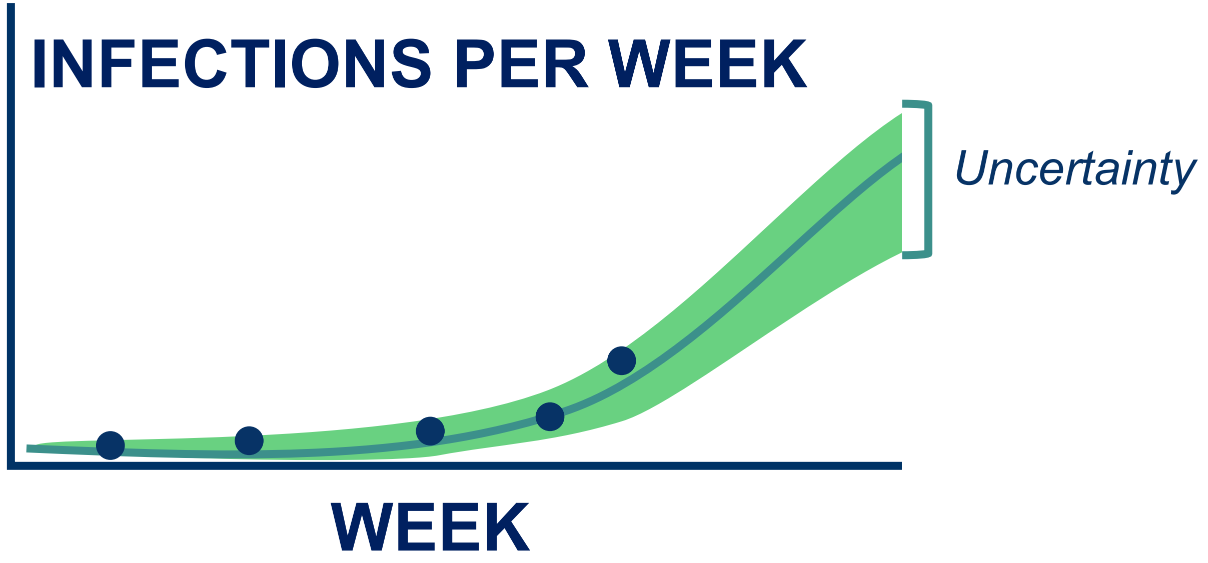

PREDICTION

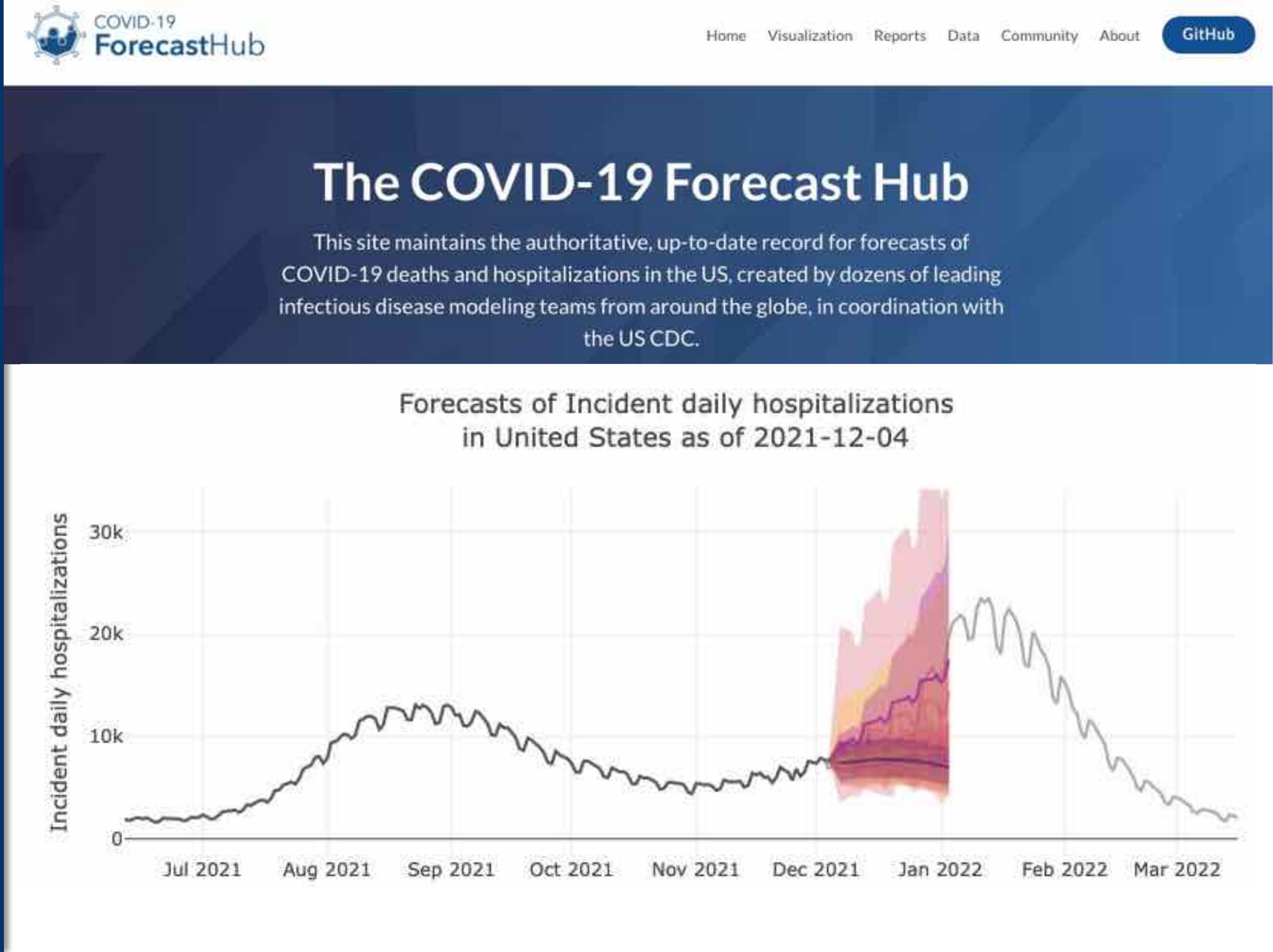
COVID-19
ForecastHub
Home
Visualization
Reports
Data
Community
About
GitHub
The COVID-19 Forecast Hub
This site maintains the authoritative, up-to-date record for forecasts of COVID-19 deaths and hospitalizations in the US, created by dozens of leading infectious disease modeling teams from around the globe, in coordination with the US CDC.
Forecasts of Incident daily hospitalizations
in United States as of 2021-12-04
Incident daily hospitalizations
30k
20k
10k
0
Jul 2021
Aug 2021
Sep 2021
Oct 2021
Nov 2021
Dec 2021
Jan 2022
Feb 2022
Mar 2022

PREDICTION

Where might the pathogen spread in the next few weeks?

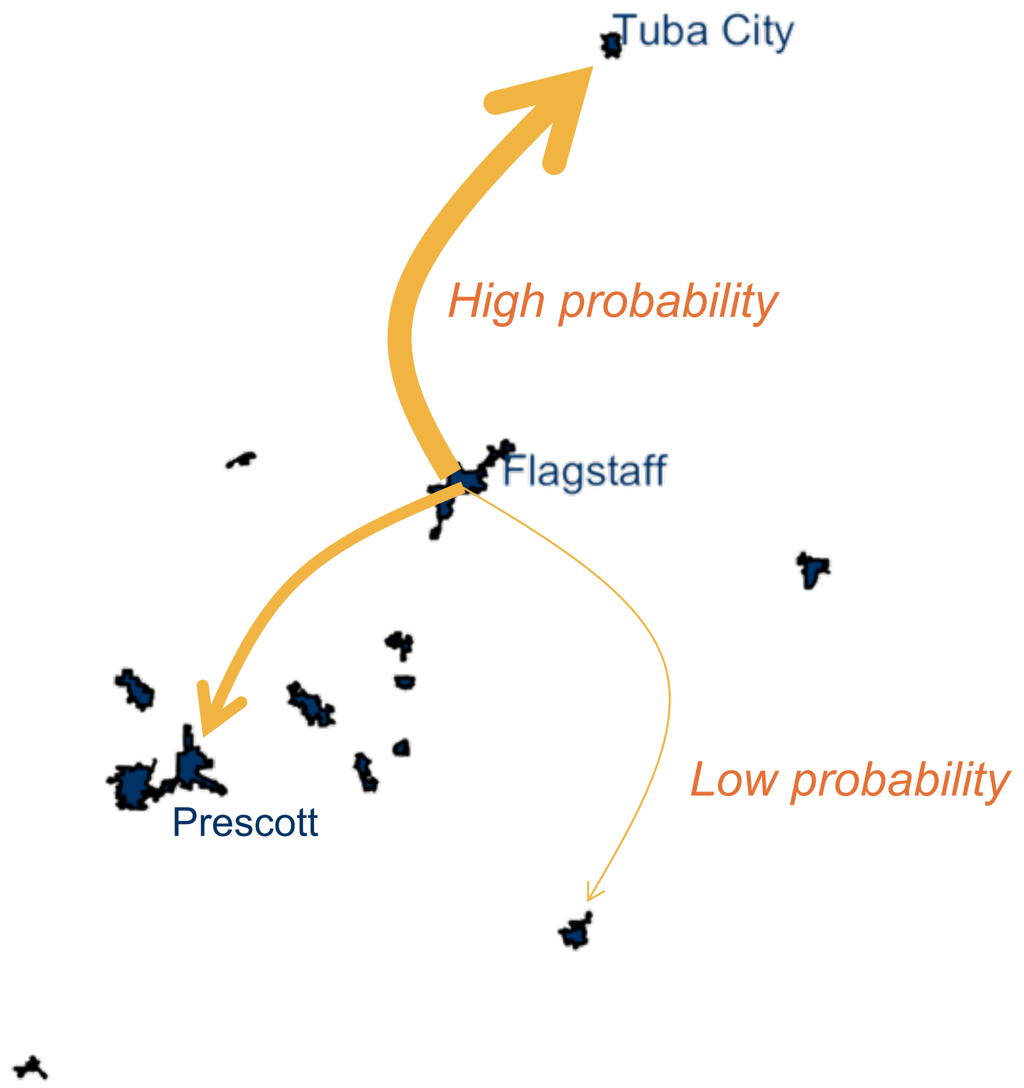

Where might the pathogen spread in the next few weeks?

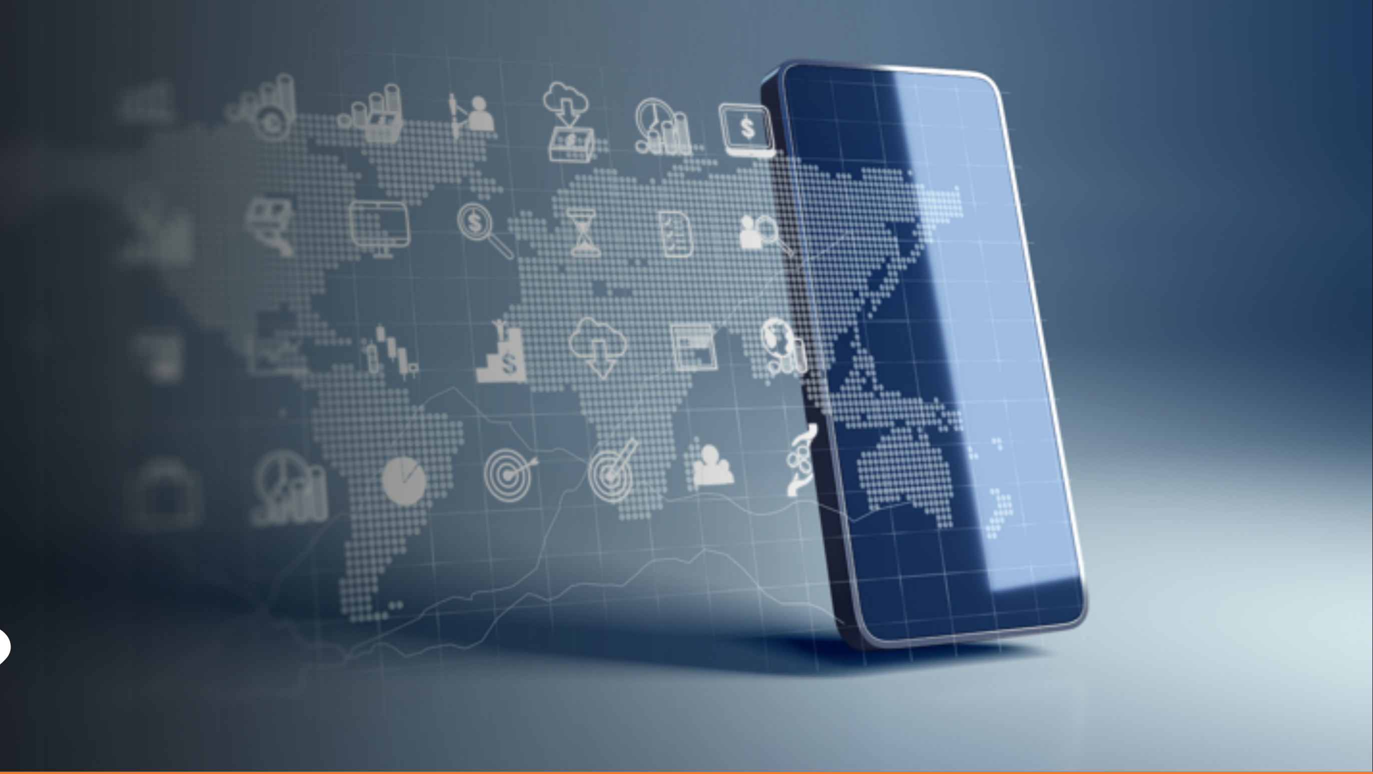

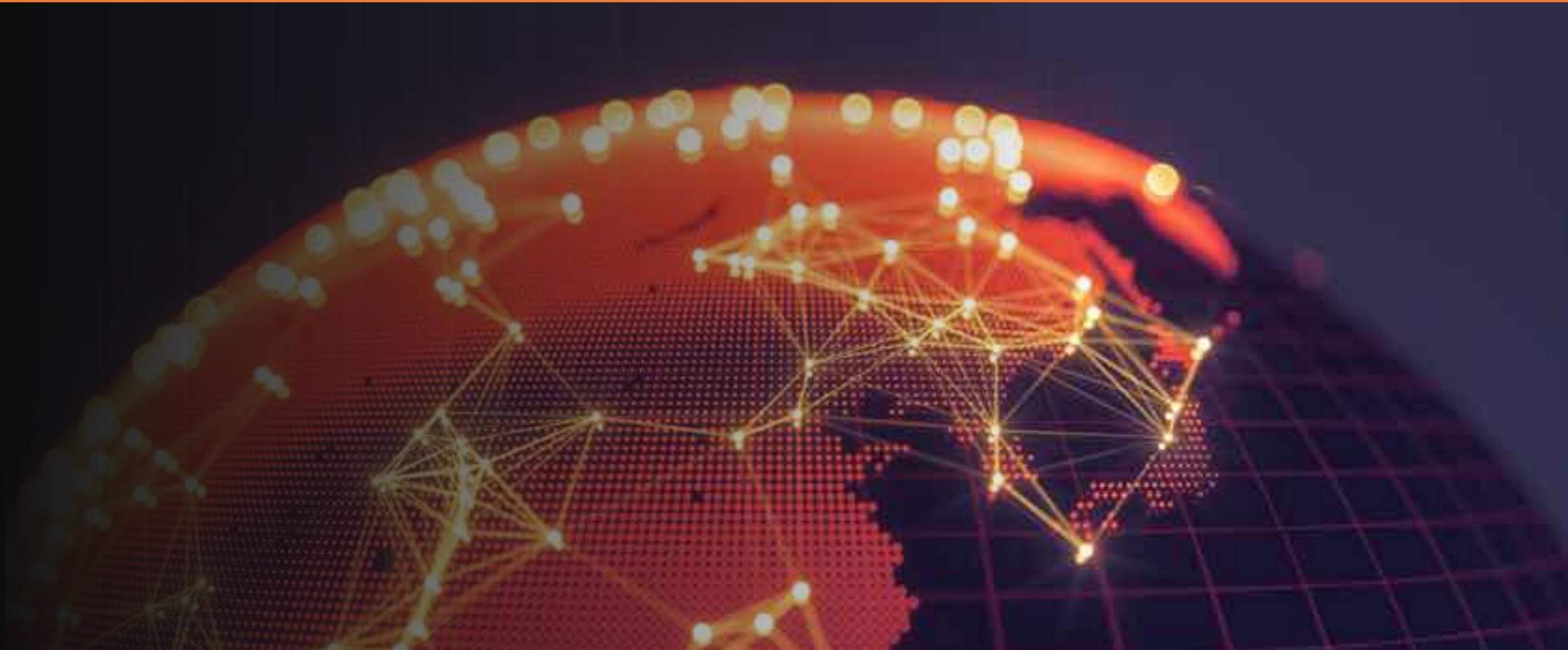

SCENARIO SIMULATION

What will happen in the next few months?

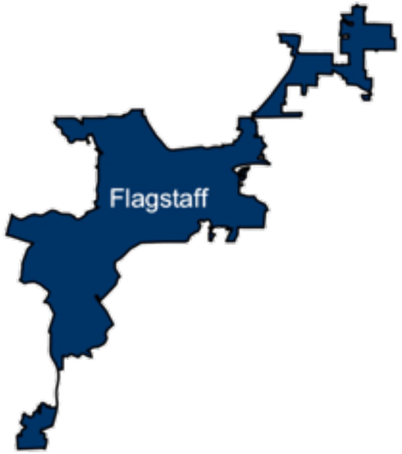

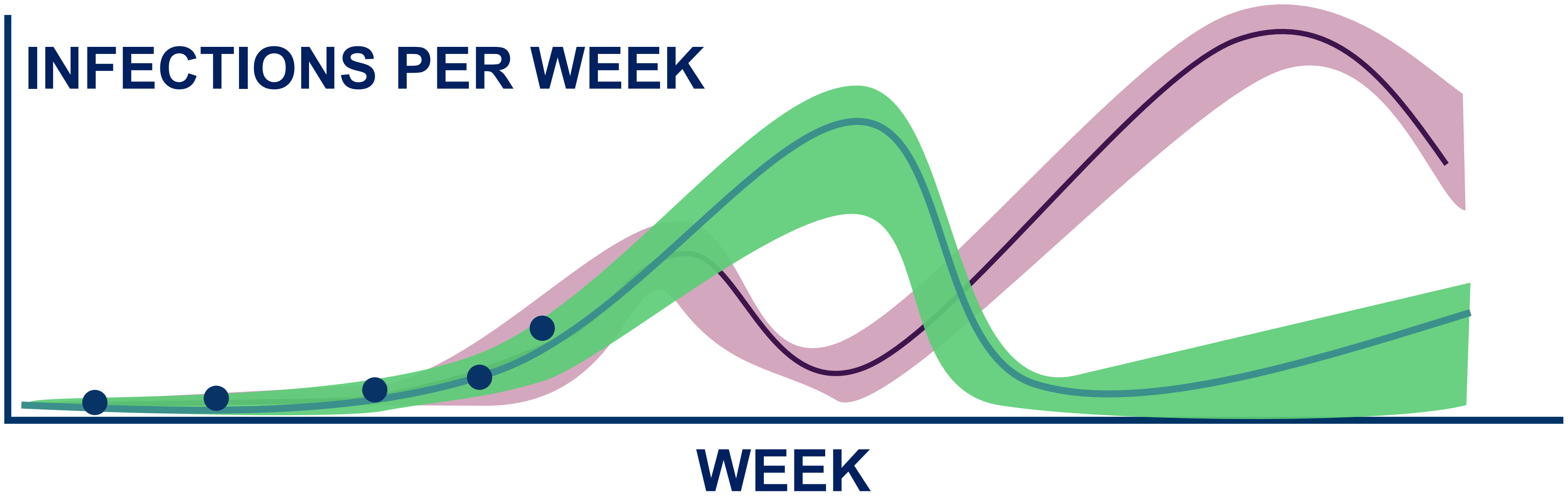

SCENARIO SIMULATION

What if we impose a certain <u>intervention</u>?

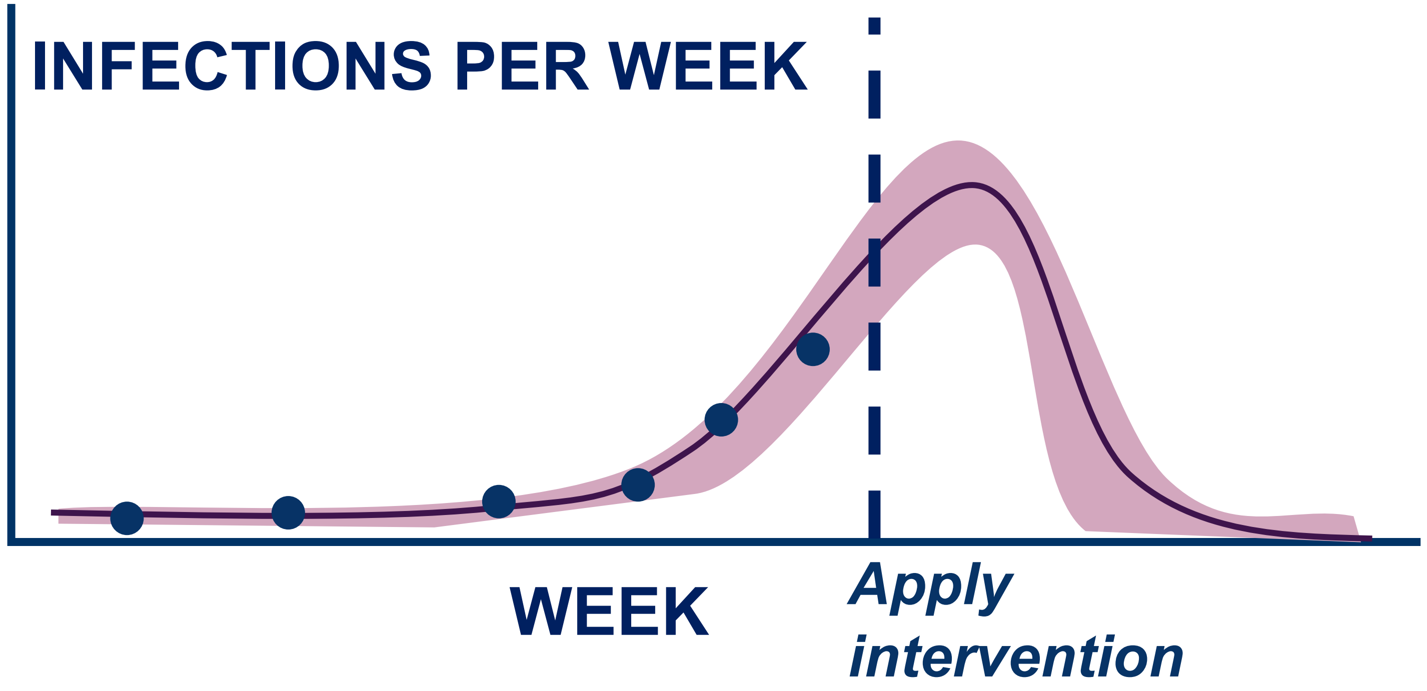

SCENARIO SIMULATION

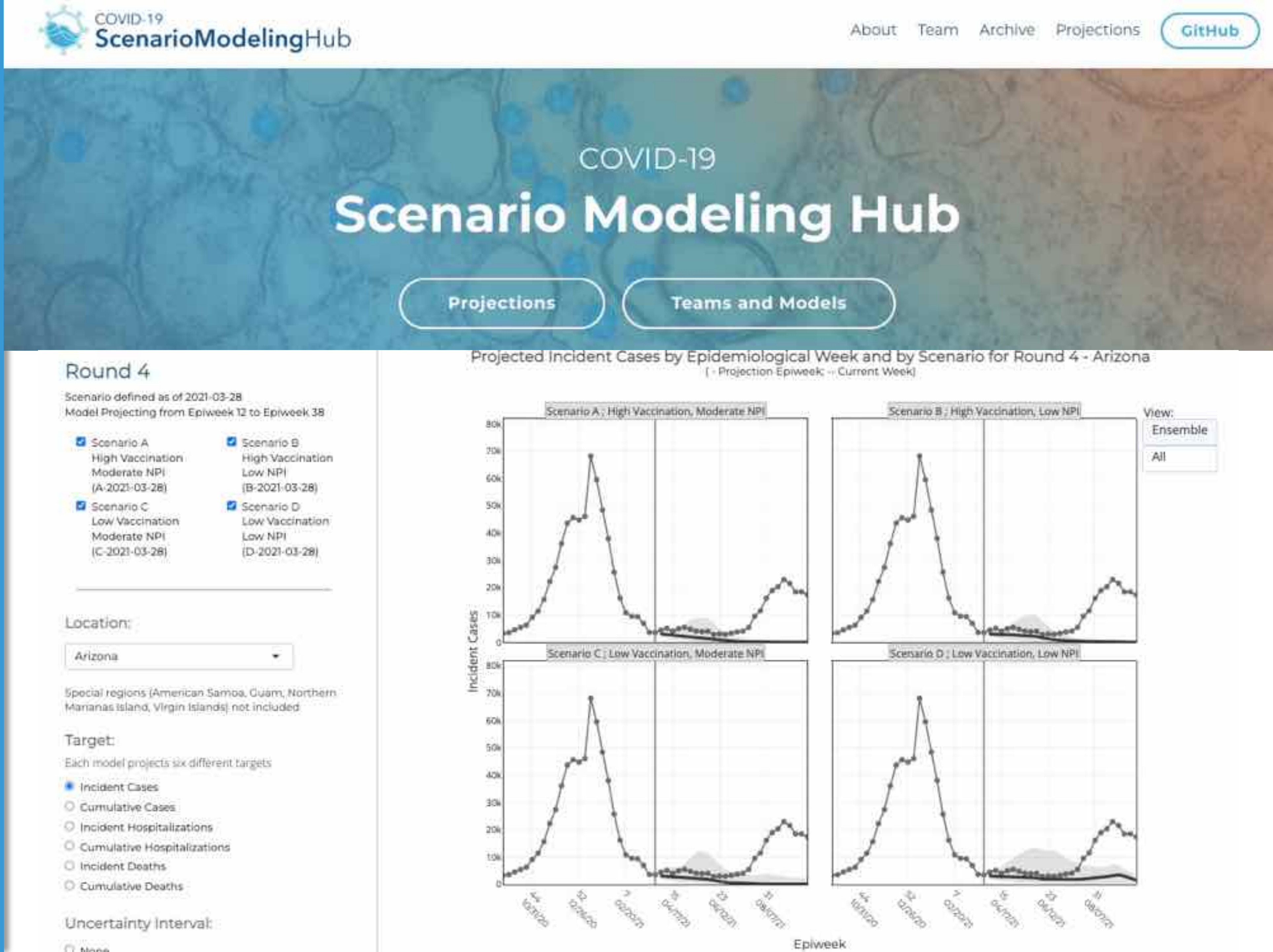

RESOURCE ALLOCATION

How do we deploy limited resources in the most optimized way to control specific outcomes?

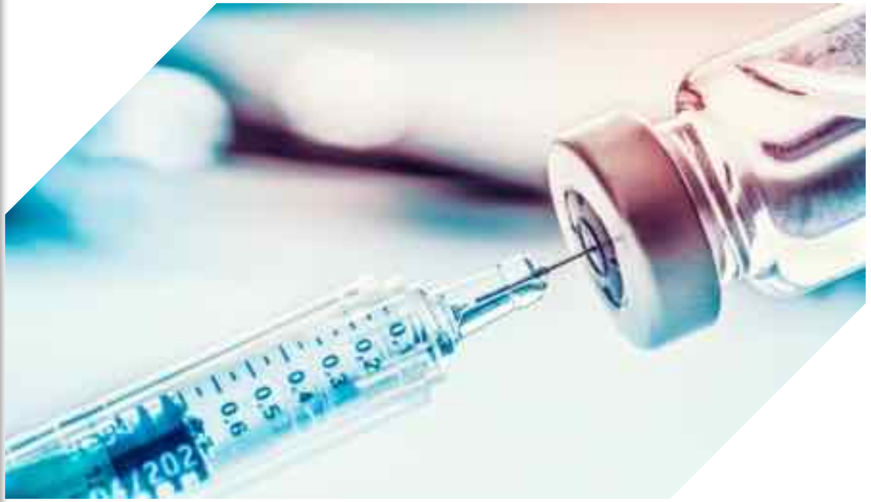

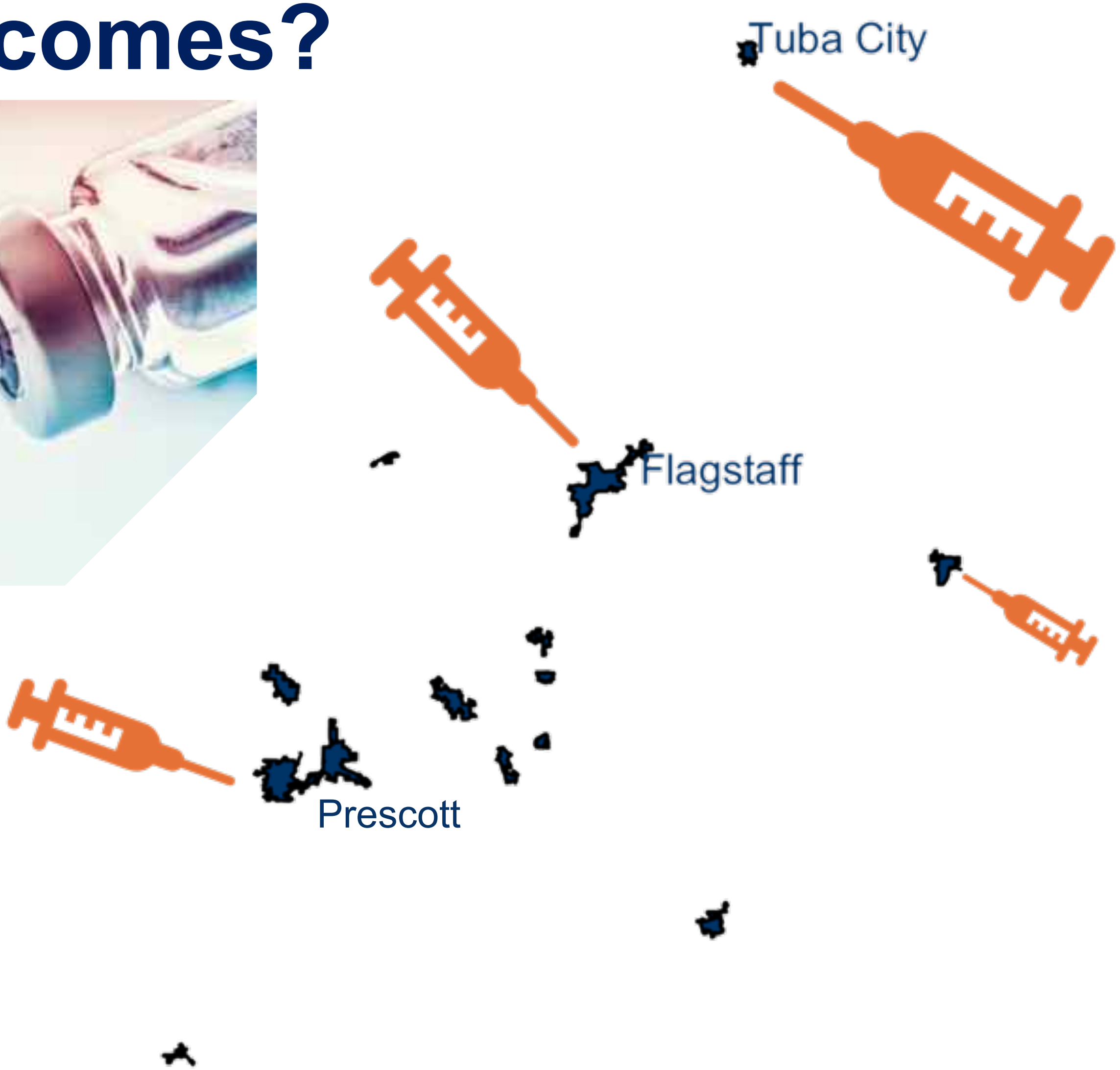

DISCOVERY

PREDICTION

SCENARIO SIMULATION

RESOURCE ALLOCATION

Epidemiological modeling can provide another layer of quality information to support decision-making.

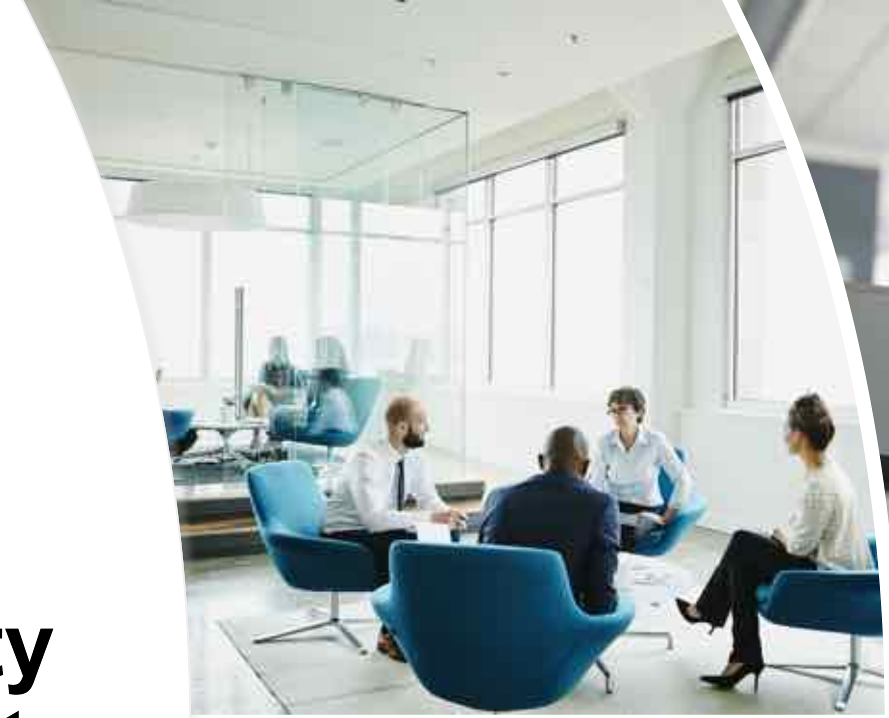

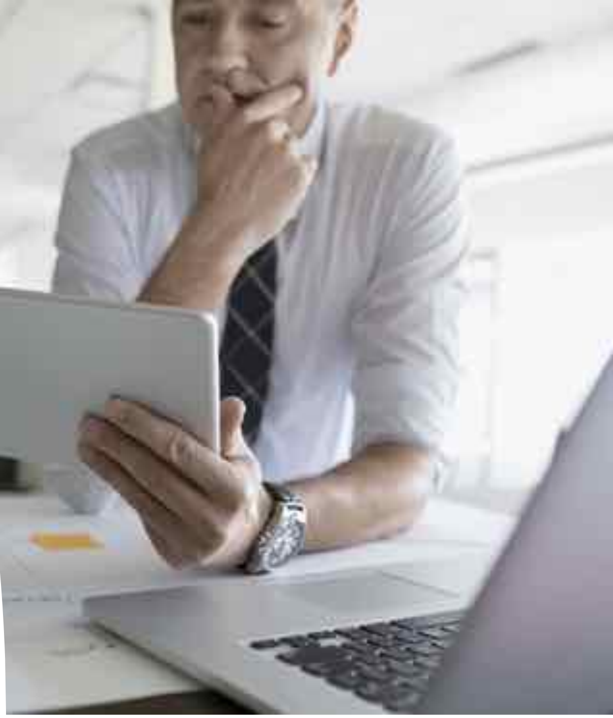

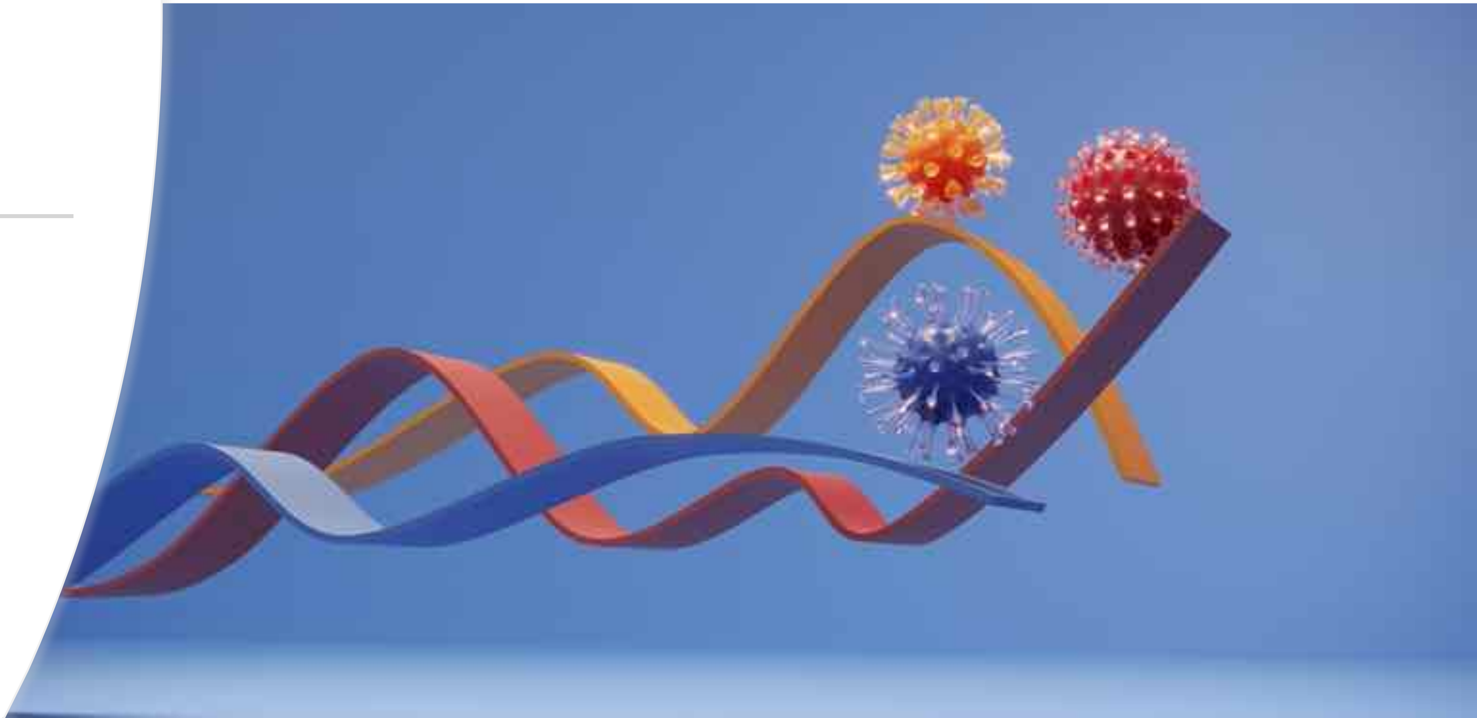

Deploying epidemiological modeling can be very useful, but not without challenges.

LOTS OF MODELS

- *Which model is best for my jurisdiction?*

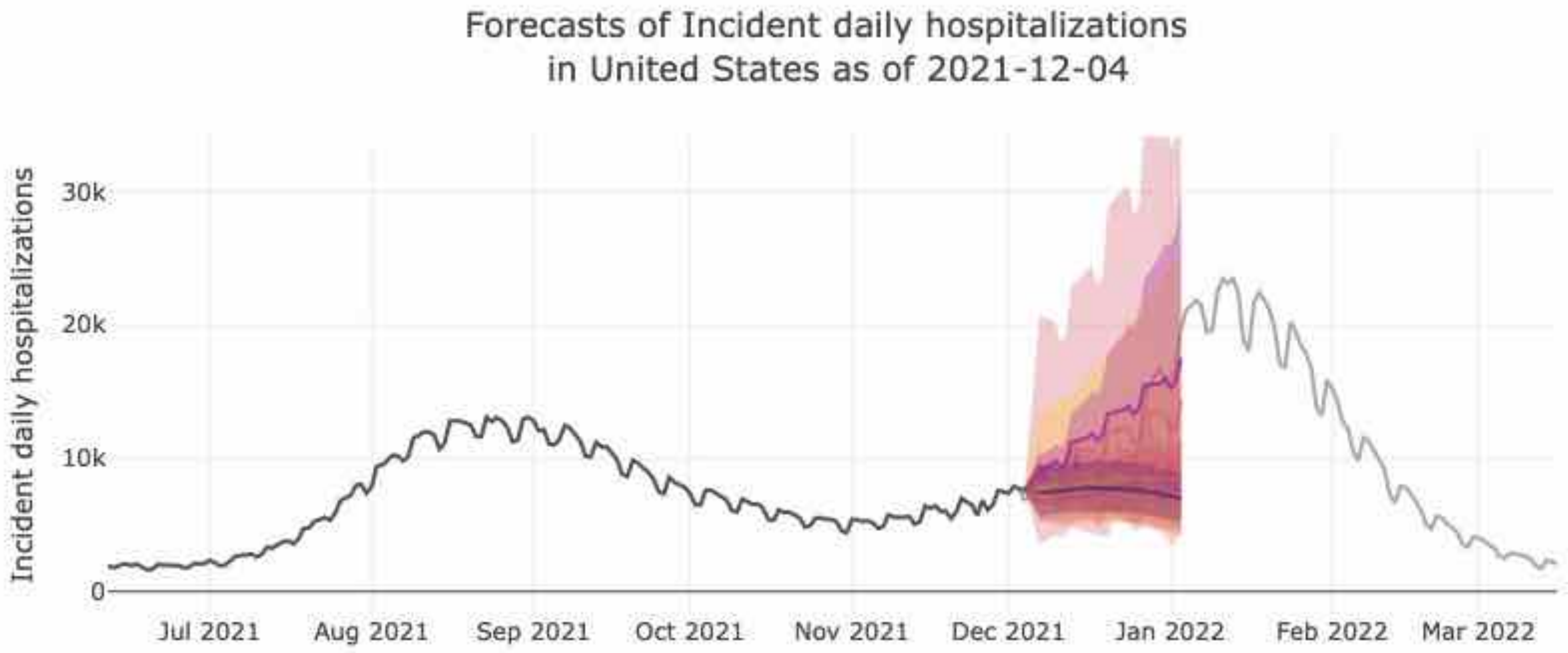

SPATIAL SCALE OF MODEL PREDICTIONS

- *If predictions are made for a 'county', are these relevant for a 'city'?*

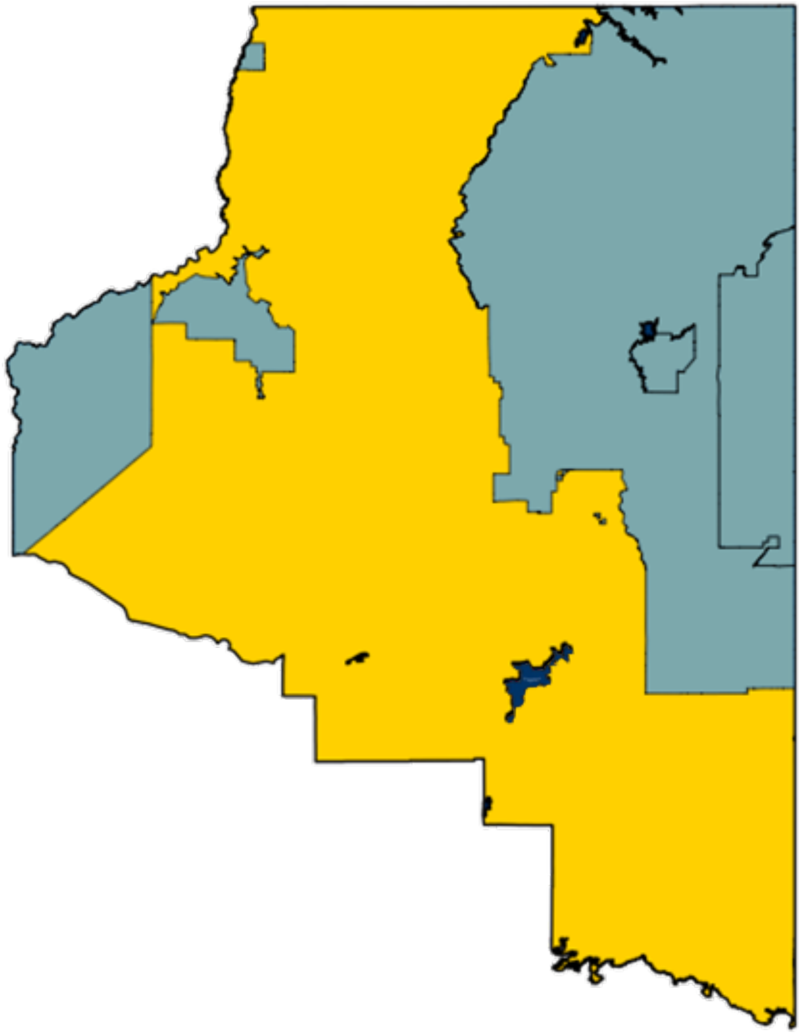

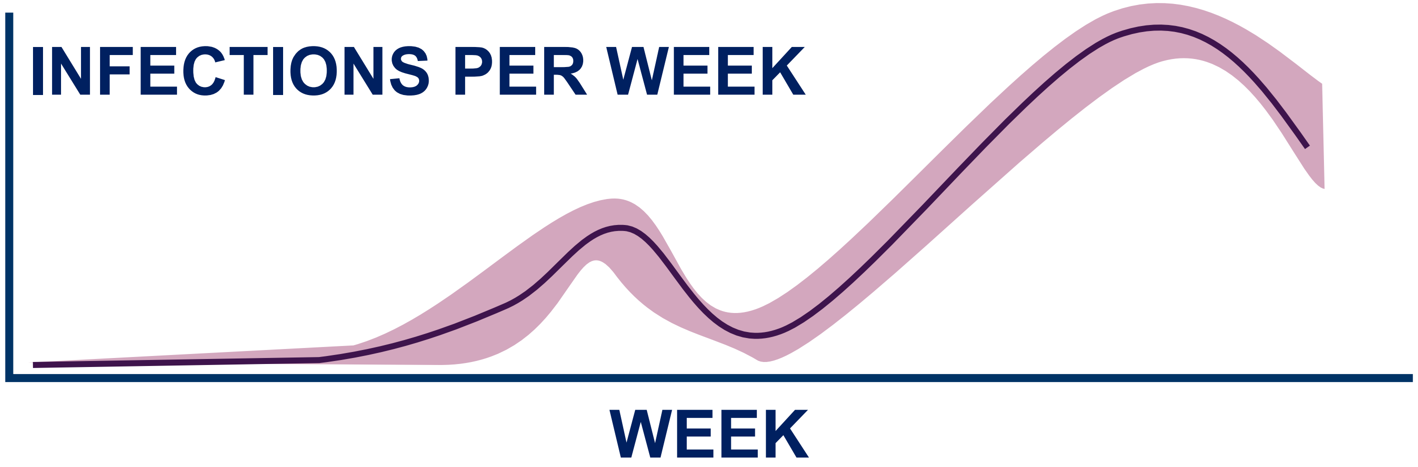

SPATIAL SCALE OF MODEL PREDICTIONS

- *If predictions are made for a 'county', are these relevant for a 'city'?*

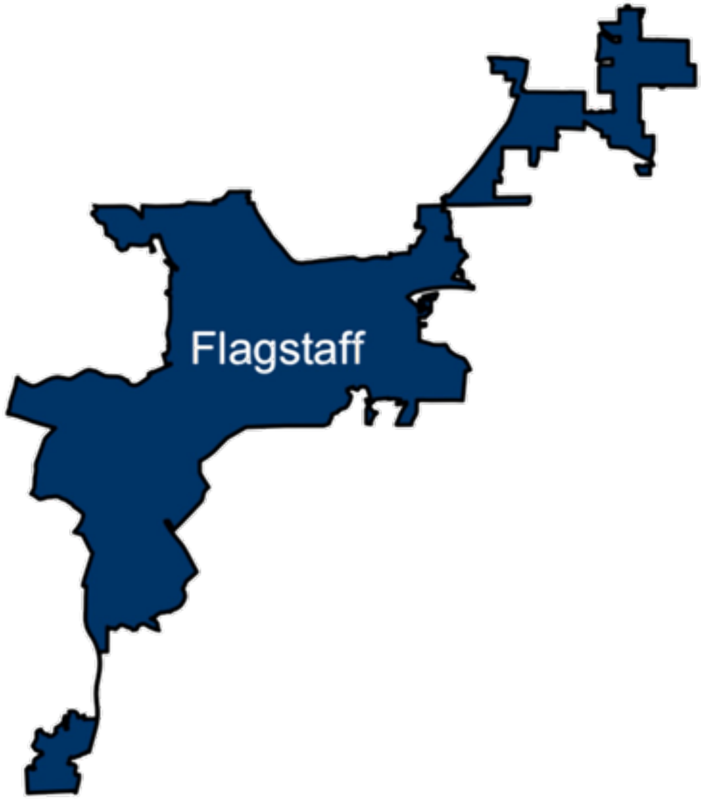

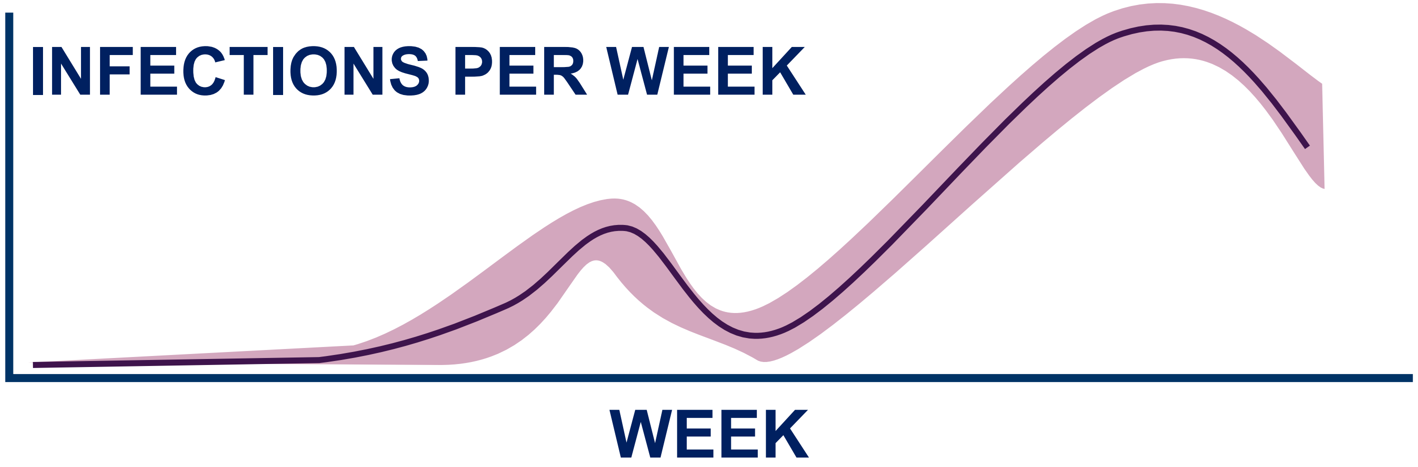

Is this same prediction relevant?

SPATIAL SCALE OF MODEL PREDICTIONS

- ***How do we make predictions for a ‘city’, if data are only available at the scale of ‘county’?***

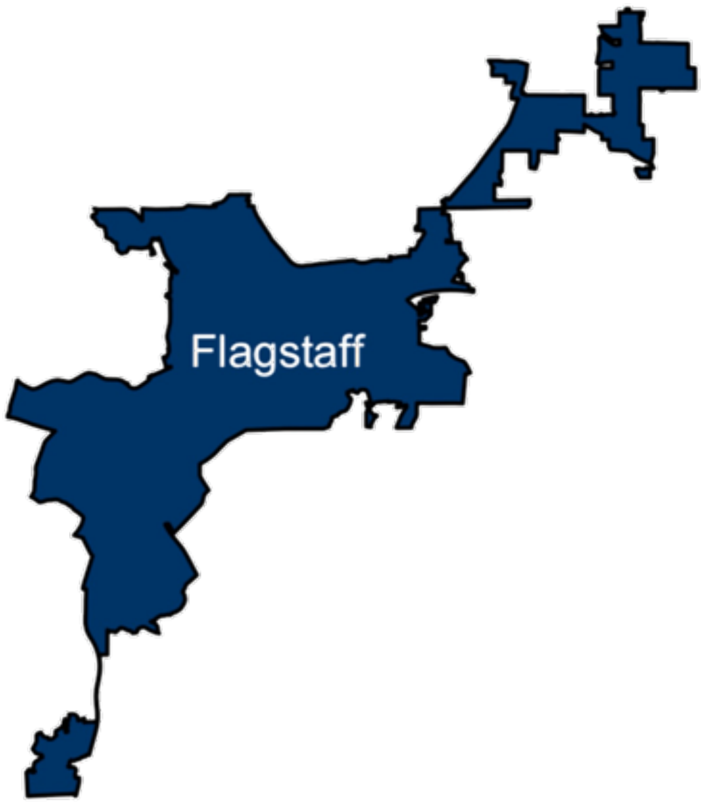

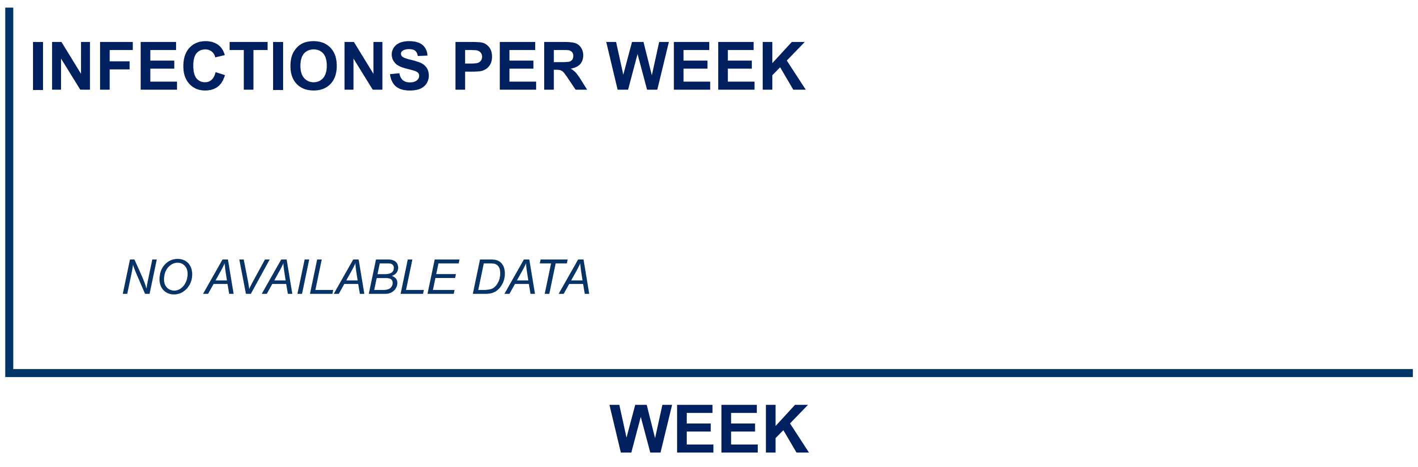

SPATIAL SCALE OF MODEL PREDICTIONS

- *How do we make predictions for a ‘city’, if data are only available at the scale of ‘county’?*

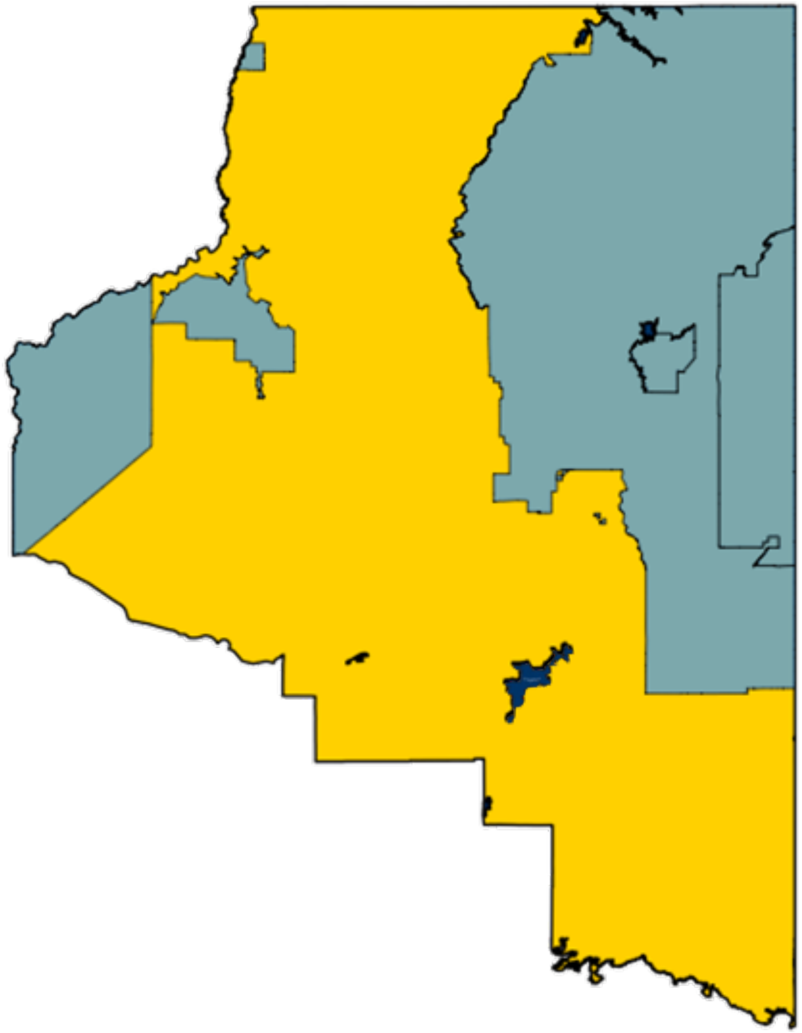

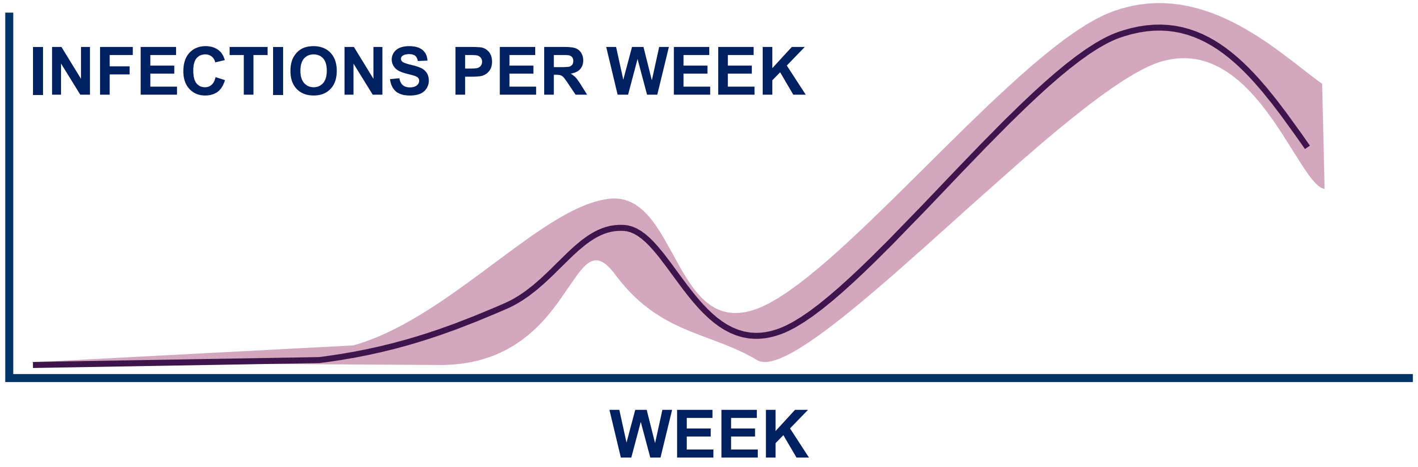

Is this the finest scale we can predict, given data availability?

PROBLEMS WITH DATA

- *What if data quality and quantity vary across locations, and over time?*

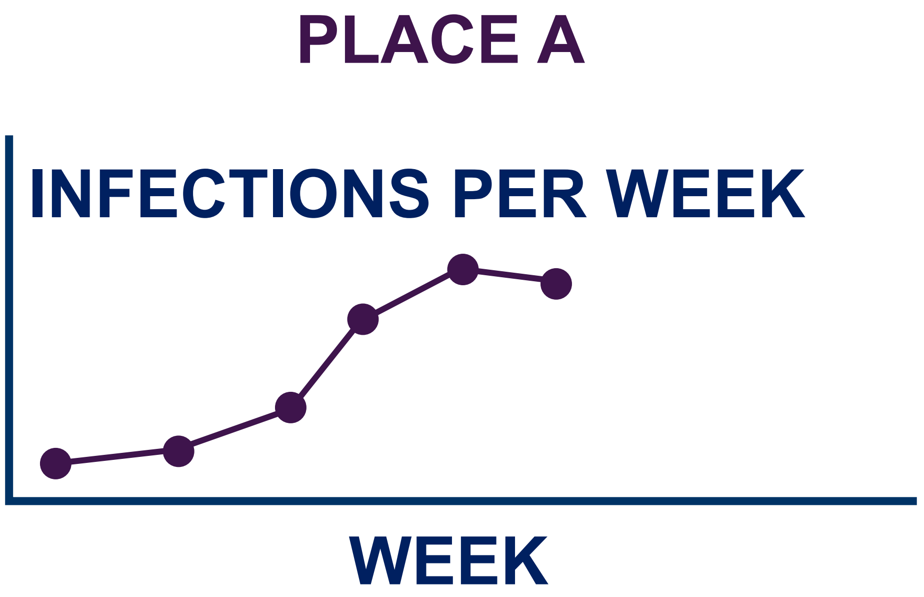

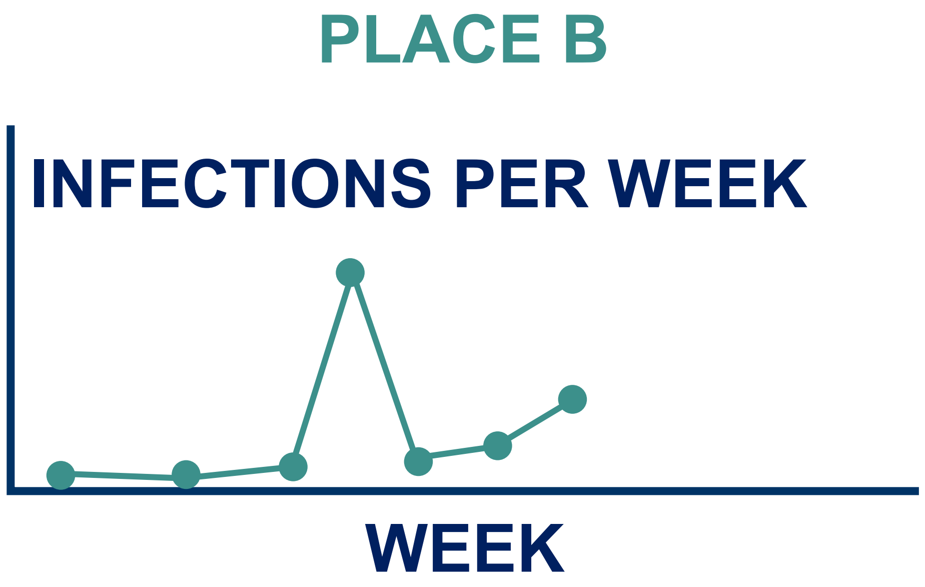

PROBLEMS WITH DATA

- ***What if data quality and quantity vary across locations, and over time?***

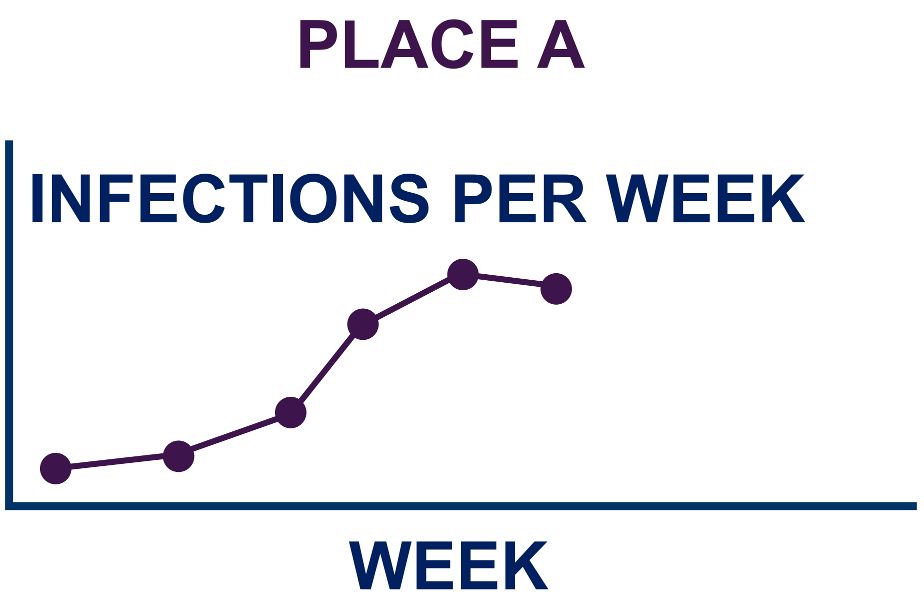

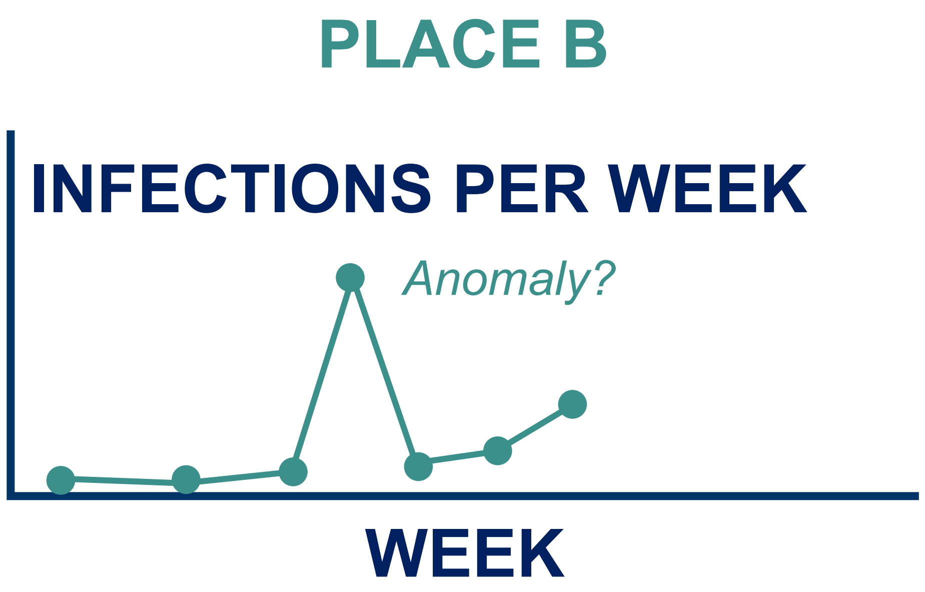

LOTS OF MODELS

- *Which model is best for my jurisdiction?*

SPATIAL SCALE OF MODEL PREDICTIONS

- *If predictions are made for a ‘state’, are these relevant for a ‘county’?*
- *How do we make predictions for a ‘city’, if data are only available at the scale of ‘state’?*

PROBLEMS WITH DATA

- *What if data quality and quantity vary across locations, and over time?*

Collaboration can lead to robust models that are more in tune to the evolving needs of public health experts.

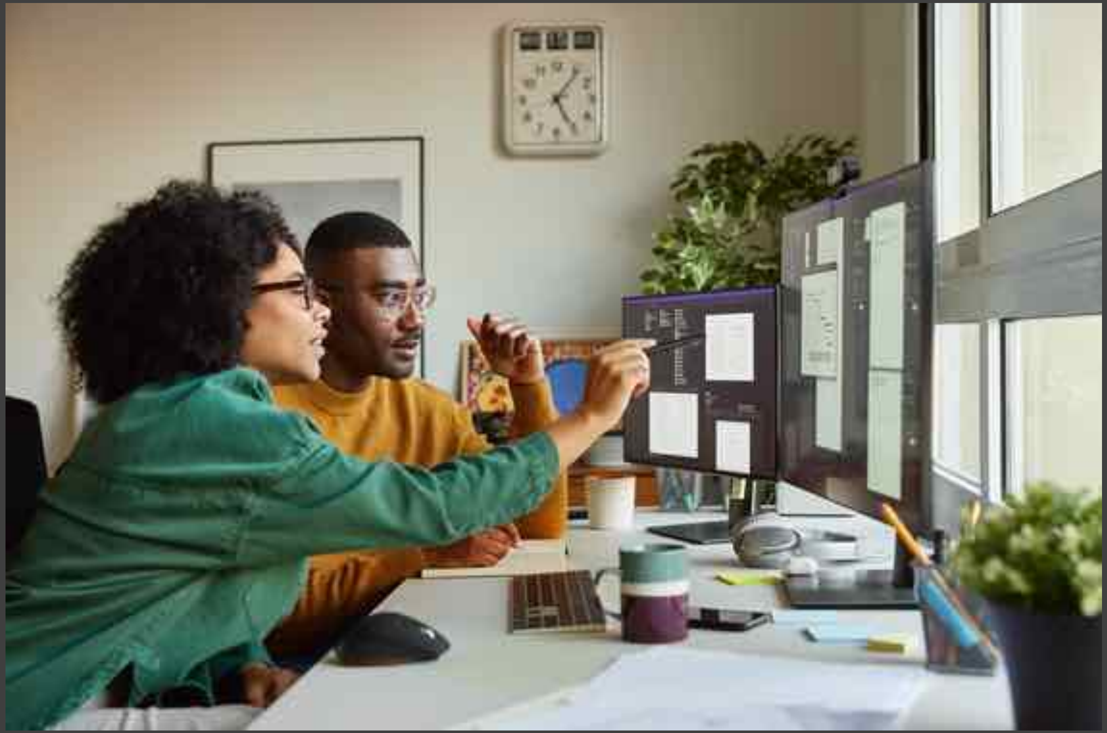

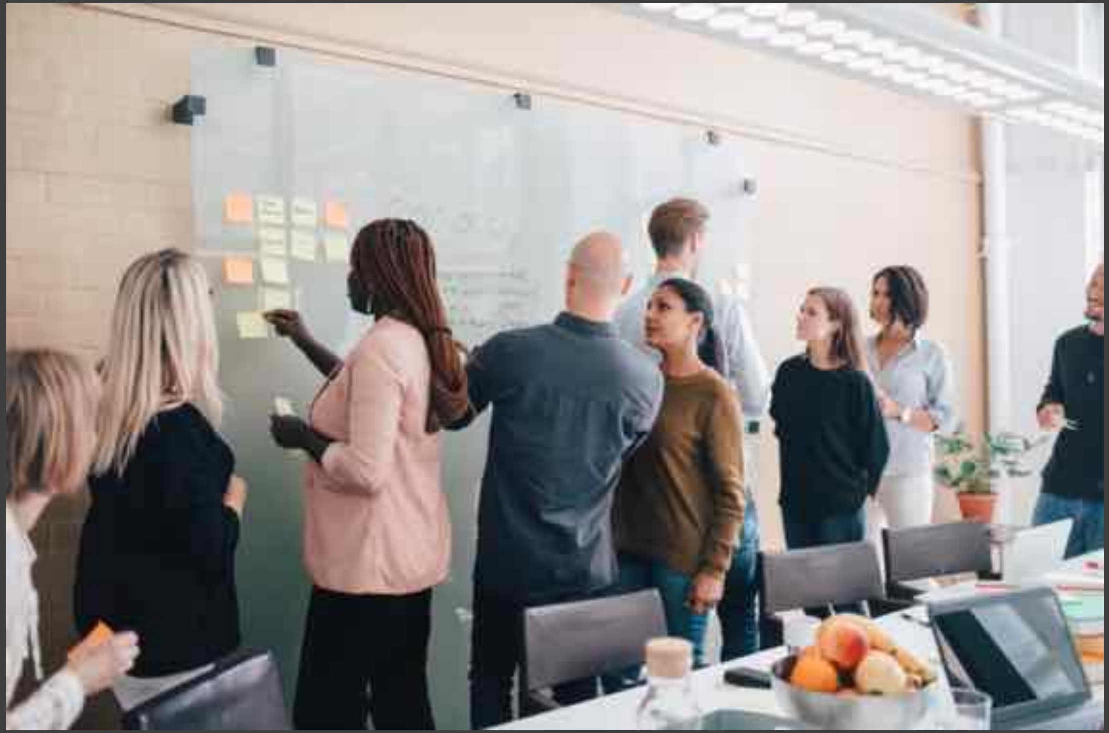

Breakout group discussions

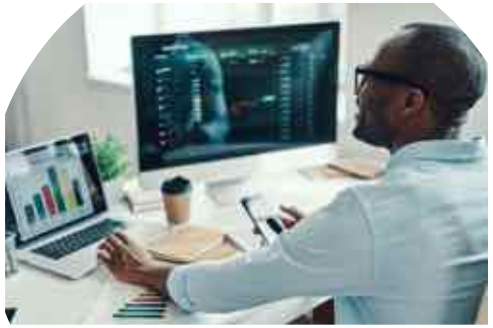

1. Infectious disease data and data analysis tools

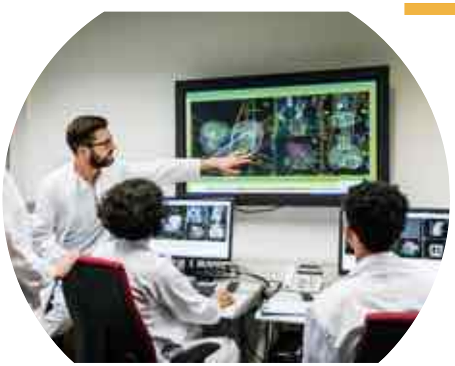

2. The role of data and technology in decision-making

EpiMoRPH
An Automated Epi Modeling Framework

3. Opportunities and challenges for using modeling technologies in Arizona
