# Supplementary material for: An inaugural forum on epidemiological modeling for public health stakeholders in Arizona
Source: Front Public Health. 2024 May 31;12:1357908. doi: 10.3389/fpubh.2024.1357908 (PMC11176426; doi:10.3389/fpubh.2024.1357908)
Supplement: Supplementary file 3 [file Data_Sheet_1.DOCX]

**Phase I: Infectious Disease Data and Data Analysis Tools**

| **Ques** | **Group 1** | **Group 2** | **Group 3** | **Group 4** | **Group 5** | **Group 6** | **Common** |
| --- | --- | --- | --- | --- | --- | --- | --- |
| **1. INTRODUCTION (not included here)** | | | | | | | |
| **2. DATA** | | | | | | | |
| 5. | What types of infectious disease data sets do you work with most frequently? | | | | | | |
|  | Survey, surveillance, prediction, forecast | Survey, surveillance, hospital dashboard, no direct involvement | Surveillance, case-based, case report, treatment | —--------------  (Surveillance, academic, applied) | COVID-19, Influenza, TB,  STDs , WNV, Dengue, Encephalitis. Congenital Syphilis, RSV. Chlamydia, Gonorrhea, Monkeypox. | (a lot of detail)  mosquito,  National, lat long coordinates, County, City, building level, Surveillance | Surveillance, case reports, academic research |
| 6. | What is the spatial scale of this data? | | | | | | |
|  | Local, county, state | Local, county, state | Individual points, state, and county | —--------------  (state) | County, Tribal | National, lat/long coordinates, County, City, building level, | National, State, County, Local |
| 7. | Do you wish you had different data to work with or data at a different spatial scale? | | | | | | |
|  | Yes, regional, district, unified data | Yes, tribe-specific data to avoid misclassification of demographics | Yes, missing data for acute cases, care points, and collaboration across the county, and state | —----------------- | Yes, SES and tribe-specific, SDOH specific | Understand the community, better indicator data, new data system to capture more cases, behavioral data, cohort data, representative data scales | Yes, local community specific |
| **3. DATA ANALYSIS** | | | | | | | |
| 8. | How are you involved with the analysis or visual presentation of infectious disease data? | | | | | | |
|  | Decision making, optimization | Surveillance, Hospital dashboard, Get Standardized guidance | Dashboards, reports, hot spot identification | —-------------------  (Surveillance, Reports) | Surveillance, Reporting, Stakeholders presentations, Dashboards | —------------------  (academic, researcher, Public Health Officials, Surveillance) | Surveillance, Hospital dashboard, Reporting |
| 9. | Are there educational opportunities to facilitate improved data literacy or data analysis literacy? | | | | | | |
|  | Online resources | Conferences, tribal epidemiology center | communication with community is difficult | —-------------------  (couldn't identify) | Varies for counties, somehow have LMS support some don't. | CDC Website, summer school | Varies, Not many opportunities |
| **4. DATA ANALYSIS AND MODELING TECHNIQUES** | | | | | | | |
| 10. | What tools or technologies have you used to analyze or visualize data? | | | | | | |
|  | Python, R, Javascript, Adobe, Plotly | SAS, R, Tableau,  SPSS | Tableau, Excel, ArcGIS, SAS, internal databases, R, python | Excel, R, Python | SAS, R, python, power bi, Esri solutions, my sidewalk, Excel, ESRI, Epi Info | —---------------- | SAS, R |
| 11. | What have been your experiences with epidemiological models (e.g. model forecasts)? | | | | | | |
|  | Optimization models, Case study generation intervention, SEIR | Not for everybody | Generally not good models, many factors that influence data, miscommunication, what after prediction | Great | Non-existent. all do summary statistics | Not clear | Varies, generally concerns about modeling |
| 12. | What are your opinions on how modeling analyses have been presented to you? | | | | | | |
|  | Robust, Interpretable, Redefining, Transparency in assumptions, multiple scenarios to accommodate uncertainty | Fair to not clear, challenging.  Miscommunication | Not well explained for real-life situations | —---------------- | subjective and conjecture, looks like a challenge while a lot of variation is involved | —----------------- | Generally miscommunication involved |
| 13. | What have you liked or not liked about using data analysis tools? | | | | | | |
|  | Like: summarization, not too steep a learning curve.  Dislike: not easy to interpret, not flexible, don't know the input data, tech issues | Like: simple interface  Dislike: Not easy to learn, not many resources | Dislike: sometimes the learning curve is too high, lot of variation, not a universal tool | —------------------ | Disease process will change but data collection doesn’t change, causes limitations | —---------------------- | Like: simple interface  Dislike: learning curve, not dynamic |
| 14. | What have you liked or not liked about how data analysis has been presented to you in the past? | | | | | | |
|  | Like: Good Prediction  Dislike: not interpretable, complicated plots, no clear context of the question | Dislike: such a lack of access to real-time data | Dislike: not linked to reality, no context, no explanation | —-----------------  (Get hold of real-time data is painful) | —------------------  (developed programs can be shared) | —------------------ | Like: prediction  Dislike: lack of accuracy to current scenarios |
| 15. | What are some questions or challenges you want to address, but you don’t feel like you have the capacity or right technologies available to you? | | | | | | |
|  | Selection of suitable model with a specific scenario, sensitive agent-based models, and of time series, covariates data, a centralized data platform | No real-time data, many models but no forecasting | Dislike: Big gaps in data quality and data speed | —-------------------- | Funding, staffing, data quality, fear of coming to the wrong conclusion and presenting the wrong outcome to the expert leadership | —------------------- | Data quality |
| **TAKEAWAY** | | | | | | | |
|  | Need for small spatial scale data, increase data quality, clarity of model assumptions, communication among stakeholders | —--------- | Rich Data, trust issues (community members), data quality and speed | Get hold of real-time data is painful, inferring missing data is time-consuming, need flexible models to accommodate multiple sources of data,  Researcher needs to understand the disease | —--------------  (collaboration for training, programming, code sharing) | Data collected in convenience can misled, Need operation data, resources, budget, scale, mitigation, Spatial scale, and heterogeneity, SOPs for Models | Issue of Data quality,  Speed, Scale, Training resources, Trust |

**Phase II: The Role of Data and Technology in Decision-Making**

| **Ques** | **Group 1** | **Group 2** | **Group 3** | **Group 4** | **Group 5** | **Group 6** | **Common** |
| --- | --- | --- | --- | --- | --- | --- | --- |
| **5. DECISION MAKING** | | | | | | | |
| 1. | What types of decisions do you make regarding infectious disease? | | | | | | |
|  | interpretation for stakeholders, for interventions, cross board communications | surveillance decision, risk, intervention decision | Interpreter not decision makers | Cluster identification, analysis, summary stats, resource allocation | bidirectional communication between county and state for intervention, impact assessments, equitable allocation of interventions | Informers and knowledge sharing | Information, interpretation to stakeholders |
| 2. | How is data and/or technology involved in your decision-making? | | | | | | |
|  | Linkage and standardization of data | capacity to build organizations develop their own models | Indirect data acquisition with trust issues | efficacy of solutions, evaluations | sustainable decision making, accommodate temporal/seasonal changes in population | get feedback from decision makers on what to explore, optimization of resource allocation | Recommendations, not decision making |
| 3. | Are there opportunities that you see to better integrate data in decision-making? | | | | | | |
|  | real time, at finer scales, Community-driven data, automation of data aggregation and anonymization | Having data ready for immediate decision, learn from their mistakes, data quality | Data privacy, cleaning, trust, SOP’s b/w data provider and modelers, centralization of system | Grouping cohesive data sets together, standardization in documentation, | Collaboration between modelers and data collectors at local level | Capturing human centered behaviors, timely data, | Timely data, standardization, trust building, centralization |
| 4. | What do you see as the resources that are most limited in terms of responding to infectious disease and making decisions about resource allocation? | | | | | | |
|  | Non-standardized, fragmented data, spatial scale, internet availability in rural areas | Funding, Experienced professionals, lack of real time data or any data at all, incomplete demographic data | —-------------- | Timely, reliable, accurate, ground level data, funding, workforce | quality data, scale and refinement, lack of data banks | Data privacy, timeliness, sharing and notification | Data quality, availability, standardization |
| 5. | Are there unique aspects about your jurisdictions that make it difficult to use currently available data? | | | | | | |
|  | access to technology, to interstate data, unique communities, anonymity issues, cross border dynamic populations. | less acceptance to CDC or University data | —--------------- | Access to data, non reporting by hospitals, undercoverage of specific populations | Access to data, availability and accuracy of data, staffing, expertise, | Density of data in rural areas, leadership support and innovation | Access, funding, workforce |
| 6. | Are there unique aspects about your jurisdictions that make it difficult to use technologies to inform your decisions? | | | | | | |
|  | —--------------  (cross border dynamic populations) | Trust on data sources | —---------------- | —---------------- | —------------------ | computing resources |  |
| **TAKEAWAY** | | | | | | | |
|  | —------------------ | —---------------- | Development of a trustable standardized infrastructure | Standardized common system,timely data and workforce, cultural sensitive intervention and building trust | Community specific data and collaboration b/w PH users, policy makers and models. | Focus on community perspective and capacity building rather than politics and power |  |

**Phase III: Opportunities and Challenges for Using Modeling Technologies in Arizona**

| **Ques** | **Group 1** | **Group 2** |  | **Group 3** | **Group 4** | **Group 5** | **Group 6** | **Common** |
| --- | --- | --- | --- | --- | --- | --- | --- | --- |
| **6. OPPORTUNITIES AND CHALLENGES FOR EPIDEMIOLOGICAL MODELING AND OTHER DATA ANALYSIS TOOLS** | | | | | | | | |
| 1. | What have you learned today about epidemiological modeling that may be useful to you in understanding or managing infectious disease across Arizona? | | | | | | | |
|  | Usefulness, applications of epi modeling | —--------------- |  | Resource allocation and disease update also incorporation of community | —----------------- | Seems promising for resource location but with certain limitations for prediction, like environmental data at a smaller scale is difficult to get. | model comparison across various datasets, data scarcity at local level,importance of inclusion of communities | Usefulness in resource allocation |
| 2. | How can resource allocation models be useful to you (e.g., making decisions about where to deploy certain resources)? | | | | | | | |
|  | Only policymakers can tell | —------------- |  | Need and impact assessment at community level | —----------------- | useful for preparedness and response programs, but policy dependent interventions is a constraint | resource allocation for specific communities, predictive power of models is not always accurate. | Community specific allocation |
| 3. | What challenges do you foresee with trying to build epidemiological modeling technologies for Arizona? | | | | | | | |
|  | Incorporate variation in climate and weather, small community data, standardization of data | —-------------- |  | lack of data, trust, managerial issues, data delay because of political issues | —------------------- | Data scarcity, standardization, sharing issues, budgeting issues | Established connections between modelers and decision makers, hiring workforce,data availability, communication | Data scarcity, standardization, collaboration |
| 4. | Do you feel like epidemiological modeling would add valuable information for decision-making? Why or why not? | | | | | | | |
|  | Yes, circumstances based on communities not politicians | ? |  | Yes, if practice on problems and appropriate resources allocation | —-------------------- | Useful for prediction and visualization, but inaccurate results will cause issues of trust and reliability in future | Need training to develop trust, connections and working relationships in tribes | yes |
| 5. | What spatial scales would models need to make predictions for them to be useful for decision-making? | | | | | | | |
|  | Depends on community and mode of transmission, preferably tribal level | Smaller scale |  | Depends on the question and needs | —------------------ | Zip code level | Various levels like neighborhood, zip code but can create administrative issues. | Zip code specific, small areas (particularly tribe specific) |
